# Optimization of Micropropagation and Metabolomic Analysis Under Different Light Qualities in *Mussaenda pubescens* Ait.f

**DOI:** 10.3390/plants14213268

**Published:** 2025-10-26

**Authors:** Li Sun, Jiajia Wu, Zilu Yang, Roudi Cai, Xiaoping Xu, Jiahui Li, Ning Tong, Muhammad Awais, Yuling Lin, Zhongxiong Lai

**Affiliations:** 1 Institute of Horticultural Biotechnology, Fujian Agriculture and Forestry University, Fuzhou 350002, Chinabyxxp310107@163.com (X.X.); awais9518@gmail.com (M.A.);; 2 Fujian Planting Technology Extension Station, Fuzhou 350003, China; 3 Biotechnology Institute, Fujian Academy of Agricultural Sciences, Fuzhou 350003, China

**Keywords:** *Mussaenda pubescens* Ait.f., in vitro regeneration, LED spectrum, metabolome profiling, secondary metabolites, bioactive metabolites, medicinal value

## Abstract

The current investigation utilized stem nodes from pre-established aseptic lines of *Mussaenda pubescens* as explants to optimize an efficient in vitro propagation protocol and investigated the effect of different light qualities (white, red, blue, and green) on metabolite accumulation in micropropagated plantlets. The findings demonstrated that the optimal medium for shoot proliferation was Murashige and Skoog basal medium supplemented with 6-Benzylaminopurine 2.0 mg·L^−1^ and α-naphthaleneacetic acid 0.2 mg·L^−1^, achieving a multiplication coefficient of 12.2 after 30 days. Rooting was more effective on Murashige and Skoog basal medium containing α-naphthaleneacetic acid 0.1 mg·L^−1^ and activated charcoal 1 g·L^−1^, resulting in a 100% rooting rate. During acclimatization, a substrate mixture of perlite:vermiculite: peat soil (1:1:1) promoted vigorous root development with a 100% survival rate at post-transplantation. Light quality significantly influenced plant morphology: red light stimulated stem elongation, while blue light increased biomass accumulation. Broad-target metabolomics revealed distinct metabolite profiles under different light spectra, with differentially accumulated metabolites primarily belonging to terpenoids, organic acids, lipids, and flavonoids. Specifically, red light enhanced the levels of terpenoids and lipids; blue light promoted the synthesis of specific triterpenoid saponins and lipids; while green light increased the content of certain terpenes and broadly upregulated a wide spectrum of lipids. This work provides a robust framework for the commercial micropropagation of *Mussaenda pubescens* and elucidates the strategic use of light quality to enhance the production of its valuable medicinal metabolites, including terpenoids and lipids.

## 1. Introduction

*Mussaenda pubescens* Ait.f. is a plant from the genus *Mussaenda* in the *Rubiaceae* family, known for its medicinal properties, and is primarily distributed in regions such as Guangdong, Fujian, and Hainan in China [1]. Its roots, stems, and leaves are rich in medicinal compounds such as terpenoids, flavonoids, and organic acids, which are recognized for their febrifugal, anti-inflammatory, and osteoprotective properties [2] and potential bioactivities. For instance, three monoterpenes (mussaenins A, B, and C) were isolated from the aerial parts of *M. pubescens* [3]. Research indicates that Mussaendoside O [4], a triterpenoid saponin isolated from *M. pubescens*, may have therapeutic potential against osteolytic bone disease. Additionally, a saponin analog extracted from *M. pubescens* leaves demonstrated anti-osteoclastogenic activity [5]. This plant has medicinal, ornamental, and ecological value, offering promising prospects for development and utilization. The intensifying demand for these plants has led to a significant decline in wild resources. The cultivation of *M. pubescens* faces significant constraints, primarily due to the protracted growth cycle of wild specimens, coupled with the inherent limitations of conventional propagation techniques. Specifically, both seed propagation and traditional cuttings fail to reliably preserve their desirable horticultural traits and are characterized by low efficiency. Studies on its breeding system indicate it is functionally dioecious, which might complicate natural regeneration and traditional propagation [6]. These challenges severely hinder large-scale production, failing to meet the market demand for stable, high-quality cultivars. Tissue culture technology offers a feasible solution to these constraints [7,8]. Although the genus *Mussaenda* comprises numerous species, tissue culture methodologies remain understudied. Previous preliminary research on *M. pubescens* tissue culture encountered various obstacles, such as low differentiation rates, extended growth cycles, and pronounced vitrification [9]. To comprehensively address these challenges, this study aimed to establish an efficient in vitro propagation system by optimizing culture conditions and plant growth regulator regimens. The prime objective was to enable efficient germplasm preservation, enhance the reproduction coefficient, and facilitate mass seedling production in a controlled environment.

Light is a crucial environmental factor that influences plant physiological functions and serves as a key source of signals that regulate plant growth and development [10]. Light quality differentially modulates the accumulation of secondary metabolites in plant species. The content of salvinorin I/H and salvinorin E in *Dracocephalum forrestii* under blue light (40 μmol·m^−2^ s^−1^) was 2.5 times higher than that in the control group [11]. Younas et al. [12] found that red light doubled total silymarin accumulation in *Silybum marianum* callus cultures compared to untreated groups. Tariq et al. [13] showed that green light treatment increased the total phenolic content, total flavonoid content and antioxidant activity of *Artemisia absinthium* callus tissues. Medicinal plants typically have basal accumulation levels of secondary metabolites, but their production can be significantly enhanced by strategic spectral optimization to increase their medicinal value. It has been well-documented that light quality regulates secondary metabolism in a variety of medicinal plant species. For instance, a study on Andrographis paniculata demonstrated that different spectral bands (red, blue, far-red, and ultraviolet light) significantly influence the accumulation of its principal active components, including andrographolide [14]. This established precedent provides a solid theoretical foundation for our investigation into the impact of light quality on the metabolic profile of *M. pubescens*.

Metabolomics can broadly describe shifts in plant metabolomes under different environmental conditions, thereby clarifying the metabolic pathways and regulatory mechanisms of metabolite synthesis. In recent years, metabolomics has been widely utilized for the identification and comprehensive investigation of the dynamic secondary metabolites in medicinal plants [15]. In particular, its power in elucidating the effects of light quality has been demonstrated in studies on medicinal plants. For example, broad-targeted metabolomics revealed that supplemental LED lighting specifically altered the accumulation of flavonoids and phenolic acids [16]. Supplemental LED light significantly altered metabolite accumulation in *Taxus wallichiana var. chinensis*, and metabolomic analysis exhibited a significant upsurge in benzenoid in the treated plants [17]. Metabolomics technology is a powerful detection tool for uncovering quantitative changes in metabolite accumulation in medicinal plants cultivated under specific environmental conditions, thus enabling a comprehensive analysis of their medicinal phytochemistry.

In recent years, research on *M. pubescens* has concentrated mainly on its chemical constituents and pharmacological effects, leaving its in vitro propagation poorly developed [2,18]. However, there have been limited inquiries into its in vitro propagation systems or light-quality-mediated metabolic regulation. In plant tissue culture, parameters such as basal medium, light, temperature and cytokinins are critical for regeneration and secondary metabolite production [19]. Unfortunately, suboptimal conditions often yield unsatisfactory outcomes—including slow growth, vitrification, and low differentiation—as reported in prior *M. pubescens* studies. Therefore, this work first established an efficient rapid propagation system by systematically optimizing culture conditions and plant growth regulator protocols. In addition, because light quality is a potent environmental regulator of secondary metabolism and has not been investigated in this species, we evaluated the impacts of white, red, blue, and green light on the growth and metabolome of tissue-cultured plantlets [20]. By utilizing broadly targeted metabolomics, we analyzed metabolite distributions and differences between treatment groups to elucidate the impact of light qualities on metabolite accumulation patterns and identify biologically active compounds. This study aimed to provide scientific foundation and technical support for the development and large-scale production of *M. pubescens*, as well as key insights into its pharmacological effects and the metabolic regulation mechanisms of medicinal plants.

## 2. Results

### 2.1. Establishment of Aseptic System and Optimization of Proliferation Medium

#### 2.1.1. Establishment of an Aseptic Line

Using stem nodes from pre-established aseptic strains cultured on Murashige and Skoog (MS) basal medium with 2.0 mg·L^−1^ 6-Benzylaminopurine(6-BA) and 0.2 mg·L^−1^ α-naphthaleneacetic acid (NAA). This protocol effectively reduced contamination to below 20.1% and achieved a survival rate exceeding 65.6%, with no secondary contamination observed in subsequent cultures, thereby delivering highly reliable aseptic materials for successive screening of proliferation, rooting, and strengthening media.

**Figure 1:**
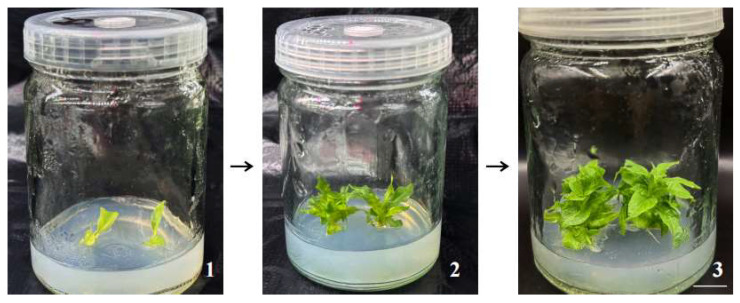
Growth process of *M. pubescens* on the optimal culture medium. Note: Explants were cultured on MS medium supplemented with 2.0 mg·L^−1^ 6-BA and 0.2 mg·L^−1^ NAA. The morphological phenotypes were observed and photographed at different culture periods: (1) 5 days, (2) 15 days, and (3) 30 days. The plants grew vigorously and maintained a healthy physiological status without any vitrification phenomenon. Scale bar = 1 cm.

The balance of NAA and 6-BA concentrations played a critical role in explant growth and development. Through systematic testing of different hormone combinations, the optimal medium for proliferation and rooting of *M. pubescens* was identified as MS basal medium supplemented with 2.0 mg·L^−1^ 6-BA and 0.2 mg·L^−1^ NAA. As illustrated in Figure 1, explants cultured on this medium exhibited vigorous growth with significantly reduced browning and no callus formation. The survival rate of explants increased remarkably, producing physiologically stable shoots suitable for subsequent culture stages.

#### 2.1.2. Optimization of Proliferation Medium: Impact of Hormonal Concentration Gradients on Mussaenda pubescens Shoot Multiplication

**Figure 2:**
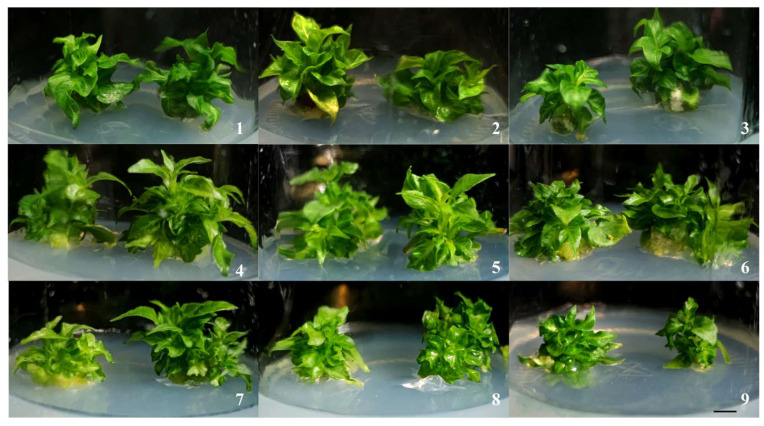
Effect of different plant growth regulator ratios on the proliferation of *M. pubescens* shoot clusters. Note: Rows share the same 6-BA concentration, and columns share the same NAA concentration. The plantlets in treatments 2, 5, and 8 exhibited good growth vigor, with those in treatment 5 being the most robust and showing the best overall growth status. In contrast, treatment 9 resulted in the poorest growth performance, producing dwarfed plantlets. Scale bar = 1 cm.

In the proliferation medium, the addition of different concentrations of 6-BA and NAA influenced the multiplication of *M. pubescens* (Table 1, Figure 2). Variations in hormone concentration significantly affected shoot multiplication and proliferation. At a constant concentration of NAA, an upsurge in 6-BA induced an initial increase followed by a decline in the multiplication coefficient, with a peak at 2 mg·L^−1^ of 6-BA. At a fixed concentration of 6-BA (2 mg·L^−1^), the variation in NAA produced the same trend, with the maximum coefficient being reached at 0.2 mg·L^−1^ of NAA. Plant height followed the same kinetics as the multiplication coefficient, with an optimum at 2 mg·L^−1^ of 6-BA + 0.2 mg·L^−1^ of NAA, with respective values of: multiplication coefficient: 12.20 ± 1.82 and average height: 2.07 ± 0.04 cm. However, at 3 mg·L^−1^ of 6-BA + 0.3 mg·L^−1^ of NAA, the following was observed: vitrification phenomenon, callus formation at the base of the shoots reduced performance (Coefficient: 10.70 ± 2.71, Height: 1.80 ± 0.05 cm). These results indicate that the optimal hormone ratio for tissue culture of *M. pubescens* is 6-BA 2 mg·L^−1^ + NAA 0.1–0.2 mg·L^−1^. This hormone ratio is favorable to the proliferation of shoot buds, and the outcomes indicate that there is an optimal concentration threshold for cell division.

### 2.2. Effect of Hormone Concentration on In Vitro Rooting of Shoots and Subsequent Plantlet Growth in Mussaenda pubescens

**Figure 3:**
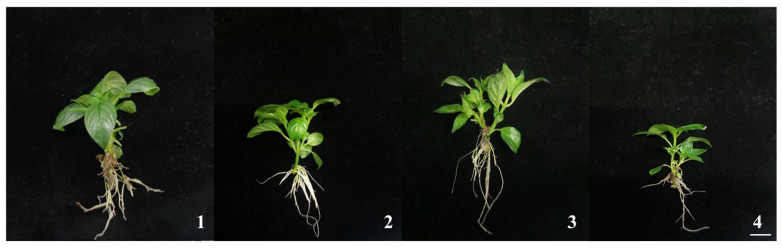
Effects of NAA on in vitro rooting and plantlet development of *M. pubescens*. Note: (1) MS + 0.05 mg·L^−1^ NAA: abundant but shorter roots with relatively lower plant height. (2) MS + 0.10 mg·L^−1^ NAA: optimal root development with the highest root number, robust root system, abundant lateral roots, and greater plant height. (3) MS + 0.15 mg·L^−1^ NAA: reduced root number and less vigorous root system, though plant height was moderately increased. (4) MS + 0.20 mg·L^−1^ NAA: poorest root performance with minimal, fragile, and brittle roots, accompanied by significantly reduced plant height.

As specified in Table 2, all tested NAA concentrations promoted adventitious root formation in *M. pubescens*, with root system morphology and plantlet development showing a clear correlation with NAA concentration. Specifically, as NAA concentration increased, the average number of roots in *M. pubescens* first increased and then decreased. At a concentration of 0.1 mg·L^−1^ NAA, the number of roots was highest (9.33 ± 1.85), significantly higher than that observed at other concentrations. At supraoptimal concentrations of 0.2 mg·L^−1^ NAA, root induction was minimized, and roots exhibited structural fragility, compromising ex vitro establishment. These results establish 0.1 mg·L^−1^ NAA as the optimal auxin concentration for simultaneous root morphogenesis and plantlet vigor in *M. pubescens*, demonstrating a defined efficacy threshold for auxin-mediated growth regulation (see Figure 3).

### 2.3. Effect of Different Substrates on the Acclimatization of Mussaenda pubescens Plantlets

**Figure 4:**
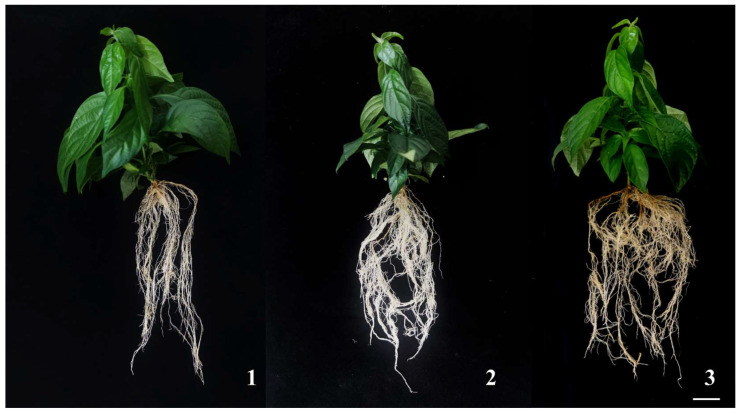
Comparison of tissue culture plantlets of goldenrod with different substrate treatments. Note: Growth phenotypes of *M. pubescens* plantlets under three substrate treatments. (1) Treatment 1 exhibited shorter plant height with elongated but weaker root systems. (2) Treatment 2 showed the thickest stems, prominent taproots, and more fibrous roots. (3) Treatment 3 resulted in greater plant height, yet with thinner stems and taproots, along with increased lateral root development.

Screening of transplant substrates (Table 3) exhibited that the perlite: vermiculite: peat soil = 1:1:1 formulation significantly optimized *M. pubescens* vitroplants, with a survival rate of 100%, which is considerably higher than the peat soil and perlite: vermiculite = 1:1 (93.3%). After 45 days post-transplantation (Figure 4), plantlets transplanted into perlite: vermiculite: peat moss = 1:1:1 presented the best growth, with the most vigorous root development, significantly more roots and longer root lengths than the other two treatment groups, and more distinct stem height growth. In addition, the leaves were dark green, and the plantlets were more robust. The perlite: vermiculite = 1:1 mixture demonstrated inadequate aggregate stability, causing substrate flotation during irrigation, which cooperated root-substrate contact and reduced survival to 93.3%. During watering, it inclined to float on the surface, leading to poor root contact and impaired root growth, which in turn exaggerated survival rates. Meanwhile, pure peat moss has insufficient air pockets and macroporosity and restricted aeration, hindering the formation of new roots in transplanted plantlets. These results indicate that the optimal substrate formulation for *M. pubescens* is perlite: vermiculite: peat moss = 1:1:1, achieving a transplant survival rate of 100% and accelerating hardening-phase completion.

### 2.4. Differential Regulation of Morphogenesis in Mussaenda pubescens Micropropagated Shoots by Light Quality

**Figure 5:**
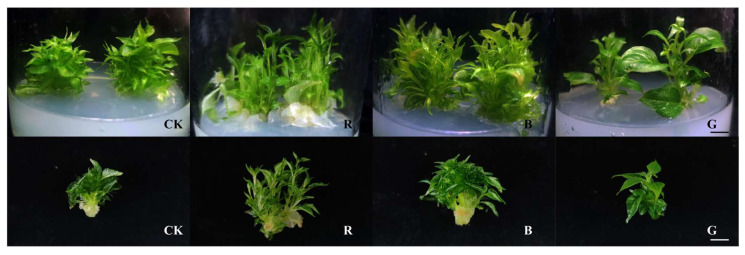
The phenotype of *M. pubescens* shoots cultured with different light qualities. Note: Treatments: CK (control), R (red light), B (blue light), G (green light). Red light enhanced stem elongation but reduced leaf thickness and area. Blue light strongly suppressed stem elongation, promoting thicker stems, shorter internodes, and increased leaf thickness. Green light reduced shoot height but increased leaf area.

**Figure 6:**
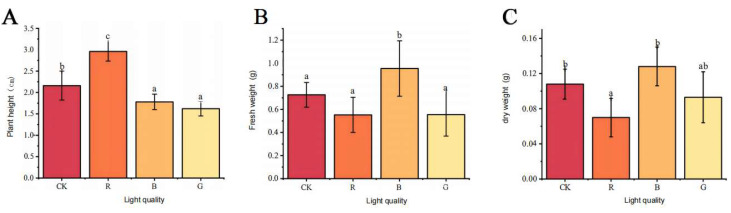
Growth parameters of *M. pubescens* shoots cultured with different light qualities. Note: (**A**) Plant height, (**B**) Fresh weight, (**C**) Dry weight. Treatments: CK (control), R (red light), B (blue light), G (green light). In (**A**), all light quality treatments showed significant differences compared with the CK. In (**B**), the blue light (B) treatment showed a significant difference compared with the CK. In (**C**), significant differences were observed in the red light (R) and blue light (B) treatments compared with the CK, while no significant difference was found in the green light (G) treatment. Data are presented as mean ± SD. Different letters indicate significant differences (*p* < 0.05, Tukey’s test).

Four different light quality treatments were applied to *M. pubescens* vitroplants for 30 days (Figure 5). The growth of *M. pubescens* tissue culture shoots under different light quality treatments significant photomorphogenic divergence compared to the white light control relative to white light controls. Red light treatment resulted in a significant reduction in leaf area, whereas blue light treatment maintained control-equivalent foliar dimensions in leaf area. In contrast, green light treatment significantly increased the leaf area. The growth parameters of tissue-cultured shoots of *M. pubescens* under different light quality treatments were measured, and the results and shown in Figure 6. Compared with the control group, the shoot height of *M. pubescens* tissue culture shoots significantly increased under red light treatment, whereas the plant height growth in the green and blue light treatment groups was lower than that of the control group. The blue light treatment group had a significantly higher fresh/dry weight increment, while the fresh/dry weight growth in the red and green light treatment groups was lower than that of the control group. The dry weight growth in the red light treatment group was lower than that in the green light treatment group, and the differences in fresh weight growth between the red and green light treatment groups were not significant.

### 2.5. Foliar Metabolic Profiling of Mussaenda pubescens Micropropagated Shoots Under Different Light Qualities

**Figure 7:**
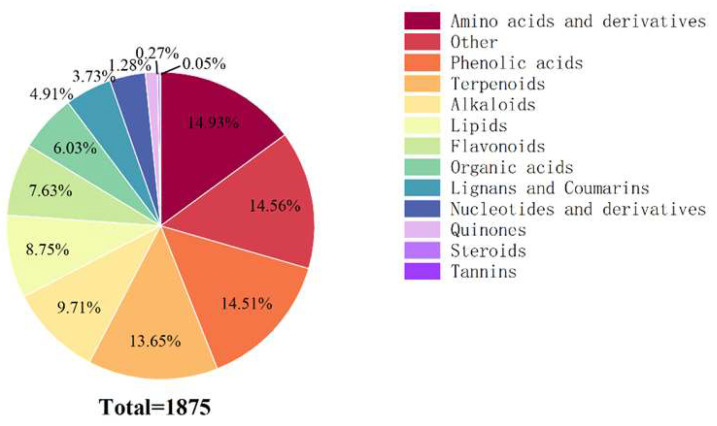
Classification of metabolites of *M. pubescens* with different light quality. Note: A total of 1875 metabolites were identified across all four light treatments (Control, R, B, G) and classified into 13 functional categories. The chart displays the distribution and relative abundance of these categories, revealing distinct metabolic patterns induced by specific light wavelengths.

Based on the KEGG classification system, a total of 1875 metabolites were identified across spectral treatment groups (Figure 7), distributed among 13 functional categories. Primary constituents are comprised. The main components, which included 280 amino acids and derivatives (14.93%), phenolic acids (272, 14.51%), and terpenoids (256, 13.65%). The next most abundant groups were alkaloids (182, 9.71%), lipids (164, 8.75%), and flavonoids (143, 7.63%). Remaining categories: organic acids (113 types, 6.03%), lignans and coumarins (92 types, 4.91%), nucleotides and derivatives (70 types, 3.73%), quinones (24 types, 1.28%), Steroids (5 types, 0.27%), and tannins (1 type, 0.05%).

**Figure 8:**
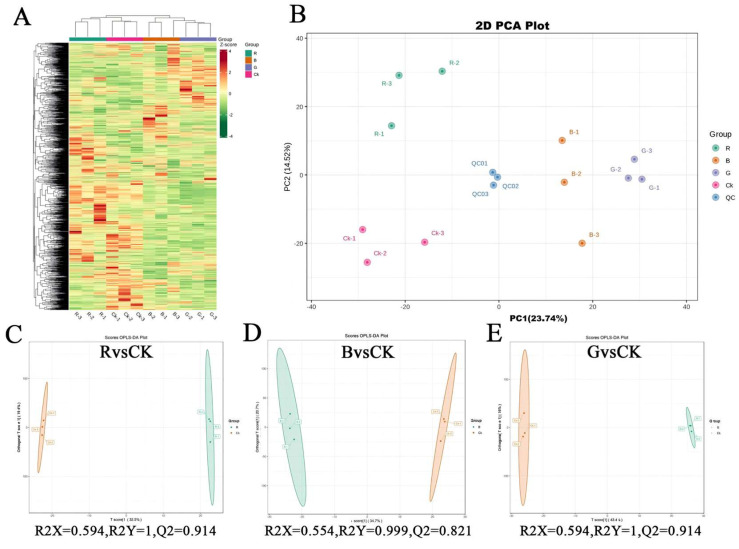
Statistical analysis of metabolites of different *M. pubescens* with different light quality. Note: Treatments are as follows: CK (control), R (red light), B (blue light), G (green light). (**A**) Clustering heat map of differentially abundant metabolites. (**B**) Principal component analysis (PCA) score plot. (**C**–**E**) Orthogonal partial least squares-discriminant analysis (OPLS-DA) score plots. These analyses reveal distinct metabolite profiles and effective separation among the four light treatment groups, indicating significant light-quality-induced metabolic alterations.

Cluster analysis was performed on metabolites from four groups of samples subjected to different light quality treatments. The results depicted (Figure 8A) that there were significant differences in the metabolite profiles among different groups, and biological replicates within each group were clustered tightly. This indicates that there are significant differences in the accumulation patterns of metabolites between groups, with high intra-group reproducibility and analytical reliability for differential metabolite identification.

Principal component analysis (PCA) was employed to evaluate intra- and inter-group metabolic divergence. The PCA (Figure 8B) demonstrated that the first two principal components (PC1 = 23.74%, PC2 = 14.52%) collectively accounted for 38.56% of total metabolic variance. The results suggest that the QC samples cluster together, indicating that QC samples share similar metabolic characteristics, and that biological replicates within each group are closely clustered, confirming the stability and reproducibility of the entire analysis. PCA results show a distinct chemotypic segregation among different light quality treatments, indicating significant changes in metabolite profiles among different treatments; specifically, B and G treatments are dispersed along the PC1 axis, while R and CK are significantly separated along the PC2 axis, with CK located in the negative quadrant of the PC2 axis. In summary, the outcomes of cluster analysis and principal component analysis consistently indicate that there are significant differences in the metabolic accumulation patterns of *M. pubescens* under different light quality treatments, and the leaves of the four treatments exhibit chemically distinct foliar profiles.

OPLS-DA (orthogonal partial least squares discriminant analysis) is a supervised multivariate statistical method that can separate variation signals unrelated to classification and identify discriminatory metabolites and differential metabolites. Pairwise OPLS-DA analysis was performed on the R, B, G, and Control groups, and score plots were constructed to visualize the separation trends between groups (Figure 8C–E). The results showed that the R group was significantly separated from the Control group (R^2^Y = 1.000, Q^2^ = 0.914), the B group was significantly separated from the Control group (R^2^Y = 0.999, Q^2^ = 0.821), and the G group was significantly separated from the Control group (R^2^Y = 1.000, Q^2^ = 0.914), where both R^2^ and Q^2^ scores were greater than 0.5 and tended towards 1, representing that the model had high fitting accuracy and was meaningful. Furthermore, based on the OPLS-DA results, variable importance in projection (VIP) was obtained to analyze and screen for differential metabolites.

### 2.6. Identification of Differential Metabolites in Leaves of Mussaenda pubescens Micropropagated Shoots Under Different Light Qualities

**Figure 9:**
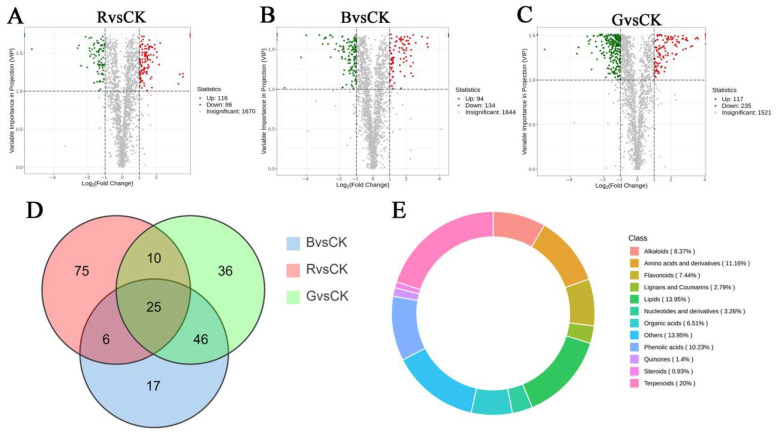
Volcano plots of different metabolites of *M. Pubescens* with different light quality. Note: (**A**–**C**) OPLS-DA score plots for paired comparisons: (**A**) Blue light vs. control (B vs. CK), (**B**) Green light vs. control (G vs. CK), and (**C**) Red light vs. control (R vs. CK). (**D**) Venn diagram showing the number of upregulated DAMs unique to and shared among the four light treatments. (**E**) Classification and statistical analysis of the upregulated DAMs across the four treatment groups.

DEMs (differentially expressed metabolites) were screened out based on FV and VIP, with results shown in Figure 9. A total of 228 differentially expressed metabolites were identified in Blue light vs. Control, counting 94 upregulated and 134 downregulated metabolites. In Green light vs. Control, a total of 352 DEMs were explored, including 117 up-regulated and 235 down-regulated, whereas in Red light vs. Control, a total of 202 differentially expressed metabolites were sorted out, together with 116 up-regulated and 86 down-regulated. The differentially expressed metabolites in the three groups were clustered primarily in four major categories: terpenoids, phenolic acids, organic acids, and lipids (Figure 9E). Particularly, blue light treatment significantly promoted the accumulation of phenolic acids (such as cimicifugic acid A and benzoic acid ethyl ester), with cimicifugic acid A being approximately 10 times higher(log_2_FC = 3.31) than in CK. Green light treatment significantly promoted the accumulation of flavonoids, lipids, phenolic acids, etc., such as cyanidin-3-*o*-rutinoside (log_2_FC = 2.91) and rosmarinic acid (log_2_FC = 1.68), with the expression of the lipid differential metabolite 9-hydroxyoctadeca-6,10,12,15-Tetraenoic Acid (log_2_FC = 3.61) being particularly significant, at approximately 12 times that of the control (CK). Red light treatment specifically induced the accumulation of metabolites, including 28 terpenoids, 19 lipids, 13 organic acids, 11 amino acid derivatives, and 10 phenolic acids. The most significant difference was observed in amino acid dicarboxylic acids (organic acids). Additionally, lipid expression was up-regulated after red light treatment, such as Sanleng acid (log_2_FC = 1.52)and 9-Oxooctadeca-10,12-octadecadienoic acid (log_2_FC= 1.77).

These findings indicate that the metabolic reprogramming of *M. pubescens* in response to different light qualities is consistent with photoreceptor regulatory mechanisms: red light preferentially stimulates primary metabolic pathways (e.g., terpenoid synthesis), while blue and green light enhance phenylpropanoid flux cryptochrome-mediated.

### 2.7. Analysis of Key Differential Metabolites in Leaves of Mussaenda pubescens Micropropagated Shoots Under Different Light Qualities

**Figure 10:**
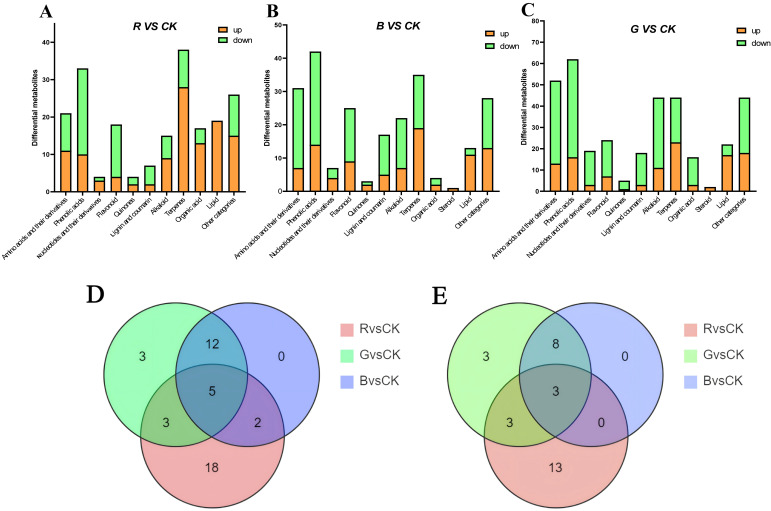
Statistical plots of different metabolites of *M. pubescens* with different light qualities. Note: (**A**–**C**) Bar plots showing the number of DAMs in pairwise comparisons: (**A**) Blue light vs. control (B vs. CK), (**B**) Green light vs. control (G vs. CK), (**C**) Red light vs. control (R vs. CK). (**D**) Venn diagram of upregulated differential metabolites across the four treatment groups. (**E**) Classification of upregulated differential metabolites in the four treatment groups.

To deep dive further into the differences in metabolite accumulation under various light conditions, we compared the main differentially expressed metabolites among the three treatment groups based on the identified differentially expressed metabolites. We found that light quality significantly altered the composition and content of terpenoid compounds in *M. pubescens* (Figure 10A–C), with red light significantly increasing the content of 28 types of terpene compounds, particularly the content of eucommoside, which was 3.28 times higher than that of the control group (CK). Blue light treatment promoted the accumulation of 19 types of terpenoids, including two triterpenoids (gynoside A and 3, 12, 23, 25-tetrahydroxy-20, 24-epoxydamaran-3-*o*-xylosylglucoside). Green light treatment increased the content of 23 types of terpenoids, significantly increasing the content of patrinioside and borneol 7-*o*-[β-ᴅ-furanosyl-(1→6)]-β-ᴅ-glucopyranoside.

Furthermore, light quality significantly altered the composition of lipids and organic acids in *M. pubescens*. In the red light treatment (Red light vs. Control), all 19 identified lipids and 13 organic acids were significantly upregulated. Key compounds such as aminopropionic acid and triacylglycerols showed relative contents 1.52 to 3.55 times that of CK, suggesting red light promotes glutamate dehydrogenase-mediated nitrogen redistribution. Blue light treatment (Blue light vs. Control) promoted lipid synthesis, with 11 out of 13 lipids significantly increased. The green light treatment (Green light vs. Control) induced the most substantial upregulation of lipids, significantly increasing the abundance of 17 lipids—including 9-hydroxyoctadeca-*6Z,10E,12E,15Z*-tetraenoic acid and LysoPC(19:2(2n isomer))—by 3.46 to 3.61-fold, while reducing the accumulation of organic acids like citric acid. A key finding regarding lipid metabolism was the distinct regulatory role of different light qualities. Specifically, red light promoted overall lipid accumulation, blue light enhanced lipid production, and green light treatment led to the most pronounced increase in lipid content.

In summary, our findings demonstrate that light quality treatments significantly alter the metabolic profile of *M. pubescens.* Notably, the coordinated accumulation of both terpenoids and lipids under red light suggests a potential rerouting of carbon flux at a central metabolic branch point. We hypothesize that the acetyl-CoA node, a common biosynthetic precursor for both metabolite classes, may be involved in this process. This hypothesis offers a plausible explanation for our observations and warrants further investigation. Blue and green light treatments promoted the enrichment of phenolic acids and flavonoids, while red light specifically drove the synthesis of terpenoids and lipids. Collectively, these results indicate that different light qualities can differentially steer carbon allocation into distinct secondary metabolic pathways.

### 2.8. KEGG Pathway Enrichment Analysis of Differential Metabolites in Mussaenda pubescens Micropropagated Leaves Under Different Light Qualities

**Figure 11:**
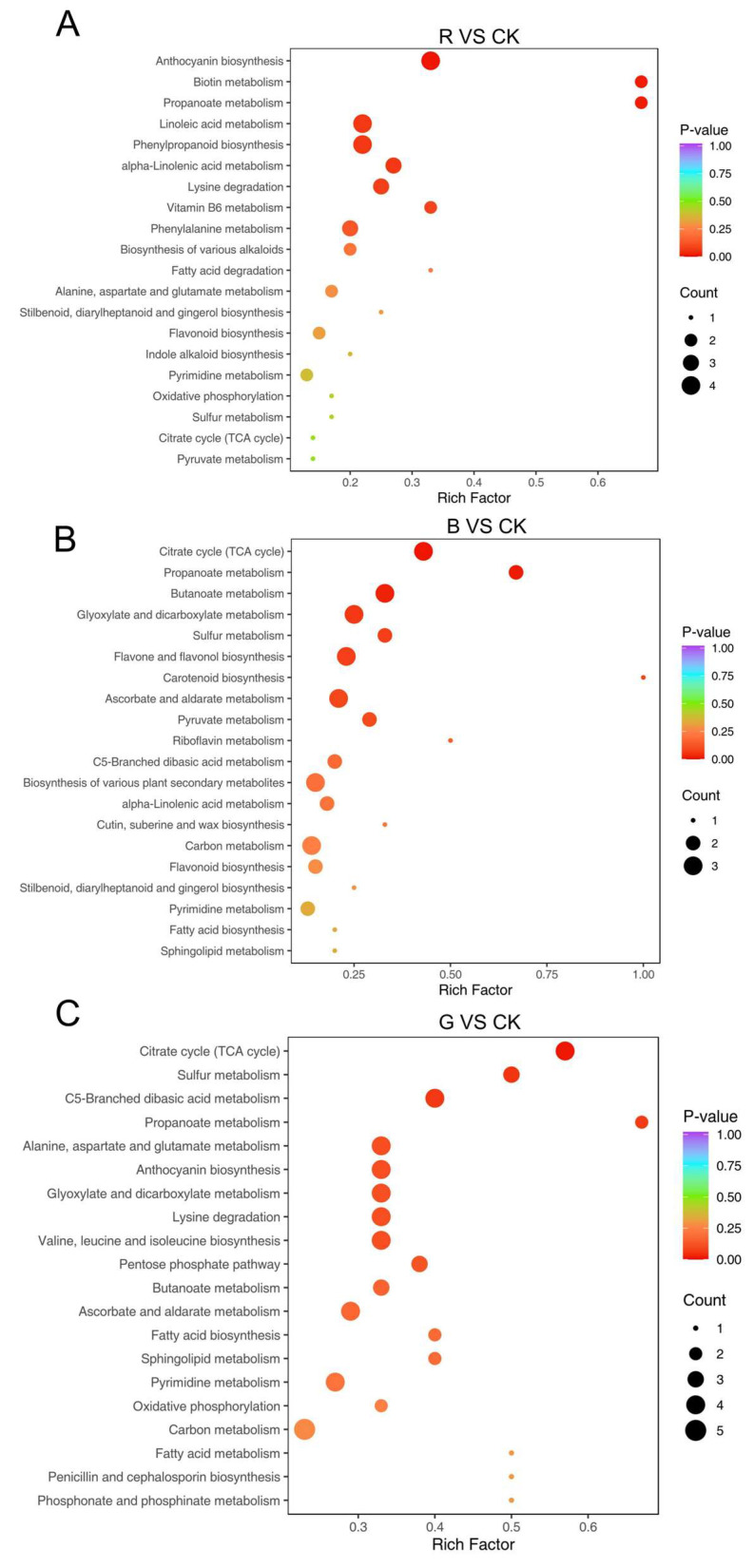
KEGG enrichment plots of different metabolites of *M. pubescens* with different light quality. Note: Bubble plots display the top significantly enriched pathways for pairwise comparisons: (**A**) blue light vs. control (B vs. CK), (**B**) green light vs. control (G vs. CK), and (**C**) red light vs. control (R vs. CK). The size and color of bubbles represent the number of differentially accumulated metabolites and the enrichment significance, respectively.

All differential metabolites across various comparison groups were assigned to KEGG pathways. A total of 405 metabolites were significantly enriched in 75 pathways, with 72.84% annotated to ‘Metabolic pathways’ (map01100) and 40.49% annotated to ‘Biosynthesis of secondary metabolites’ (map01110) (Table 4). Enrichment analysis of annotated pathways identified those significantly enriched with differential metabolites (*p* < 0.05) (Figure 11A–C), prominently including anthocyanin biosynthesis (map00942), zeatin biosynthesis (map00908), biotin metabolism (map00780), and propanoate metabolism (map00640). Under blue light treatment, carotenoid biosynthesis and anthocyanin synthesis were significantly enriched; Among the metabolites of carotenoid biosynthesis, dihydro-pterosinic acid (DPA) (log_2_FC = 2.41) was upregulated; the content of pelargonidin-3-*o*-(3″,6″-*o*-dipropionyl)glucoside, a metabolite of anthocyanin biosynthesis, was up-regulated, while the content of Cyanidin-3-*o*-(2″-*o*-glucosyl)glucoside and delphinidin-3-*o*-glucoside were down-regulated. Three differentially expressed metabolites were involved in flavonoid and flavonoid biosynthesis, including luteolin and kaempferol-3-*o*-rhamnoside (log_2_FC = 1.14), which were up-regulated, whereas luteolin-7-*o*-neohesperidoside (lonicerin) was down-regulated. Under green light treatment, anthocyanin biosynthesis, zeatin biosynthesis, and biotin metabolism were significantly enriched; Anthocyanin biosynthesis included cyanidin-3-*o*-rutinoside (log_2_FC = −2.87) and paeoniflorin-3-*o*-glucoside (log_2_FC = −2.02), both of which were downregulated. The zeatin pathway included uridine-5′-diphosphate-ᴅ-xylose (log_2_FC = −1.30) and adenosine triphosphate (log_2_FC = −1.71), both downregulated. Under red light treatment, biotin metabolism and propanoate metabolism were significantly enriched; in the propanoate metabolism pathway, succinic acid (log_2_FC = 3.54) and methylmalonic acid (log_2_FC = 3.25) were both up-regulated. The differential accumulation of metabolites in biotin metabolism and propanoate metabolism indicates that red light treatment promotes the accumulation of organic acid compounds.

Notably, all three comparison groups were highly enriched in the phenylpropanoid biosynthesis pathway and its downstream anthocyanin pathway. Different light qualities induced the expression of phenylalanine ammonia-lyase (PAL), promoting the accumulation of cinnamic acid upstream and thereby activating the accumulation of anthocyanin biosynthetic enzymes, eventually regulating anthocyanin accumulation. This highlights the crucial role of these pathways in the growth and development of *M. pubescens*.

## 3. Discussion

### 3.1. Development and Hormonal Regulation of an Efficient Micropropagation System for Mussaenda pubescens

The culture medium serves as the nutrients for the growth and development of tissue culture plant regeneration, whereas the types and concentrations of exogenous hormones play a critical role in axillary shoot proliferation and rooting quality, and the success of acclimatization and transplantation. Previous studies provide key evidence supporting the adoption of the axillary shoot proliferation approach in this research. For example, studies on Chinese yam (*Dioscorea opposita*) have shown that bud-bearing stem segments cultured on a suitable medium primarily facilitate the formation of multiple bud clusters, whereas segments without buds tend to induce callus formation [21]. Therefore, bud-bearing young stems of *M. pubescens* were selected as explants in this study, aiming to achieve the proliferation of cluster shoots by activating axillary buds, thereby maximally preserving the parental traits.

Previous study [22] has shown that the concurrent application of auxin and cytokinin exerts synergistic control over plant growth, development, and differentiation, thereby modulating the proliferation of clustered shoots. For instance, the established in vitro axillary shoot proliferation system in dwarf raspberry (*Rubus pubescens*) confirmed that cytokinins such as 6-BA and zeatin are key drivers for the germination and growth of axillary buds [23]. Specifically, the combination of 6-BA and NAA was found to promote the growth of axillary shoots, aligning with conclusions more effectively reported [24,25] on shoot proliferation formation in papyrus. Experimental results indicated that the 6-BA and NAA combination significantly enhanced axillary bud proliferation in *Clivia miniata*. Similarly, the addition of 1–2 mg·L^−1^ 6-BA and 0.1 mg·L^−1^ NAA to MS medium effectively promoted the formation of multiple bud clusters with 3–5 buds from bud-bearing stem segments of Chinese yam [26]. Likewise, a study on *Polygonum multiflorum* also confirmed that the combination of 6-BA (1.26–1.99 mg·L^−1^) and NAA (0.05–0.48 mg·L^−1^) optimally induced adventitious bud proliferation [27]. These studies collectively demonstrate that the combination of 6-BA and NAA can effectively promote axillary shoot proliferation, which is consistent with the findings of this study, confirming that this hormone combination significantly enhances axillary shoot proliferation in *M. pubescens.* As the concentration of 6-BA increased, shoot proliferation initially rose and subsequently declined. Elevated 6-BA levels induced vitrification in tissue-cultured *M. pubescens*, accompanied by limited callus formation at the base, which ultimately suppressed further shoot differentiation. These observations are consistent with reports by Shen et al. [28] and Li et al. [29], confirming that the 6-BA/NAA combination promotes cluster shoot proliferation while simultaneously stimulating callus generation throughout the culture period as 6-BA concentration increases. The optimal hormone ratio for proliferating clustered shoots of *M. pubescens* was determined to be 2 mg·L^−1^ 6-BA combined with 0.1–0.2 mg·L^−1^ NAA. Under these conditions, plants exhibited vigorous growth, high multiplication coefficients, and an absence of callus formation.

Throughout the rooting stage, the appropriate concentration of auxin (NAA) proved crucial for inducing root formation in tissue-cultured shoots. All treatments achieved a 100% rooting rate, indicating robust rooting competence. As rooting efficiency was not a constraint, subsequent evaluations emphasized root system quality and overall plantlet growth as key indicators for assessing rooting performance and identifying optimal culture conditions. Previous studies have identified NAA concentration as a primary factor governing root initiation in plant explants [30]. While suitable NAA levels promoted both proliferation and root growth, excessive concentrations inhibited explant development and rhizogenesis. The present study showed that at an NAA concentration of 0.1 mg·L^−1^, the rooting quality and morphology of *M. pubescens* tissue-cultured plantlets reached an optimum, yielding significantly greater root numbers and plantlet heights (plantlet height 3.46 ± 0.43 cm; average root number 9.33 ± 1.85) compared to other treatments. In contrast, at 0.2 mg·L^−1^ NAA, both plantlet growth and root number were suppressed (plantlet height 2.85 ± 0.11 cm; average root number 6.67 ± 0.67), which aligns with the findings that high auxin concentrations can inhibit organogenesis [31].

Acclimatization and transplantation of rooted tissue-cultured plantlets are critical phases that determine the overall success of rapid propagation systems. The survival rate and subsequent growth of plantlets post-transplantation are directly influenced by the choice of substrate, which must balance aeration and water retention to meet physiological requirements. Different transplant media led to varying survival rates among tissue-cultured plantlets. Experimental results demonstrated that a substrate mixture of perlite, vermiculite, and peat moss in a 1:1:1 ratio supported the highest survival rate (100%) for transplanted *M. pubescens*. This combination likely creates an ideal root zone environment by integrating the superior aeration provided by perlite and vermiculite with the high water- and nutrient-holding capacity of peat moss [32]. Such a balanced physicochemical property mitigates transplant shock by ensuring adequate oxygen supply to the roots while preventing waterlogging, thereby facilitating the observed robust root development and ultimately leading to a 100% survival rate and significantly enhanced plant height. This confirms that the specified substrate formulation enhances physicochemical properties, thereby facilitating robust and sustainable plantlet growth.

### 3.2. Light Quality Regulation of Morphogenesis in Mussaenda pubescens Micropropagated Shoots

Light quality, as a key environmental factor, plays a significant role in regulating plant growth and secondary metabolite accumulation [33,34,35]. This study demonstrated that red light treatment markedly promoted stem elongation in *M. pubescens* shoots, consistent with the growth-enhancing effects previously reported in *Sarcandra glabra* [36] and *Mesona chinensis* Benth [37]. This morphological alteration was underpinned by distinct metabolic reprogramming, specifically a marked upregulation of azelaic acid (Log_2_FC = 1.41), a recognized defense priming and signaling molecule [38]. According to Pashkovskiy et al. [39], red light induces phosphorylation and activates the plasma membrane H^+^-ATPase in guard cells. This process is mediated through the synergistic action of photopigment signaling and photosynthetic processes, with the latter also supplying the requisite ATP energy. Further observed that red light affects xylem cell development and upregulates genes involved in cytokinin and auxin signal transduction [40]. Based on these findings, we hypothesize that red light may enhance photosynthetic phosphorylation efficiency, accelerate the translocation of photoassimilates to the shoot apex, and upregulate the expression of cell wall loosening agents via the phytochrome signaling pathway, collectively promoting cell elongation. It should be noted that this promotive effect was primarily morphological, as the red light treatment simultaneously led to a decrease in shoot robustness and fresh weight.

In contrast, blue light influences plant photoperception and energy absorption, leading to increased leaf dry and fresh weights [41], with blue light treatment resulting in significantly greater biomass accumulation compared to the control. Our metabolomic data corroborate this by revealing a substantial accumulation of structural and functional lipids, most notably monogalactosyldiacylglycerol (MGDG, Log_2_FC = 2.95), a major galactolipid essential for chloroplast membrane integrity and photosynthetic function [42]. Stomatal responses to light include a specific blue light-induced reaction in guard cells [43]. Moreover, studies have shown that a large proportion of the carbon dioxide fixed by plants enters leaves through stomata [44]. It is thus hypothesized that blue light may enhance photosynthetic carbon assimilation by stimulating stomatal opening and improving CO_2_ uptake capacity, ultimately increasing leaf biomass.

Notably, green light treatment was found to increase leaf area in tissue-cultured *Crassula ovata* shoots, a trend consistent with observations in lettuce and potentially mediated by photosensory pigments [45]. We found green light treatment significantly promoted the accumulation of flavonoids and phenolic acids. Key metabolites included cyanidin-3-*o*-rutinoside (Log_2_FC = 2.91) and rosmarinic acid (Log_2_FC = 1.68), alongside a pronounced upregulation of the oxylipin 9-hydroxyoctadecatetraenoic acid (Log_2_FC = 3.61). This effect may be attributed to the greater penetration capacity of green light, enabling more efficient energy distribution within mesophyll tissues. Through interactions with photoreceptors, green light may delay leaf senescence and promote leaf expansion. Since fresh weight is a primary metric for evaluating the yield of medicinal plants, this study confirms that supplemental blue light significantly enhances biomass production in *M. pubescens*. This result clearly demonstrates that, unlike red light, which induced elongated but weaker growth, blue light was crucial for achieving compact morphology and substantial biomass accumulation. Therefore, integrating supplemental blue light into cultivation protocols is recommended to achieve high-quality, high-yield production in practical horticultural applications.

### 3.3. Red Light-Induced Metabolic Pathways for Bioactive Compound Synthesis in Mussaenda pubescens

Secondary metabolites synthesized by medicinal plants are a vital reservoir of natural bioactive compounds, and their biosynthetic processes are finely modulated by environmental cues. Light quality, as a key environmental factor, profoundly modulates the synthesis and accumulation of secondary metabolites such as terpenoids, phenylpropanoids, and alkaloids through activation of photoreceptor-mediated signaling networks. Light quality demonstrates dual regulatory specificity in regulating metabolites: different light qualities have differentially modulated the accumulation of different types of metabolites, and the same light quality can have varying effects on the same type of specialized metabolites, including in different plant species [46,47,48]. Numerous studies have confirmed that light quality significantly influences the accumulation of various secondary metabolites in plants. The identities and relative contents of the major terpenoids, lipids, and organic acids identified in the foliar tissues across all light treatments are provided in Supplementary Table S1.

Terpenoids are the most diverse class of plant metabolites, and *M. pubescens* contains a rich array of terpenoid compounds. Current research designates that terpenoids isolated from *M. pubescens* exhibit anti-inflammatory and antibacterial effects [49]. Different light qualities influence terpenoid synthesis, with red and blue light serving as effective regulators of terpenoid synthesis [50]. This study found that, compared to other light qualities, red light significantly promotes the synthesis and accumulation of terpenoid compounds in *M. pubescens*. Among the 28 significantly upregulated terpenoids under red light, key compounds included eucommoside (Log_2_FC = 3.28) and dihydrophaseic acid (Log_2_FC = 2.41), highlighting their role as major contributors to the red light-induced terpenoid profile. A previous metabolomic study on *M. pubescens* leaf development indicated that terpenoid accumulation is a hallmark of leaf maturation in this species, as terpenoid content increases naturally with maturation while phenolic acids and flavonoids decline [51]. Our findings suggest that red light treatment may mimic this maturity-related metabolic shift, effectively redirecting carbon flux into terpenoid synthesis during early tissue culture stages. This is consistent with the observed upregulation of 28 terpenoid compounds under red light in our study, highlighting the role of red light in modulating these core developmental metabolic pathways. Similarly, studies in *Artemisia annua* have shown that light-signaling cascades can intensify terpenoid content [52], a principle consistent with our findings in *M. pubescens*. Moreover, studies have shown that green light treatment can increase the content of ginseng ginsenosides Rg1 and Rf [53]. The results indicate that light quality regulation of terpenoid compound synthesis in medicinal plants exhibits significant species specificity, with red light specifically inducing the synthesis of the active component eucommoside(Log_2_FC = 3.28) in *M. pubescens.* Shao [54] reported that eucommoside may be a promising anti-neoplastic agent against breast carcinoma and also has regulatory effects on intestinal issues; Organic acids represent pharmacologically significant constituents distributed throughout the leaves, roots, and fruits of traditional Chinese herbs, such as chlorogenic acid and succinic acid in *Aconitum carmichaelii*. These compounds confer cardioprotective and immunomodulatory functions in the treatment of cardiovascular diseases and the protection of the human immune system [55]. The KEGG enrichment analysis in this study directed that the biotin metabolism pathway and propanoate metabolism pathway were significantly enriched under red light treatment. Further metabolic analysis confirmed that organic acid intermediates exhibited coordinated accumulation under red light, including heptanedioic acid, ᴅ-biotin, succinic acid, and carboxylic acid metabolism.

Results from other species are consistent with those of this study. It was found that red light enhanced the content of nine organic acids in the plant [56]; total organic acids in *Citrus sinensis* significantly increased under different light qualities (blue light, red light, white light, UVA, UVC). It is speculated that red light can specifically upregulate key genes in the organic acid synthesis pathway of *M. pubescens*. In addition to previously reported metabolites such as heptanedioic acid and ᴅ-biotin, aminopropionic acid accumulates significantly under red light, with its relative expression level than white light controls. Additionally, previous studies have shown that aminopropionic acid has certain anti-hyperglycemic effects on diabetes [57]. This indicates that red light, demonstrating 3.55-fold higher than that of the white light, indirectly promotes the synthesis of medicinal components but can also directly induce the accumulation of small molecules with clear pharmacological effects (aminopropionic acid), providing a new pathway for the development of the medicinal value of *M. pubescens*.

Lipids are an essential biomolecule compound in plants, critically regulating the plant growth cycles by providing the energy required for metabolism. Glycerophospholipids are one of the eight major lipid categories, and their metabolic products exhibit highly significant bioactivity, such as lysophosphatidylcholine (LPC). Previous studies have shown that LPC possesses antibacterial activity and can also enhance the antibacterial effect of antibiotics on bacterial strains [58]. Suh et al. [59] reported that red, blue, and red-blue joint light exposure repressed lipid accumulation in Perilla frutescens. Additionally, other studies have reported that red light upregulates the expression of many lipid biosynthetic genes in *Arabidopsis thaliana*. Metabolite results showed that red light promoted the accumulation of more lipid metabolites compared to blue and green light. LPC is involved in plant growth and development and regulates various physiological functions [60], underscoring the species-specific regulatory role of light quality. The functional significance of the observed lipid accumulation in *M. pubescens* and its potential role in mediating light-quality-induced physiological responses remains an important topic for future research.

### 3.4. Photoresponsive Signatures and Regulatory Potential of the Anthocyanin-Phenylpropanoid Biosynthetic Pathway in Mussaenda pubescens

The anthocyanin biosynthesis pathway is an important branch of the phenylpropanoid biosynthesis pathway, where light quality modulates anthocyanin accumulation can be regulated by stimulating anthocyanin biosynthesis genes. Anthocyanins are phenolic compounds, and research has shown that they possess anti-carcinogenic, anti-inflammatory, and anti-obesogenic properties, as well as significantly contribute to prevention in prevention of chronic human pathologies [61]. Shao et al. [62] found that both red light and blue light treatment can induce the phenylpropanoid, flavonoid, and anthocyanin metabolic pathways, actively regulating genes associated with the anthocyanin synthesis pathway. Red light stimulates the accumulation of anthocyanin content in rice by increasing the activity of peroxidase (POD), phenylalanine ammonia-lyase (PAL) [63]. Additionally, studies have shown that blue light further induces anthocyanin accumulation in plants by participating in the regulation of genes critical branch of phenylpropanoid metabolism [64]. These findings demonstrate that the phenylpropanoid biosynthesis pathway is the foundation for regulating the anthocyanin metabolic pathway. Based on KEGG enrichment analysis, we found that the phenylpropanoid biosynthesis pathway was significantly enriched under different light quality treatments, clearly indicating that light quality serves as an important environmental signal capable of effectively activating or reshaping the phenylpropanoid metabolic network in *M. pubescens*. Although the anthocyanin biosynthesis pathway also showed a certain enrichment trend in the enrichment analysis, the current metabolomics data have not provided evidence of light quality-regulated changes in the levels of anthocyanin synthesis intermediates or end products. It is speculated that light quality primarily modulates upstream regulatory nodes, midstream, or more fundamental regulatory nodes in the phenylpropanoid biosynthesis pathway. However, due to the complexity of anthocyanin synthesis, upstream regulation of anthocyanin synthesis by light quality may not directly or critically influence anthocyanin synthesis regulation. The enrichment signals in the anthocyanin pathway suggest that it may be indirectly influenced or have regulatory potential. The molecular mechanisms underlying the differential regulation of anthocyanin synthesis in *M. pubescens* under different light qualities require further investigation. Future studies could employ transcriptomics, targeted metabolomics, enzyme activity assays, and validation of key transcription factor functions to clarify the regulatory networks governing anthocyanin synthesis under different light qualities.

## 4. Materials and Methods

### 4.1. Materials

The experimental materials in this study were collected from Guling, Guanxi Town, Fuzhou City (119.3° E, 26.09° N). All media were supplemented with 30 g·L^−1^ sucrose and 7 g·L^−1^ agar. All media were adjusted to pH 5.8 using a 1 mol·L^−1^ NaOH solution and autoclaved at 121 °C for 20 min. All culture conditions were maintained at 25 °C, with 12 h of light and 12 h of darkness.

### 4.2. Sterilization of Explants

Stems of *M. pubescens* were soaked in washing powder for 4 h, and the washed explants were disinfected with 75% alcohol for 30 s on an ultra-clean bench and washed with sterile water 3–5 times. They were disinfected again with 0.1% HgCl_2_ for 8 min, and then explants were washed with sterile water five times, then drained and inoculated in bud induction medium: MS + 6-BA 4.0 mg·L^−1^ + Kinetin (KT) 0.5 mg·L^−1^ + NAA 0.1 mg·L^−1^ [65].

### 4.3. Screening of Optimal Medium for Axillary Shoot Proliferation

Induced sterile shoots were used as experimental materials for the proliferation culture. Uniform nodal segments, each containing an axillary bud, were selected and inoculated into MS medium supplemented with different concentrations of 6-BA (1, 2, and 3 mg·L^−1^, respectively) and NAA (0.1, 0.2, and 0.3 mg·L^−1^, respectively). Nine treatments were established using an orthogonal design. Each treatment was inoculated into 15 culture bottles, with two nodal segments per bottle. The growth of axillary shoots was observed, and the coefficient of proliferation of clustered shoots was counted after 30 d. Additionally, the plant height of the *M. pubescens* shoot was measured.

### 4.4. Effects of Different Media on Rooting of Shoots and Plantlet Growth

The plant materials used for the 30-day culture period were in vitro-derived shoot segments (approximately 2 cm in length), each containing a single nodal bud, obtained from the established aseptic lines of *M*. *pubescens*. These shoot clusters were separated into individual shoots and inoculated onto MS medium supplemented with NAA at concentrations of 0.05, 0.10, 0.15 and 0.20 mg·L^−1^. Four treatments were set up, and activated charcoal at 1 g·L^−1^ was added to each treatment. Each treatment was inoculated with 20 vials, with three replicates. Root formation was assessed after 45 days of incubation.

### 4.5. Screening for Optimal Substrate for Transplanting

For acclimatization and transplanting, well-rooted plantlets with robust leaf growth were selected. The plantlets were first pre-cultured in a greenhouse at 25 °C with a photosynthetic photon flux density (PPFD) of 100 μmol m^−2^ s^−1^ under a 12/12 h (light/dark) photoperiod for approximately 5 days. Subsequently, the medium adhering to the roots was then gently rinsed off with clean water, and the plantlets were transplanted into 10.5 cm diameter plastic pots containing sterilized cultivation substrate. Three different proportions of substrates were prepared: (1) peat soil; (2) Perlite: vermiculite = 1:1; (3) Perlite: vermiculite: peat soil = 1:1:1. Fifteen transplants were transplanted in each treatment with three replications. When transplanting, a layer of sterile polyethylene film was placed on the transplanting pots to achieve the effect of maintaining 80–90% relative humidity and lightly misted every 3 days to keep the substrate moist but not waterlogged. Survival rate and growth were assessed after 45 d.

### 4.6. Different Light Quality Treatments

The explants used consisted of in vitro-derived shoot segments (approximately 1.0–1.5 cm in length), each containing at least one nodal bud, obtained from established aseptic lines of *M. pubescens*. LED plant growth lamps (Model: T5-1.2M-18W-220V; Manufacturer: Boyue, Shenzhen, China) were used as the light source, and an LED light source emitting white light was used as the control (CK). The following light conditions were set: red light (R, peak emission at 630 nm with a full width at half maximum (FWHM) of ±22 nm), blue light (B, peak emission at 453 nm FWHM of ±19 nm), and green light (G, peak emission at 522 nm with a FWHM of ±35 nm), and the photosynthetic photon flux density (PPFD) was set at 100 μmol m^−2^ s^−1^. The emission spectra for all light sources are provided in Supplementary Figure S1. After 30 d of culture under their respective light conditions, leaves and stems from the four treatment groups were used as experimental materials. Three biological replicates were set for each treatment, and leaves and stems were snap-frozen in liquid nitrogen and stored in a refrigerator at −80 °C for later use.

### 4.7. Analysis by Ultra-Performance Liquid Chromatography-Tandem Mass Spectrometry (UPLC-MS/MS)

The sample was freeze-dried under vacuum in a lyophilizer (Scientz-100F, Ningbo, China) and ground (30 Hz, 1.5 min) to a powder using a grinder (MM 400, Retsch, Haan, Germany). Approximately 50 mg of the powder was accurately weighed and extracted with 1.2 mL of 70% methanol. The sample was vortexed every 30 min for 30 s, for a total of six times. The sample was placed in a refrigerator at 4 °C overnight. After centrifugation at 12,000 rpm for 3 min, the supernatant was collected, filtered through a microporous filter membrane (0.22 μm pore size), and stored in an injection bottle for UPLC-MS/MS analysis.

Liquid phase conditions: column: SB-C18 1.8 µm, 2.1 mm × 100 mm; mobile phase: ultra-pure water containing 0.1% formic acid in phase A, and ethylene glycol containing 0.1% formic acid in phase B; elution gradient: the proportion of B-phase was 5% at 0.00 min. The elution gradient was 5% of B phase at 0.00 min, the proportion B phase increased linearly to 95% at 9.00 min and was maintained at 95% for 1 min, the proportion of B phase decreased to 5% at 10.00–11.10 min, and equilibrated at 5% for 14 min; the flow rate was 0.35 mL·min^−1^; the column temperature was 40 °C; the injection volume was 2 μL.

The mass spectrometry conditions were as follows: electrospray ionization (ESI) at 500 °C; ion spray voltage (IS) 5500 V in positive ion mode/-4500 V in negative ion mode; ion source gas I (GSI), gas II (GSII), and curtain gas (CUR) were set to 50, 60, and 25 psi, respectively, and collision induced ionization was set to high. Triple Quadrupole (QQQ) scans using Multiple Reaction Monitoring Mode (MRM) mode (MRM metabolite detection multi-peak diagram shown in Supplementary Figure S1), with collision gas (nitrogen) set to medium.

### 4.8. Data Analysis

#### 4.8.1. Data Processing for In Vitro Fast Propagation Systems

Proliferation coefficient = total number of shoots after proliferation culture/total number of inoculated explants
Average plant height = total shoot height/total number of shoots
Rooting rate = number of rooted explants/number of inoculated explants × 100%
Average number of roots = total number of roots of all plantlets/total number of plantlets
Mean stem thickness = total stem thickness of shoots/total number of shoots

All data were subjected to one-way analysis of variance (ANOVA) using Excel and SPSS (version 23.0, IBM Corp., Armonk, NY, USA). Significant differences among means were determined by Tukey’s honestly significant difference (HSD) test at a significance level of *p* < 0.05. Data are presented as the mean ± standard deviation (SD). Figures were prepared using Excel and Origin (version 2021, OriginLab Corporation, Northampton, MA, USA) software.

#### 4.8.2. Widely Targeted Metabolomics Analysis

Metabolite characterization was performed using secondary spectrum information from the self-constructed database MWDB (Myvi Bioscience and Technology Co., Ltd., Wuhan, China). The metabolites were quantified by triple quadrupole mass spectrometry (TQMS) in multiple reaction monitoring (MRM) mode. The peak area of the metabolites was integrated after obtaining the mass spectra, and the mass spectra were corrected for the integration of the peaks out of the mass spectrometry. The peak area of each chromatographic peak represented the relative content of the corresponding substance. The identified metabolites were subjected to multivariate statistical analysis to explore the metabolite profiles of the four sample groups. According to the variable importance in projection (VIP) obtained by orthogonal partial least squares discriminant analysis (OPLS-DA), the metabolite profiles were analyzed in the following way importance in projection (VIP) score obtained by orthogonal partial least squares discriminant analysis model (OPLS-DA), the metabolites with VIP ≥ 1 and difference multiplicity value (FC ≥ 2 or FC ≤ 0.5) were defined as differential metabolites, and at the same time, the corresponding differential metabolites obtained were submitted to the Kyoto Encyclopedia of Genes and Genomes (KEGG) database website for pathway correlation analysis.

## 5. Conclusions

This study established a systematic framework for in vitro rapid propagation of *M. pubescens* and, for the first time, integrated broad-targeted metabolomics to investigate the regulatory effects of light quality on its metabolic network. The findings demonstrated that the optimal proliferation medium for shoot induction from nodal explants was: MS + 6-BA 2.0 mg·L^−1^ + NAA 0.2 mg·L^−1^, yielding a proliferation coefficient of 12.20 ± 1.82. The most suitable medium for rooting and subsequent plantlet growth was: MS + NAA 0.1 mg·L^−1^ + activated carbon 1 g·L^−1^, producing plantlets with a height of 3.46 ± 0.43 cm and 9.33 ± 1.85 roots per plant. The optimal transplanting medium is: perlite: vermiculite: peat moss = 1:1:1 (survival rate 100%). Different light quality treatments significantly influenced the phenotypic and metabolic profiles of *Mussaenda pubescens*. Blue light markedly promoted biomass accumulation, indicating its suitability for yield-oriented cultivation. Red light specifically enriched medicinal active components such as terpenoids, organic acids, and alkaloids, while also stimulating stem elongation. Green light acted synergistically with blue light to enhance lipid and partial terpenoid synthesis while increasing leaf area. This study elucidated the phenotypic and metabolic accumulation patterns of *M. pubescens* under different light qualities, providing a mechanistic foundation for its sustainable development and utilization. Furthermore, the established micropropagation protocol offers a scalable framework for the conservation and commercial production of this species, which can be adapted for the other members of *Mussaenda* genus.

## Figures and Tables

**Table 1:** Effects of different phytohormone combinations on shoot cluster proliferation in *M. pubescens*.

Treatment Serial No.	Hormone Concentration (mg/L)		Value-Added Factor	Average Plant Height (cm)	Growth	
	6-BA	NAA			Growth Momentum	Callus
1	1	0.1	10.30 ± 2.56 a	1.86 ± 0.08 bc	**	+
2	1	0.2	11.20 ± 3.14 ab	1.89 ± 0.03 c	**	+
3	1	0.3	11.30 ± 2.68 ab	1.85 ± 0.04 bc	**	+
4	2	0.1	11.80 ± 2.89 ab	1.99 ± 0.03 d	***	+++
5	2	0.2	12.20 ± 1.82 b	2.07 ± 0.04 f	***	+++
6	2	0.3	10.20 ± 2.78 a	1.95 ± 0.07 d	***	+
7	3	0.1	11.50 ± 2.52 ab	1.75 ± 0.03 a	**	++
8	3	0.2	11.40 ± 2.06 ab	1.82 ± 0.02 bc	**	++
9	3	0.3	10.70 ± 2.71 ab	1.80 ± 0.05 ab	*	

Note: Data are presented as mean ± standard deviation (SD). *, **, and *** indicate poor, moderate, and vigorous growth performance, respectively. +, ++, and +++ represent minor callus formation, leaf dedifferentiation, and little to no callus formation, respectively. Different lowercase lettersdenote significant differences among groups according to Tukey’s honestly significant difference (HSD) test (*p* < 0.05). The same conventions apply to the figures and tables below.

**Table 2:** Effects of different NAA concentrations on in vitro rooting and plantlet development of *M. pubescens*.

Treatment Serial No.	Hormone Concentration (mg/L) NAA	Average Plant Height (cm)	Rooting Rate (%)	Average Number of Roots
1	0.05	3.11 ± 0.45 ab	100	7.55 ± 0.70 ab
2	0.10	3.46 ± 0.43 b	100	9.33 ± 1.85 b
3	0.15	3.26 ± 0.20 b	100	8.44 ± 0.51 ab
4	0.20	2.85 ± 0.11 a	100	6.67 ± 0.67 a

Note: Data are presented as mean ± SD. Labels 1–4 represent different NAA concentrations: (1) 0.05 mg·L^−1^, (2) 0.10 mg·L^−1^, (3) 0.15 mg·L^−1^, and (4) 0.20 mg·L^−1^. The same numbering convention applies to the figures below. Where a, b, and c indicate significant differences between groups, *p* < 0.05.

**Table 3:** Effect of different substrates on the acclimatization of *M. pubescens* in vitro plantlets.

Treatment Serial No.	Substrate for Transplanting	Average Plant Height (cm)	Average Stem Thickness (cm)	Survival Rate (%)
1	peat soil	8.55 ± 0.47 a	0.164 ± 0.009 b	96.7
2	perlite: vermiculite = 1:1	9.25 ± 0.88 ab	0.18 ± 0.021 b	93.3
3	perlite: vermiculite: peat soil = 1:1:1	12.37 ± 1.76 b	0.12 ± 0.013 a	100

Note: Numbers 1–3 represent different substrate formulas: (1) Peat soil; (2) Perlite: Vermiculite = 1:1; (3) Perlite: Vermiculite: Peat soil = 1:1:1. Plant height, stem diameter, and survival rate were measured after 45 days of culture. Data are presented as mean ± SD. Where a, b, and c indicate significant differences between groups, *p* < 0.05.

**Table 4:** Top 10 statistics of KEGG enrichment analysis of differential metabolites in *M. pubescens* under different light quality treatments.

KEGG Level 1	KEGG Pathway	KoID	Metabolome Frequency
Metabolism	Metabolic pathways	ko01100	72.84%
Metabolism	Biosynthesis of secondary metabolites	ko01110	40.49%
Metabolism	Biosynthesis of cofactors	ko01240	13.83%
Metabolism	Biosynthesis of amino acids	ko01230	12.59%
Environmental Information Processing	ABC transporters	ko02010	11.11%
Metabolism	2-Oxocarboxylic acid metabolism	ko01210	7.65%
Metabolism	Nucleotide metabolism	ko01232	5.93%
Metabolism	Carbon metabolism	ko01200	5.43%
Metabolism	Biosynthesis of various plant secondary metabolites	ko00999	4.94%
Metabolism	Linoleic acid metabolism	ko00591	4.44%

Note: The table lists the top 10 most significantly enriched KEGG pathways (*p* < 0.05) ranked by metabolome frequency, showing the pathway classification, name, KEGG Orthology identifier (KoID), and the percentage of differentially accumulated metabolites mapped to each pathway.

## Data Availability

The authors declare that the data supporting the findings of this study are available within the paper and its Supplementary Materials files. Should any raw data files be needed in another format, they are available from the corresponding author upon reasonable request. Source data are provided with this paper.

## Supplementary Materials

The following supporting information can be downloaded at: https://www.mdpi.com/article/10.3390/plants14213268/s1, Identification of the total metabolites under different light quality of *M. pubescens* (Table S1), DEGs-based KEGG metabolic pathway enrichment in comparison of B vs. CK and GvsCK and RvsCK (Tables S2–S4), and this part was submitted to the document separately. Supplementary Figure S1: Emission spectra of the LED light sources used in this study. Figure S2: MRM Metabolite Detection Multi-Peak Chart.

## Abbreviations

R	red light
B	blue light
G	green light
OPLS-DA	orthogonal partial least squares discriminant analysis
VIP	variable importance in projection
ESI	electrospray ionization
PPFD	photosynthetic photon flux density
d	days
6-BA	6-Benzylaminopurine
NAA	1-naphthaleneacetic acid
MS	Murashige and Skoog basal medium
HSD	honestly significant difference
ANOVA	one-way analysis of variance
*M. pubescens*	*Mussaenda pubescens*
KT	Kinetin
FWHM	full width at half maximum
DEM	differentially expressed metabolites
MRM	Multiple Reaction Monitoring
