# Supplementary material for: Optimization of Micropropagation and Metabolomic Analysis Under Different Light Qualities in Mussaenda pubescens Ait.f
Source: Plants (Basel). 2025 Oct 26;14(21):3268. doi: 10.3390/plants14213268 (PMC12610484; doi:10.3390/plants14213268)

Supplemental Data Table:

Table S1. Identification of the total metabolites under different light quality of *Mussaenda Pubescens*

Code	Molecular weight (Da)	Formula	Compounds	Class
1	3.27E+02	C18H17NO5	N-Caffeoylphenylalanine	Amino acids and derivatives
2	1.66E+02	C8H6O4	Terephthalic acid*	Phenolic acids
3	2.10E+02	C12H18O3	(+)-7-iso-Jasmonic acid	Organic acids
4	1.26E+02	C5H6N2O2	Imidazole-4-Acetic Acid*	Alkaloids
5	3.66E+02	C17H22N2O7	N-(1-Deoxy-1-fructosyl)Tryptophan	Amino acids and derivatives
6	3.10E+02	C14H18N2O6	γ-Glutamyltyrosine	Amino acids and derivatives
7	5.38E+02	C25H30O13	Fraxamoside	Terpenoids
8	1.19E+02	C4H9NO3	L-Homoserine*	Amino acids and derivatives
9	1.19E+02	C4H9NO3	L-Threonine*	Amino acids and derivatives
10	1.69E+02	C8H11NO3	Pyridoxine	Others
11	1.78E+02	C9H6O4	Daphnetin	Lignans and Coumarins
12	2.92E+02	C18H28O3	9-Hydroxyoctadeca-6,10,12,15-Tetraenoic Acid	Lipids
13	1.78E+02	C11H15NO	5-Acetyl-2,3-dihydro-6,7-dimethyl-1H-pyrrolizi ne	Alkaloids
14	1.30E+02	C5H6O4	Citraconic acid	Organic acids
15	2.64E+02	C14H20N2O3	N-Feruloylputrescine	Alkaloids
16	1.47E+02	C5H9NO4	O-Acetylserine	Amino acids and derivatives
17	1.49E+02	C6H7N5	1-Methyladenine	Nucleotides and derivatives
18	7.40E+02	C33H40O19	Luteolin-7-O-rutinoside-5-O-rhamnoside	Flavonoids
19	2.38E+02	C8H14O8	Mucic acid Dimethyl Ester	Organic acids
20	4.72E+02	C30H48O4	2,3-Dihydroxylup-20(29)-en-28-oic acid (Alphitolic acid)*	Terpenoids
21	1.39E+02	C6H5NO3	4-Nitrophenol	Phenolic acids
22	3.33E+02	C18H39NO4	2-Aminooctadecane-1,16,18,18-tetraol	Others
23	1.32E+02	C4H8N2O3	3-Ureidopropionic Acid	Organic acids
24	2.78E+02	C10H18N2O5S	γ-glutamylmethionine	Amino acids and derivatives

25	2.78E+02	C18H30O2	α-Linolenic Acid*	Lipids
26	2.40E+02	C15H28O2	Cis-10-Pentadecenoic Acid(C15:1)	Lipids
27	3.16E+02	C14H20O8	5-(2-Hydroxyethyl)-2-O-glucosylphenol	Phenolic acids
28	1.92E+02	C10H8O4	Scopoletin (7-Hydroxy-6-methoxycoumarin)*	Lignans and Coumarins
29	1.26E+02	C5H6N2O2	Imidazol-1-yl-acetic acid*	Alkaloids
30	2.66E+02	C17H30O2	8,11-Heptadecadienoic acid	Lipids
31	1.48E+02	C6H12O4	Mevalonic acid	Organic acids
32	4.70E+02	C30H46O4	3-Hydroxy-11-oxours-12-en-28-oic acid (11-Keto-ursolic acid)	Terpenoids
33	4.58E+02	C22H18O11	p-Coumaroylcaffeoyltartaric acid	Phenolic acids
34	1.29E+02	C6H11NO2	Homoproline	Amino acids and derivatives
35	2.40E+02	C16H32O	Palmitaldehyde	Lipids
36	5.34E+02	C27H34O11	Phillyrin	Lignans and Coumarins
37	6.26E+02	C27H30O17	Myricetin-3-O-rutinoside	Flavonoids
38	2.96E+02	C18H32O3	9(10)-EpOME;(9R,10S)-(12Z)-9,10-Epoxyoctade cenoic acid	Lipids
39	1.17E+02	C5H11NO2	L-Valine*	Amino acids and derivatives
40	8.81E+01	C4H12N2	Putrescine	Alkaloids
41	1.61E+02	C6H11NO4	DL-2-Aminoadipic acid*	Alkaloids
42	2.61E+02	C14H31NO3	2-Aminotetradecane-1,11,13-triol	Others
43	1.80E+02	C11H18NO+	Candicine	Alkaloids
44	1.32E+02	C5H12N2O2	D-Ornithine	Amino acids and derivatives
45	2.96E+02	C18H32O3	alpha-Hydroxylinoleic acid*	Lipids
46	1.74E+02	C6H14N4O2	L-Arginine	Amino acids and derivatives
47	1.66E+02	C9H10O3	3-Hydroxyphenylacetic Acid Methyl Ester	Phenolic acids
48	4.22E+02	C27H34O4	7-Hydroxycoumarinyl-gamma-linolenate	Lipids
49	2.26E+02	C13H22O3	6,9-Dihydroxy-7-megastigmen-3-one	Terpenoids
50	4.05E+02	C22H47NO5	2-aminodocoSane-1,6,19,20,21-pentaol	Others
51	6.75E+02	C35H66NO9P	1-(9Z-Octadecenoyl)-2-(9-oxo-nonanoyl)-sn-gly cero-3-phosphocholine	Lipids
52	3.52E+02	C21H36O4	2-α-Linolenoyl-glycerol*	Lipids
53	2.47E+02	C10H21N3O4	Lys-Thr	Amino acids and derivatives
54	4.70E+02	C30H46O4	Rubianol-g*	Terpenoids
55	5.04E+02	C18H32O16	D-Panose*	Others
56	5.04E+02	C18H32O16	Raffinose*	Others
57	1.62E+02	C9H6O3	4-Hydroxycoumarin	Lignans and Coumarins

58	3.46E+02	C16H26O8	Kankanoside P	Terpenoids
59	1.45E+02	C6H11NO3	4-Acetamidobutyric acid	Organic acids
60	2.44E+02	C11H16O6	Shanzhigenin methyl ester	Terpenoids
61	1.81E+02	C9H11NO3	L-Tyrosine*	Amino acids and derivatives
62	9.56E+02	C47H72O20	Medicagenic acid-3-O-glucuronide-28-O-rhamnosyl(1,2)-arabinoside	Terpenoids
63	1.94E+02	C6H10O7	D-Galacturonic acid*	Others
64	1.75E+02	C6H13N3O3	L-Citrulline	Amino acids and derivatives
65	9.31E+01	C6H7N	2-Picoline; 2-Methylpyridine	Alkaloids
66	3.86E+02	C17H22O10	1-O-Eudesmoylquinic acid	Phenolic acids
67	3.30E+02	C18H34O5	Sanleng acid	Lipids
68	6.07E+02	C17H27N3O17P2	Uridine 5'-diphospho-N-acetylglucosamine	Nucleotides and derivatives
69	3.48E+02	C22H36O3	Anacardic acid	Organic acids
70	6.98E+02	C31H38O18	Gentistraminoside A	Terpenoids
71	3.30E+02	C18H34O5	9,10,13-Trihydroxy-11-Octadecenoic Acid	Lipids
72	3.36E+02	C16H16O8	5-O-Caffeoylshikimic acid	Phenolic acids
73	1.92E+02	C6H8O7	Isocitric Acid	Organic acids
74	2.34E+02	C15H22O2	Dihydrocostunolide	Terpenoids
75	1.47E+02	C6H13NO3	2-Amino-4-hydroxy-3-methylpentanoic acid	Amino acids and derivatives
76	3.03E+02	C12H21N3O6	Nicotianamine	Alkaloids
77	3.21E+02	C20H35NO2	linolenoylethanolamine	Others
78	1.24E+02	C7H8O2	4-Hydroxybenzyl Alcohol	Phenolic acids
79	4.62E+02	C21H18O12	Vnilloylcaffeoyltartaric acid	Phenolic acids
80	1.43E+02	C5H9N3O2	Cyclocreatine	Others
81	1.74E+02	C10H6O3	Lawsone; 2-Hydroxy-1,4-Naphthoquinone	Others
82	2.05E+02	C11H11NO3	(S)-3-(2-oxopropyl)-3-hydroxyindolin-2-one	Alkaloids
83	1.31E+02	C6H13NO2	6-Deoxyfagomine	Alkaloids
84	1.11E+02	C4H5N3O	Isocytosine	Nucleotides and derivatives
85	1.84E+02	C5H15NO4P+	O-Phosphocholine	Alkaloids
86	1.67E+02	C10H17NO	N-(2-Methylpropyl)hexa-2,4-dienamide	Others
87	3.86E+02	C17H22O10	4-O-Glucosyl-sinapate	Phenolic acids
88	3.56E+02	C16H20O9	4-O-β-D-glucopyranosylferulic acid	Phenolic acids
89	1.29E+02	C6H11NO2	N-Methyl-L-proline	Amino acids and derivatives
90	3.40E+02	C15H16O9	Daphnin*	Lignans and Coumarins
91	1.32E+02	C5H8O4	2-Hydroxy-2-methyl-3-oxobutanoic acid*	Organic acids
92	3.52E+02	C21H36O4	Monolinolenin*	Lipids

93	1.60E+02	C7H12O4	Pimelic acid*	Organic acids
94	2.02E+02	C11H10N2O2	vasicinone	Alkaloids
95	1.90E+02	C7H14N2O4	2,6-Diaminooimelic acid	Organic acids
96	1.94E+02	C10H10O4	6-Hydroxymellein	Others
97	5.04E+02	C30H48O6	1,2,3,19-Tetrahydroxyurs-12-en-28-oic acid	Terpenoids
98	1.28E+02	C5H8N2O2	5,6-Dihydro-5-methyluracil	Nucleotides and derivatives
99	1.38E+02	C7H6O3	Salicylic acid	Phenolic acids
100	1.18E+02	C5H10O3	β-Hydroxyisovaleric acid	Organic acids
101	5.17E+02	C26H48NO7P	LysoPC 18:3(2n isomer)	Lipids
102	4.72E+02	C30H48O4	3,19-Dihydroxyurs-12-en-28-oic acid (Pomolic acid)*	Terpenoids
103	3.70E+02	C20H18O7	9-hydroxysesamin	Lignans and Coumarins
104	1.18E+02	C4H6O4	Succinic acid*	Organic acids
105	1.80E+02	C11H16O2	Dihydroactinidiolide	Others
106	5.00E+02	C25H24O11	Gnaphaffine A	Others
107	5.09E+02	C25H52NO7P	LysoPC 17:0	Lipids
108	2.17E+02	C9H19N3O3	Lys-Ala	Amino acids and derivatives
109	1.03E+02	C4H9NO2	γ-Aminobutyric acid	Organic acids
110	3.38E+02	C16H18O8	3-O-p-Coumaroylquinic acid*	Phenolic acids
111	3.60E+02	C16H24O9	7-Deoxyloganic acid	Terpenoids
112	1.36E+02	C7H8N2O	6-Methylnicotinamide	Alkaloids
113	6.10E+02	C27H30O16	Luteolin-7-O-gentiobioside	Flavonoids
114	2.19E+02	C9H17NO5	6-(((S)-1-carboxyethyl)amino)-4-hydroxyhexan oicacid*	Amino acids and derivatives
115	1.38E+02	C7H6O3	2,5-Dihydroxybenzaldehyde	Others
116	3.52E+02	C21H36O4	Glycerol 9(E),11(Z),13(E)-octadecatrienoyl ester*	Lipids
117	3.50E+02	C21H34O4	15-Hydroxysessilifol F	Terpenoids
118	2.73E+02	C16H35NO2	2-Aminohexadecane-1,4-diol*	Others
119	4.02E+02	C18H26O10	Benzyl B-Primeveroside*	Phenolic acids
120	1.96E+02	C11H16O3	epiloliolide*	Others
121	1.98E+02	C11H18O3	Hydroxydihydrobovolide	Others
122	4.16E+02	C19H28O10	2-Phenylethyl beta-primeveroside	Phenolic acids
123	2.02E+02	C8H18N4O2	ethyl-L-arginine	Amino acids and derivatives
124	3.16E+02	C13H16O9	2-Hydroxy-5-[3,4,5-Trihydroxy-6-(Hydroxymeth yl)Oxan-2-Yl]Oxybenzoic Acid	Others
125	3.48E+02	C22H36O3	Viteagnusin B	Terpenoids
126	4.88E+02	C30H48O5	3,19,23-Trihydroxyurs-12-en-28-oic acid (Rutundic acid)	Terpenoids
127	4.70E+02	C30H46O4	3,6-Dihydroxyurs-12,18(19)-dien-28-oic acid	Terpenoids

			(Uncargenin A)	
128	3.58E+02	C19H18O7	Eupatorin-5-methylether (3'-hydroxy-5,6,7,4'-tetramethoxyflavone)	Flavonoids
129	3.28E+02	C15H20O8	Demethyl coniferin	Phenolic acids
130	2.72E+02	C16H32O3	(R)-Beta-Hydroxypalmitic Acid	Lipids
131	1.22E+02	C6H6N2O	Nicotinamide	Others
132	1.15E+02	C5H9NO2	1-Amino-1-cyclobutane-carboxylic-acid*	Amino acids and derivatives
133	2.82E+02	C18H34O2	Elaidic Acid*	Lipids
134	5.07E+02	C24H29NO11	Pyridoxine-5'-O-(6''-feruloyl)glucoside	Others
135	5.70E+02	C34H50O7	2,3-Diacetoxy-18-hydroxyoleana-5,12-dien-28-oic acid	Terpenoids
136	2.46E+02	C10H18N2O5	γ-Glutamyl-L-valine	Amino acids and derivatives
137	6.63E+02	C21H27N7O14P2	Nicotinic acid adenine dinucleotide	Nucleotides and derivatives
138	1.62E+02	C6H10O5	3-Hydroxy-3-methylpentane-1,5-dioic acid	Amino acids and derivatives
139	4.86E+02	C30H46O5	Cannabifolin D	Terpenoids
140	2.73E+02	C16H35NO2	Hexadecylsphingosine	Lipids
141	1.03E+02	C4H9NO2	N-Ethylglycine*	Amino acids and derivatives
142	3.52E+02	C21H36O4	1-α-Linolenoyl-glycerol*	Lipids
143	1.62E+02	C9H6O3	7-hydroxy-2H-1-benzopyran-2-one	Lignans and Coumarins
144	1.30E+02	C6H14N2O	N-Acetylputrescine	Alkaloids
145	3.28E+02	C16H24O7	3-Hydroxy-4-isopropylbenzylalcohol-3-O-glucoside	Phenolic acids
146	2.05E+02	C11H11NO3	3-Hydroxy-3-acetonyloxindole*	Alkaloids
147	2.16E+02	C10H20N2O3	Val-Val	Amino acids and derivatives
148	3.86E+02	C22H26O6	Pinoresinol dimethyl ether*	Lignans and Coumarins
149	2.27E+02	C14H29NO	N-Isobutyl Decanamide	Alkaloids
150	3.56E+02	C21H40O4	1-Oleoyl-Sn-Glycerol	Lipids
151	1.79E+02	C6H5N5O2	Isoxanthopterin	Nucleotides and derivatives
152	1.52E+02	C8H8O3	2-Hydroxyphenylacetic acid	Organic acids
153	2.40E+02	C14H8O4	Danthron; 1,8-Dihydroxyanthraquinone	Quinones
154	3.30E+02	C14H18O9	2-β-D-Glucopyranosyloxy-5-hydroxy-phenylacetic acid	Phenolic acids
155	1.42E+02	C8H14O2	2-n-Propyl-3-pentenoic acid	Organic acids
156	4.88E+02	C30H48O5	2,3,23-Trihydroxyurs-12-en-28-oic acid*	Terpenoids
157	3.32E+02	C13H16O10	Gallic acid-4-O-glucoside	Phenolic acids

158	1.44E+02	C10H8O	2-Naphthol*	Phenolic acids
159	1.22E+02	C7H6O2	4-Hydroxybenzaldehyde	Others
160	3.24E+02	C20H20O4	3-Prenyl-4,2',4'-Trihydroxychalcone	Flavonoids
161	1.96E+02	C6H12O7	Gluconic acid	Others
162	5.40E+02	C25H32O13	Oleuropein	Terpenoids
163	1.33E+02	C4H7NO4	Iminodiacetic acid*	Organic acids
164	3.26E+02	C15H18O8	Phenylpropionic acid-O-β-D-glucopyranoside	Phenolic acids
165	4.88E+02	C30H48O5	2,3,19-Trihydroxyurs-12-en-28-oic acid (Tormentic acid)*	Terpenoids
166	4.48E+02	C21H20O11	Kaempferol-4'-O-glucoside*	Flavonoids
167	1.27E+02	C6H6ClN	3-Chloroaniline	Alkaloids
168	1.00E+02	C4H4O3	Succinic anhydride	Organic acids
169	2.62E+02	C18H30O	Octadeca-2,9,12,15-tetraen-1-ol	Others
170	1.63E+02	C7H5N3O2	5-Nitrobenzimidazole	Alkaloids
171	1.01E+02	C6H15N	Triethylamine	Alkaloids
172	4.32E+02	C22H24O9	3,5,6,7,8,3',4'-Heptamethoxyflavone	Flavonoids
173	3.00E+02	C20H28O2	hemerocallal A	Terpenoids
174	1.01E+02	C4H7NO2	L-Azetidine-2-carboxylic acid*	Alkaloids
175	2.94E+02	C14H18N2O5	(3-(carboxyamino)-2-methylpropanoyl)phenylalanine	Amino acids and derivatives
176	2.05E+02	C9H19NO4	D-Panthenol	Others
177	3.32E+02	C13H16O10	5-O-Galloyl-D-hamamelose*	Phenolic acids
178	2.33E+02	C9H19N3O4	Ser-Lys	Amino acids and derivatives
179	2.94E+02	C18H30O3	17-Hydroxylinolenic acid	Lipids
180	2.72E+02	C17H20O3	3-hydroxy-2',5-dimethoxy-2-methylbibenzyl	Others
181	3.01E+02	C13H19NO7	1-(β-D-Glucopyranosyl)-3-ethyl-4-methyl-1H-pyrrole-2,5-dione	Others
182	2.57E+02	C11H19N3O4	Ala-Pro-Ala	Amino acids and derivatives
183	1.46E+02	C6H14N2O2	L-Lysine	Amino acids and derivatives
184	2.45E+02	C14H31NO2	Tetradecasphinganine*	Others
185	3.50E+02	C21H34O4	1-Stearidonoyl-Glycerol	Lipids
186	2.45E+02	C14H31NO2	2-Aminotetradecane-1,4-diol*	Others
187	2.79E+02	C18H33NO	Octadecadienamide	Alkaloids
188	3.00E+02	C14H20O7	Salidroside	Phenolic acids
189	1.59E+02	C6H13N3O2	δ-Guanidinovaleric acid	Organic acids
190	1.17E+02	C8H7N	Indole	Alkaloids
191	1.29E+02	C6H11NO2	Pipecolic acid	Alkaloids
192	1.94E+02	C10H10O4	Isoferulic Acid*	Phenolic acids
193	3.38E+02	C16H18O8	1-O-p-Coumaroylquinic acid*	Phenolic acids
194	3.38E+02	C16H18O8	3-(Hydroxycinnamoyl)-quinic acid*	Phenolic acids
195	2.08E+02	C11H12O4	Sinapinaldehyde	Phenolic acids

196	1.80E+02	C6H12O6	D-Galactose*	Others
197	1.01E+02	C4H7NO2	1-Aminocyclopropane-1-carboxylic acid*	Organic acids
198	2.46E+02	C10H18N2O5	L-Isoleucyl-L-Aspartate	Amino acids and derivatives
199	2.97E+02	C11H15N5O5	N7-Methylguanosine	Nucleotides and derivatives
200	2.36E+02	C12H16N2O3	Phe-Ala	Amino acids and derivatives
201	2.81E+02	C18H35NO	Oleamide (9-Octadecenamide)	Lipids
202	2.18E+02	C12H14N2O2	N-Acetyl-5-hydroxytryptamine	Alkaloids
203	2.10E+02	C6H10O8	D-Saccharic acid	Others
204	3.02E+02	C16H14O6	Dihydrokaempferide	Flavonoids
205	2.20E+02	C11H12N2O3	5-Hydroxy-L-tryptophan	Amino acids and derivatives
206	1.66E+02	C9H10O3	Ethylparaben	Phenolic acids
207	3.69E+02	C16H19NO9	Dioxindole-3-acetyl-3-O-glucoside	Alkaloids
208	3.47E+02	C10H14N5O7P	Adenosine 2'-Phosphate	Nucleotides and derivatives
209	3.23E+02	C20H37NO2	linoleoyl ethanolamine	Others
210	3.56E+02	C16H20O9	4,5,8-Trihydroxy-α-tetralone-5-O-glucoside*	Quinones
211	1.59E+02	C7H13NO3	N-acetylvaline	Amino acids and derivatives
212	3.32E+02	C13H16O10	3-O-Galloyl-D-glucose*	Phenolic acids
213	1.55E+02	C6H9N3O2	L-Histidine	Amino acids and derivatives
214	2.60E+02	C6H13O9P	D-Fructose 6-Phosphate*	Others
215	1.39E+02	C6H5NO3	2-Nitrophenol	Phenolic acids
216	1.78E+02	C10H10O3	3-Ethyl-7-hydroxyphthalide	Others
217	2.88E+02	C16H32O4	9,16-Dihydroxypalmitic acid	Lipids
218	1.37E+02	C7H9N2O	3-Carbamyl-1-methylpyridinium;(1-Methylnicotinamide)	Alkaloids
219	4.56E+02	C30H48O3	3-Hydroxyolean-12-en-28-oic acid (Oleanolic acid)*	Terpenoids
220	4.93E+02	C24H48NO7P	LysoPC 16:1*	Lipids
221	3.99E+02	C17H25N3O6S1	Tyr-Ser-Met	Amino acids and derivatives
222	1.84E+02	C6H4N2O5	2,4-Dinitrophenol	Phenolic acids
223	1.92E+02	C7H12O6	Quinic Acid	Organic acids
224	2.78E+02	C16H22O4	Diisobutyl phthalate*	Phenolic acids
225	2.03E+02	C8H17N3O3	Lys-Gly	Amino acids and derivatives
226	2.57E+02	C8H20NO6P	Choline Alfoscerate	Lipids
227	1.17E+02	C3H7N3O2	Guanidinoacetate	Alkaloids
228	2.89E+02	C16H35NO3	2-Aminohexadecane-1,5,15-triol	Others

229	3.56E+02	C16H20O9	Ferulic β-glucoside*	Phenolic acids
230	1.29E+02	C5H11N3O	4-Guanidinobutanal	Others
231	1.30E+02	C5H14N4	Agmatine	Alkaloids
232	2.29E+02	C14H31NO	Lauramine oxide	Others
233	8.34E+02	C42H42O18	Emodin(10-10')emodin double monosaccharide glucosides	Quinones
234	3.56E+02	C16H20O9	Juglanoside D	Others
235	6.76E+02	C33H56O14	Gingerglycolipid A	Lipids
236	2.94E+02	C18H30O3	3-Hydroxy-3,7,11-trimethyldodeca-1,6E,10-trien-9-yl isobutyrate*	Terpenoids
237	1.48E+02	C9H8O2	Cinnamic acid	Phenolic acids
238	4.72E+02	C30H48O4	2-Hydroxyursolic acid*	Terpenoids
239	2.72E+02	C12H16O7	Arbutin*	Phenolic acids
240	5.26E+02	C24H30O13	mudanpioside E	Others
241	1.68E+02	C8H8O4	2,5-Dimethoxybenzoquinone*	Quinones
242	1.63E+02	C6H13NO2S	L-Methionine methyl ester	Amino acids and derivatives
243	1.80E+02	C10H12O3	4-Hydroxy-3-methoxyphenylacetone	Others
244	1.80E+02	C6H12O6	D-Fructose*	Others
245	1.73E+02	C7H11NO4	Oxaceprol	Amino acids and derivatives
246	1.05E+02	C3H7NO3	L-Isoserine	Amino acids and derivatives
247	2.43E+02	C9H13N3O5	Cytidine	Nucleotides and derivatives
248	3.16E+02	C14H20O8	Grevilloside G	Phenolic acids
249	1.29E+02	C6H11NO2	Cycloleucine	Amino acids and derivatives
250	1.21E+02	C7H7NO	Benzamide	Alkaloids
251	2.78E+02	C17H26O3	Fusarester B	Others
252	3.86E+02	C17H22O10	1-O-Sinapoyl-β-D-glucose	Phenolic acids
253	5.21E+02	C26H52NO7P	LysoPC 18:1*	Lipids
254	1.94E+02	C6H10O7	D-Glucoronic acid*	Others
255	4.93E+02	C24H48NO7P	LysoPC 16:1(2n isomer)*	Lipids
256	1.62E+02	C10H10O2	2-Methyl-3-hydroxyindan-1-one	Others
257	3.76E+02	C16H24O10	8-Epiloganic acid	Terpenoids
258	4.75E+02	C23H42NO7P	LysoPE 18:3	Lipids
259	1.32E+02	C5H8O4	4-Hydroxy-2-Oxopentanoic Acid*	Organic acids
260	2.24E+02	C13H20O3	Blumenol A	Terpenoids
261	4.88E+02	C30H48O5	2,3,23-Trihydroxyolean-12-en-28-oic acid*	Terpenoids
262	2.21E+02	C8H15NO6	N-Acetyl-D-galactosamine	Others
263	1.61E+02	C6H11NO4	L-2-Aminoadipate*	Amino acids and derivatives
264	1.74E+02	C6H6O6	trans-Aconitic acid	Organic acids

265	5.24E+02	C26H36O11	Secoisolariciresinol 4-O-glucoside	Lignans and Coumarins
266	1.86E+02	C3H7O7P	3-Phospho-D-glyceric acid	Others
267	3.74E+02	C20H22O7	Nortrachelogenin	Lignans and Coumarins
268	2.76E+02	C18H28O2	Macrophypene B	Terpenoids
269	5.77E+02	C28H52NO9P	1-(2,3-dihydroxypropoxy)-3-(((2-(dimethylamino)ethoxy)(hydroxy)phosphoryl)oxy)propan-2-yl (8E,11Z,14Z)-octadeca-8,11,14-trienoate	Others
270	1.32E+02	C5H8O4	2-Methylsuccinic acid*	Organic acids
271	1.32E+02	C5H8O4	Monomethyl succinate*	Organic acids
272	5.30E+02	C26H26O12	4,5-O-Dicaffeoylquinic Acid Methyl Ester	Phenolic acids
273	3.32E+02	C14H20O9	2,6-Dimethoxypydroquinone-1-O-glucoside	Flavonoids
274	1.88E+02	C7H16N4O2	N-Monomethyl-L-arginine*	Amino acids and derivatives
275	5.21E+02	C26H52NO7P	1-Vaccenoyl-Glycero-3-Phosphocholine*	Others
276	2.08E+02	C11H12O4	Ferulic acid methyl ester	Phenolic acids
277	5.34E+02	C27H34O11	Arctiin	Lignans and Coumarins
278	2.34E+02	C15H22O2	Curcumenol	Terpenoids
279	2.96E+02	C18H32O3	9-Oxo-12Z-Octadecenoic acid*	Lipids
280	2.96E+02	C18H32O3	9S-Hydroxy-10E,12Z-octadecadienoic acid*	Lipids
281	2.51E+02	C10H13N5O3	Cordycepin (3'-Deoxyadenosine)*	Nucleotides and derivatives
282	3.12E+02	C11H20O10	4-O-galactopyranosylxylose	Others
283	2.61E+02	C10H19N3O5	Lys-Asp*	Amino acids and derivatives
284	1.73E+02	C7H15N3O2	N5-(1-Iminoethyl)-L-ornithine	Amino acids and derivatives
285	3.38E+02	C20H34O4	7β-Hydroxydarutigenol	Terpenoids
286	1.46E+02	C6H10O4	2,2-Dimethylsuccinic acid	Organic acids
287	1.90E+02	C11H10O3	2,5-Dimethyl-7-hydroxychromone	Others
288	5.19E+02	C26H50NO7P	LysoPC 18:2	Lipids
289	2.40E+02	C15H16N2O	Arenarine B	Alkaloids
290	1.48E+02	C5H8O5	3-Hydroxyglutaric acid*	Organic acids
291	6.60E+02	C6H18O24P6	Phytic acid	Organic acids
292	1.03E+02	C4H9NO2	Methyl 3-aminopropanoate	Amino acids and derivatives
293	2.94E+02	C17H26O4	Embelin	Quinones
294	2.08E+02	C10H8O5	Isofraxetin	Lignans and Coumarins
295	3.10E+02	C20H38O2	Eicosenoic acid	Lipids
296	2.78E+02	C18H30O2	6,10,14-Trimethylpentadeca-5,9-Diene-2,13-Dione	Others

297	1.90E+02	C12H14O2	Butylphthalide	Others
298	5.18E+02	C26H30O11	Balanophonin glucoside	Lignans and Coumarins
299	6.10E+02	C27H30O16	Quercetin-3-O-robinobioside	Flavonoids
300	3.56E+02	C16H20O9	Linocaffein	Phenolic acids
301	1.88E+02	C7H16N4O2	Arginine methyl ester*	Amino acids and derivatives
302	2.92E+02	C18H28O3	9s,13r-12-Oxophytodienoic Acid	Lipids
303	5.36E+02	C26H32O12	Caudatoside A	Terpenoids
304	5.04E+02	C30H48O6	2,3,19,23-Tetrahydroxyurs-12-en-28-oic acid	Terpenoids
305	5.33E+02	C27H52NO7P	LysoPC 19:2(2n isomer)	Lipids
306	2.52E+02	C12H16N2O4	Phe-Ser	Amino acids and derivatives
307	2.18E+02	C15H22O	α-Cyperone	Others
308	6.96E+02	C32H40O17	6''-O-p-Coumaroylgenipin gentiobioside	Terpenoids
309	2.40E+02	C15H12O3	Chrysophanol-9-anthrone	Quinones
310	5.10E+02	C28H30O9	2,7-Dihydroxy-1-(p-hydroxybenzyl)-4-methoxy-9,10-dihydrophenanthrene-4'-O-glucose	Others
311	3.84E+02	C22H28N2O4	Isorhynchophylline*	Alkaloids
312	4.82E+02	C25H22O10	Silicristin	Flavonoids
313	2.86E+02	C16H30O4	Hexadecanedioic acid	Lipids
314	3.32E+02	C13H16O10	2-O-Galloyl-D-glucose	Phenolic acids
315	2.80E+02	C15H22NO4+	Feruloylcholine	Alkaloids
316	3.16E+02	C13H16O9	1-O-Gentisoyl-β-D-glucoside*	Phenolic acids
317	2.62E+02	C11H22N2O3S	Met-Leu	Amino acids and derivatives
318	6.11E+02	C27H31O16+	Cyanidin-3-O-(2''-O-glucosyl)glucoside	Flavonoids
319	6.50E+02	C31H38O15	xylosyl phellodendroside	Flavonoids
320	1.66E+02	C10H14O2	Rhododendrol	Phenolic acids
321	1.66E+02	C10H14O2	4-Isopropenylcyclohexene-1-carboxylic acid	Organic acids
322	1.66E+02	C10H14O2	Actinidialactone	Terpenoids
323	1.88E+02	C7H16N4O2	Homoarginine	Amino acids and derivatives
324	2.76E+02	C17H24O3	Lycoposerramine E	Alkaloids
325	2.56E+02	C16H32O2	Cetostearic acid	Lipids
326	4.88E+02	C30H48O5	2,3,6-Trihydroxyurs-12-en-28-oic acid (Madasiatic acid)*	Terpenoids
327	2.34E+02	C15H22O2	Ligudicin D	Terpenoids
328	5.49E+02	C28H56NO7P	LysoPC 20:1	Lipids
329	2.73E+02	C16H35NO2	Lauryldiethanolamine*	Others
330	2.20E+02	C15H24O	2-trans,6-trans-Farnesal	Others
331	2.48E+02	C15H20O3	Micheliolide	Terpenoids
332	5.19E+02	C26H50NO7P	LysoPC 18:2(2n isomer)	Lipids
333	3.82E+02	C20H30O7	3,4,5-trihydroxy-6-(4-octylphenoxy)oxane-2-ca	Phenolic acids

			rboxylic acid	
334	1.52E+02	C8H8O3	Isovanillin	Others
335	2.02E+02	C9H18N2O3	L-Alanyl-L-leucine	Amino acids and derivatives
336	2.24E+02	C11H12O5	Sinapic acid	Phenolic acids
337	3.00E+02	C13H16O8	1-O-Salicyloyl-β-D-glucose*	Phenolic acids
338	1.66E+02	C6H14O5	L-Fucitol	Others
339	4.93E+02	C23H25O12+	Malvidin-3-O-glucoside	Flavonoids
340	1.96E+02	C10H12O4	3-Hydroxy-1-(4'-hydroxy-3'-methoxyphenyl)-propan-1-one	Others
341	3.61E+02	C20H43NO4	2-AminoicoSane-1,5,7,19-tetraol	Others
342	1.33E+02	C4H7NO4	L-Aspartic Acid*	Amino acids and derivatives
343	2.06E+02	C11H10O4	5,7-Dimethoxycoumarin (Limettin)(Citropten)	Lignans and Coumarins
344	2.45E+02	C10H19N3O4	Asn-Ile	Amino acids and derivatives
345	1.65E+02	C9H11NO2	L-Phenylalanine	Amino acids and derivatives
346	2.94E+02	C18H30O3	2R-hydroxy-9Z,12Z,15Z-octadecatrienoic acid	Lipids
347	2.18E+02	C15H22O	Santalal	Terpenoids
348	3.78E+02	C22H34O5	Previtexilactone	Terpenoids
349	4.64E+02	C22H24O11	Hesperetin-5-O-glucoside	Flavonoids
350	4.48E+02	C21H36O10	Geranyl 3-O-xylopyranosyl-glucopyranoside*	Others
351	8.16E+02	C36H48O21	kurroaoside B	Others
352	4.50E+02	C21H22O11	Eriodictyol-7-O-glucoside	Flavonoids
353	2.80E+02	C19H20O2	1-phenyl-7-(4-hydroxyphenyl)-4-ene-3-heptanone	Others
354	1.17E+02	C5H11NO2	alanine betaine*	Alkaloids
355	4.48E+02	C21H36O10	Borneol 7-O-[β-D-apiofuranosyl-(1→6)]-β-D-glucopyranoside*	Terpenoids
356	5.04E+02	C30H48O6	2,3,19,23-Tetrahydroxyolean-12-en-28-oic acid	Terpenoids
357	1.46E+02	C6H10O4	Mono-Methyl Glutarate*	Organic acids
358	3.56E+02	C16H20O9	Juglanoside E	Others
359	5.95E+02	C27H31O15+	Cyanidin-3-O-rutinoside (Keracyanin)	Flavonoids
360	1.38E+02	C9H14O	Isophorone	Others
361	3.26E+02	C15H18O8	6-O-p-Coumaroyl-β-D-glucose	Phenolic acids
362	1.94E+02	C10H10O4	Dimethyl phthalate	Phenolic acids
363	1.48E+02	C5H8O5	L-Citramalic acid	Organic acids
364	2.56E+02	C16H32O2	Palmitic acid	Lipids
365	1.58E+02	C10H22O	1-Decanol*	Others
366	1.48E+02	C5H8O5	2-Hydroxyglutaric Acid*	Organic acids
367	3.56E+02	C16H20O9	Veranisatin B	Terpenoids

368	2.96E+02	C18H32O3	15(R)-Hydroxylinoleic Acid*	Lipids
369	1.80E+02	C6H12O6	D-Glucose*	Others
370	2.61E+02	C10H19N3O5	Asp-Lys*	Amino acids and derivatives
371	1.50E+02	C5H10O5	D-Ribose	Others
372	5.23E+02	C26H54NO7P	LysoPC 18:0(2n isomer)	Lipids
373	1.58E+02	C10H22O	2-Decanol*	Others
374	1.69E+02	C12H11N	Diphenylamine	Alkaloids
375	1.31E+02	C6H13NO2	L-Isoleucine*	Amino acids and derivatives
376	3.89E+02	C14H23N5O8	Asp-Gln-Gln	Amino acids and derivatives
377	1.09E+02	C6H7NO	2-Aminophenol	Alkaloids
378	1.20E+02	C4H8O4	D-Threose	Others
379	3.88E+02	C15H28N6O6	Asn-Lys-Gln	Amino acids and derivatives
380	3.35E+02	C15H21N5O4	Riboprine	Nucleotides and derivatives
381	3.48E+02	C16H28O8	Kankanoside E	Terpenoids
382	2.87E+02	C16H17NO4	Tetrahydropapaveroline	Alkaloids
383	1.51E+02	C8H9NO2	4-methoxybenzamide	Alkaloids
384	1.80E+02	C6H12O6	D-Mannose*	Others
385	4.79E+02	C23H46NO7P	LysoPC 15:1	Lipids
386	6.58E+02	C33H38O14	Xyloccensin U	Terpenoids
387	1.35E+02	C4H9NO2S	L-Homocysteine	Amino acids and derivatives
388	1.46E+02	C6H10O4	2-Methylglutaric acid*	Organic acids
389	1.46E+02	C6H10O4	Adipic Acid*	Organic acids
390	2.56E+02	C15H12O4	Pinocembrin (Dihydrochrysin)	Flavonoids
391	1.48E+02	C5H8O5	2-Dehydro-3-deoxy-L-arabinonate*	Others
392	7.12E+02	C33H44O17	Medioresinol-4,4'-di-O-glucoside	Lignans and Coumarins
393	3.32E+02	C10H13N4O7P	2'-Deoxyinosine-5'-monophosphate	Nucleotides and derivatives
394	4.72E+02	C30H48O4	Rubianol-f	Terpenoids
395	1.47E+02	C8H5NO2	Isatin	Alkaloids
396	2.22E+02	C11H10O5	Umckalin (7-hydroxy-5,6-dimethoxycoumarin)	Lignans and Coumarins
397	3.46E+02	C15H22O9	6-DeoxyCatalpol	Terpenoids
398	4.98E+02	C26H42O9	Suavioside F	Terpenoids
399	3.72E+02	C16H20O10	Hydroxyferulic acid glucoside	Phenolic acids
400	2.82E+02	C18H34O2	Petroselinic acid*	Lipids
401	1.76E+02	C7H12O5	2-Isopropylmalic Acid	Organic acids
402	2.04E+02	C11H12N2O2	L-Tryptophan	Amino acids and

				derivatives
403	1.31E+02	C6H13NO2	DL-Leucine*	Amino acids and derivatives
404	1.31E+02	C6H13NO2	L-Norleucine*	Amino acids and derivatives
405	3.76E+02	C16H24O10	Cornusoside A	Others
406	2.71E+02	C12H21N3O4	Pro-Gly-Val	Amino acids and derivatives
407	2.94E+02	C18H30O3	9-Oxo-10,12-Octadecadienoic Acid*	Lipids
408	3.86E+02	C19H30O8	Sonnerstigmane D	Terpenoids
409	2.99E+02	C18H37NO2	3-Dehydrosphinganine	Lipids
410	6.60E+02	C31H32O16	1,5-O-dicaffeoyl-3-O-dimethylmalyl-quinic acid	Phenolic acids
411	5.00E+02	C25H24O11	1,4,8-Trihydroxynaphthalene-1-O-[6'-O-(3'',4'', 5''-trimethylbenzoyl)]glucoside	Quinones
412	1.92E+02	C13H20O	α-Ionone	Terpenoids
413	3.31E+02	C10H14N5O6P	2'-Deoxyadenosine-5'-monophosphate	Nucleotides and derivatives
414	1.62E+02	C9H6O3	7-Hydroxycoumarin;Umbelliferone	Lignans and Coumarins
415	2.20E+02	C11H12N2O3	(3S)-1,3-dihydroxy-2,3,3a,4-tetrahydropyrrolo[2,1-b]quinazolin-9(1H)-one	Alkaloids
416	3.64E+02	C16H28O9	Glucosyl 6,9-dihydroxydec-4-enoic acid	Others
417	1.42E+02	C8H14O2	cis-3-Hexenyl acetate*	Lipids
418	1.42E+02	C6H6O4	Kojic acid	Organic acids
419	3.30E+02	C18H34O5	9,12,13-TriHOME; 9(S),12(S),13(S)-Trihydroxy-10(E)-octadecenoic acid	Lipids
420	5.35E+02	C27H54NO7P	LysoPC 19:1	Lipids
421	4.50E+02	C21H22O11	Eriodictyol-3'-O-glucoside	Flavonoids
422	2.03E+02	C7H13N3O4	Gln-Gly	Amino acids and derivatives
423	5.16E+02	C25H24O12	Isochlorogenic acid B	Phenolic acids
424	5.34E+02	C26H30O12	Graminone A glucoside	Lignans and Coumarins
425	6.54E+02	C31H58O14	1-Palmitoyl-Sn-Glycerol 3-O-Diglucoside	Lipids
426	3.60E+02	C15H20O10	Syringoyl-D-Glucose	Phenolic acids
427	3.50E+02	C15H26O9	Eucommioside	Terpenoids
428	1.92E+02	C11H16N2O	N-(4-Aminobutyl)benzamide	Alkaloids
429	1.43E+02	C7H13NO2	1-Methylpiperidine-2-carboxylic acid	Organic acids
430	1.81E+02	C9H11NO3	3-Hydroxy-L-phenylalanine*	Amino acids and derivatives
431	1.31E+02	C6H13NO2	propyl-L-alanine	Amino acids and derivatives
432	1.31E+02	C6H13NO2	D-Allo-Isoleucine*	Amino acids and

				derivatives
433	2.42E+02	C15H30O2	Pentadecanoic Acid	Lipids
434	2.22E+02	C13H18O3	Dehydrovomifoliol	Terpenoids
435	4.88E+02	C30H48O5	2α,3α-Dihydroxyursolic acid*	Terpenoids
436	5.79E+02	C28H54NO9P	1-(2,3-dihydroxypropoxy)-3-(((2-(dimethylamino)ethoxy)(hydroxy)phosphoryl)oxy)propan-2-yl (11Z,14Z)-octadeca-11,14-dienoate	Others
437	4.53E+02	C21H44NO7P	LysoPE 16:0	Lipids
438	2.19E+02	C9H17NO5	D-Pantothenic Acid*	Others
439	2.44E+02	C9H12N2O6	1-Arabinosyluracil	Nucleotides and derivatives
440	1.32E+02	C5H8O4	Glutaric acid*	Organic acids
441	1.17E+02	C5H11NO2	Betaine	Alkaloids
442	3.52E+02	C21H36O4	1-Monolinolenoyl-Rac-Glycerol*	Lipids
443	3.17E+02	C18H39NO3	4-Hydroxysphinganine; Phytosphingosine	Lipids
444	5.80E+02	C15H22N2O18P2	Uridine-5'-diphosphoglucuronic acid	Nucleotides and derivatives
445	5.20E+02	C25H28O12	6'-Trans-Cinnamoyl-8-epikingisidic acid	Phenolic acids
446	2.38E+02	C11H14N2O4	Gly-Tyr*	Amino acids and derivatives
447	2.30E+02	C9H14N2O5	Pro-Asp	Amino acids and derivatives
448	4.32E+02	C19H28O11	Benzyl-(2''-O-glucosyl)glucoside	Phenolic acids
449	4.26E+02	C30H50O	9,19-Cyclolanost-24-en-3-ol (Cycloartenol)	Terpenoids
450	4.72E+02	C30H48O4	nahagenin	Terpenoids
451	5.48E+02	C23H32O15	6'-O-Sinapoylsucrose	Phenolic acids
452	4.72E+02	C30H48O4	2,3-Dihydroxyurs-12-en-28-oic acid (Corosolic acid)*	Terpenoids
453	1.66E+02	C5H10O6	D-Xylonic acid	Others
454	2.02E+02	C14H18O	2-Pentyl-3-phenyl-2-propenal	Others
455	1.19E+02	C4H9NO3	DL-Threonine	Amino acids and derivatives
456	3.20E+02	C16H16O7	3-O-p-Coumaroylshikimic acid	Phenolic acids
457	3.00E+02	C18H36O3	2R-Hydroxyoctadecanoic Acid*	Lipids
458	5.51E+02	C24H23O15+	Delphinidin-3-O-(6''-O-malonyl)glucoside	Flavonoids
459	2.51E+02	C10H13N5O3	5'-Deoxyadenosine*	Nucleotides and derivatives
460	1.61E+02	C6H11NO4	N-Acetyl-L-threonine	Amino acids and derivatives
461	1.98E+02	C11H18O3	5-hydroxy-3,4-dimethyl-5-pentylfuran-2(5H)-one	Others
462	2.02E+02	C8H18N4O2	3-(triazan-2-yl)propyl L-prolinate	Amino acids and derivatives
463	3.20E+02	C20H32O3	14-Hydroxyvibsanin F	Terpenoids

464	5.36E+02	C14H22N2O16P2	Uridine-5'-Diphosphate-D-Xylose	Nucleotides and derivatives
465	1.01E+02	C4H7NO2	Azetidine-2-carboxylic acid*	Alkaloids
466	1.13E+02	C5H7NO2	3,4-Dehydro-DL-proline	Amino acids and derivatives
467	1.68E+02	C8H8O4	5-Methoxysalicylic acid	Phenolic acids
468	3.55E+02	C14H21N5O6	His-Ala-Glu	Amino acids and derivatives
469	2.98E+02	C20H42O	1-Eicosanol	Lipids
470	5.36E+02	C26H32O12	Scrophuloside A1	Terpenoids
471	1.75E+02	C6H9NO5	N-Acetyl-L-Aspartic Acid	Amino acids and derivatives
472	4.78E+02	C21H18O13	Quercetin-4'-O-glucuronide	Flavonoids
473	5.04E+02	C30H48O6	2,3,6,23-Tetrahydroxyolean-12-en-28-oic acid (Protobassic acid)	Terpenoids
474	2.96E+02	C18H32O3	13(S)-HODE;13(S)-Hydroxyoctadeca-9Z,11E-dienoic acid*	Lipids
475	2.60E+02	C11H20N2O5	L-γ-Glutamyl-L-leucine	Amino acids and derivatives
476	5.52E+02	C27H36O12	5'-Methoxyisolariciresinol-9'-O-glucoside	Lignans and Coumarins
477	6.66E+02	C24H42O21	D-Maltotetraose	Others
478	6.50E+02	C33H30O14	1,5-O-dicaffeoyl-3-O-p-coumarin-glucose	Phenolic acids
479	4.39E+02	C20H42NO7P	LysoPE 15:0(2n isomer)	Lipids
480	1.36E+02	C5H4N4O	Hypoxanthine	Nucleotides and derivatives
481	1.36E+02	C8H8O2	Phenyl acetate	Phenolic acids
482	1.72E+02	C7H8O5	3-Dehydroshikimic acid	Organic acids
483	3.48E+02	C10H13N4O8P	Inosine 5'-monophosphate	Nucleotides and derivatives
484	3.64E+02	C20H12O7	Gallein	Phenolic acids
485	3.02E+02	C13H18O8	4-O-Glucosyl-3,4-dihydroxybenzyl alcohol	Phenolic acids
486	1.78E+02	C10H10O3	Coniferaldehyde*	Others
487	1.47E+02	C6H13NO3	4-Hydroxy-L-Isoleucine	Amino acids and derivatives
488	1.38E+02	C8H10O2	2-Methoxy-4-methylphenol	Phenolic acids
489	3.42E+02	C12H22O11	Galactinol	Others
490	4.88E+02	C30H48O5	Rubianol-a	Terpenoids
491	3.02E+02	C13H18O8	Idesin	Phenolic acids
492	1.45E+02	C6H11NO3	Allysine(6-Oxo DL-Norleucine)	Amino acids and derivatives
493	2.02E+02	C8H18N4O2	NG,NG-Dimethyl-L-arginine*	Amino acids and derivatives
494	1.92E+02	C6H8O7	Citric Acid	Organic acids

495	2.06E+02	C7H10O7	Homocitrate	Organic acids
496	3.25E+02	C20H39NO2	N-Oleoylethanolamine	Alkaloids
497	3.70E+02	C19H30O7	(6S,9R)-Deoxysappanol	Phenolic acids
498	1.65E+02	C6H7N5O	7-Methylguanine	Nucleotides and derivatives
499	1.93E+02	C10H11NO3	3,3-dimethoxyindolequinone	Alkaloids
500	1.32E+02	C5H12N2O2	L-Ornithine	Amino acids and derivatives
501	1.50E+02	C9H10O2	M-Tolyl acetate	Others
502	1.80E+02	C10H12O3	Propyl 4-hydroxybenzoate	Phenolic acids
503	3.88E+02	C18H28O9	5'-Glucosyloxyjasmanic acid	Phenolic acids
504	1.46E+02	C5H10N2O3	L-Glutamine	Amino acids and derivatives
505	5.16E+02	C25H24O12	Isochlorogenic acid A*	Phenolic acids
506	2.94E+02	C17H26O4	[6]-Gingerol	Phenolic acids
507	1.42E+02	C8H14O2	2-n-Propyl-4-pentenoic acid	Organic acids
508	1.17E+02	C8H7N	m-Aminophenylacetylene	Alkaloids
509	2.82E+02	C13H18N2O5	Thr-Tyr	Amino acids and derivatives
510	3.00E+02	C20H28O2	Dehydroabietic acid	Terpenoids
511	1.78E+02	C10H10O3	3,4-Methylenedioxy cinnamyl alcohol*	Others
512	2.44E+02	C13H24O4	Tridecanedioic acid	Lipids
513	2.38E+02	C12H14O5	1-O-p-Cumaroylglycerol	Phenolic acids
514	3.16E+02	C13H16O9	Protocatechuic acid-4-O-glucoside*	Phenolic acids
515	2.86E+02	C13H18O7	Helicidol	Phenolic acids
516	2.48E+02	C15H20O3	Codonolactone	Terpenoids
517	3.42E+02	C12H22O11	Melibiose	Others
518	2.81E+02	C18H35NO	Octadec-2-enamide	Alkaloids
519	2.85E+02	C12H15NO7	N-(beta-D-Glucosyl)nicotinate	Others
520	2.14E+02	C5H11O7P	2-Deoxyribose-1-phosphate	Nucleotides and derivatives
521	5.34E+02	C27H34O11	2,,7-di-O-β-D-glucose-4-methoxy-9,10-dihydro phenanthrene	Quinones
522	1.38E+02	C6H6N2O2	Urocanic acid	Organic acids
523	2.75E+02	C16H21NO3	Methylenedioxypyrovalerone	Others
524	1.52E+02	C10H16O	cis-Citral	Terpenoids
525	3.29E+02	C10H12N5O6P	Cyclic 3',5'-Adenylic acid	Nucleotides and derivatives
526	1.31E+02	C4H9N3O2	3-Guanidinopropionic acid	Organic acids
527	1.42E+02	C6H6O4	Muconic acid	Organic acids
528	1.45E+02	C5H11N3O2	4-Guanidinobutyric acid	Organic acids
529	1.74E+02	C8H14O4	2-Propylglutaric acid	Organic acids
530	4.06E+02	C18H22N4O7	Trp-Ser-Asp	Amino acids and derivatives

531	5.32E+02	$C_{26}H_{28}O_{12}$	Sesaminol 2-O-Beta-D-Glucoside	Lignans and Coumarins
532	3.78E+02	$C_{22}H_{34}O_5$	Negundoin A	Terpenoids
533	1.88E+02	$C_8H_{16}N_2O_3$	N6-Acetyl-L-lysine	Amino acids and derivatives
534	2.07E+02	$C_{11}H_{13}NO_3$	N-(acetyl)phenylalanine	Amino acids and derivatives
535	1.61E+02	$C_9H_7NO_2$	Indole-3-carboxylic acid*	Alkaloids
536	2.67E+02	$C_{10}H_{13}N_5O_4$	2'-Deoxyguanosine	Nucleotides and derivatives
537	3.33E+02	$C_{16}H_{19}N_3O_5$	Tryptophan glutamic acid	Amino acids and derivatives
538	2.05E+02	$C_{10}H_7NO_4$	4-hydroxy-2-oxo-1,2-dihydroquinoline-3-carboxylic acid	Alkaloids
539	4.52E+02	$C_{30}H_{44}O_3$	3-Hydroxyurs-5(6),12,18(19)-trien-28-oic acid (Uncargenin B)	Terpenoids
540	1.64E+02	$C_6H_{12}O_5$	1,5-Anhydro-D-glucitol	Others
541	2.67E+02	$C_{12}H_{13}NO_6$	N-(3,4,5-trihydroxycinnamicacid)-alanine	Amino acids and derivatives
542	4.50E+02	$C_{21}H_{22}O_{11}$	Maesopsin 4-O-Glucoside(Hovetrichoside C)	Flavonoids
543	3.84E+02	$C_{17}H_{20}O_{10}$	Isofraxidin-7-O-glucoside	Lignans and Coumarins
544	3.12E+02	$C_{18}H_{32}O_4$	9S-Hydroperoxy-10E,12Z-octadecadienoic acid	Lipids
545	1.65E+02	$C_5H_{11}NO_3S$	L-Methionine Sulfoxide	Amino acids and derivatives
546	1.50E+02	$C_5H_{10}O_5$	DL-Xylose*	Others
547	3.30E+02	$C_{14}H_{18}O_9$	Vanillic acid-4-O-glucoside	Phenolic acids
548	4.46E+02	$C_{21}H_{34}O_{10}$	Dihydropenstemide	Terpenoids
549	1.89E+02	$C_8H_{15}NO_4$	(2-carboxyethyl)-L-valine	Amino acids and derivatives
550	1.29E+02	$C_5H_7NO_3$	5-Oxoproline*	Amino acids and derivatives
551	4.78E+02	$C_{22}H_{22}O_{12}$	Rhamnetin-3-O-Glucoside*	Flavonoids
552	3.02E+02	$C_{20}H_{30}O_2$	Abietic acid	Terpenoids
553	4.95E+02	$C_{24}H_{50}NO_7P$	LysoPC 16:0	Lipids
554	4.70E+02	$C_{22}H_{26}N_6O_6$	Trp-Glu-His	Amino acids and derivatives
555	3.72E+02	$C_{21}H_{24}O_6$	Arctigenin	Lignans and Coumarins
556	3.56E+02	$C_{16}H_{20}O_9$	6-O-Feruloyl-β-D-glucose	Phenolic acids
557	2.26E+02	$C_{10}H_{14}N_2O_4$	Cyclo(Pro-Glu)	Amino acids and derivatives
558	1.15E+02	$C_5H_9NO_2$	L-Proline*	Amino acids and derivatives

559	4.70E+02	C30H46O4	2,3-Dihydroxyoleana-11,13(18)-dien-28-oic acid (Camaldulenic acid)	Terpenoids
560	1.78E+02	C10H10O3	3-Hydroxy-3,7-dimethyl-2-benzofuran-1(3H)-one	Others
561	1.35E+02	C5H5N5	Zarzissine	Alkaloids
562	1.50E+02	C5H10O5	L-Xylose*	Others
563	1.43E+02	C7H13NO2	L-Cyclopentylglycine	Amino acids and derivatives
564	3.52E+02	C21H36O4	(S)-2,3-dihydroxypropyl (9Z,12Z,15Z)-octadeca-9,12,15-trienoate	Others
565	5.66E+02	C28H38O12	6'-O-Sinapoyljasminoside B	Terpenoids
566	1.98E+02	C11H18O3	Robinlin	Terpenoids
567	1.76E+02	C10H8O3	3,4-Methylenedioxycinnamaldehyde	Others
568	8.00E+02	C38H40O19	Isovitexin-7-O-(6''-sinapoyl)glucoside	Flavonoids
569	2.90E+02	C7H15O10P	D-Sedoheptuiose 7-phosphate	Others
570	3.34E+02	C11H15N2O8P	β-Nicotinamide mononucleotide	Nucleotides and derivatives
571	6.12E+02	C20H32N6O12S2	Oxiglutatione	Amino acids and derivatives
572	3.42E+02	C15H18O9	Grevilloside F	Phenolic acids
573	3.12E+02	C11H20O10	6-O-alpha-L-arabinopyranosyl-D-glucopyranose	Others
574	2.02E+02	C10H26N4	Spermine	Alkaloids
575	4.88E+02	C30H48O5	Cannabifolin C	Terpenoids
576	4.88E+02	C30H48O5	Cordianol B	Terpenoids
577	6.10E+02	C27H30O16	Quercetin-3-O-rutinoside (Rutin)	Flavonoids
578	5.24E+02	C26H36O11	Javanicolide C	Terpenoids
579	4.48E+02	C19H28O12	6-Epi-barlerin	Terpenoids
580	3.18E+02	C20H30O3	12-Hydroxyabietic Acid	Terpenoids
581	5.92E+02	C28H32O14	Disinapoyl glucoside	Phenolic acids
582	4.81E+02	C23H48NO7P	LysoPC 15:0(2n isomer)*	Lipids
583	1.82E+02	C9H10O4	Syringaldehyde; 4-Hydroxy-3,5-Dimethoxybenzaldehyde	Others
584	3.32E+02	C14H20O9	2-(3,4-dihydroxyphenyl)ethanediol 1-O-β-D-glucopyranoside*	Phenolic acids
585	3.32E+02	C14H20O9	Koaburaside*	Phenolic acids
586	3.32E+02	C14H20O9	Leonuriside A*	Phenolic acids
587	5.04E+02	C30H48O6	3β,6β,19α,24-Tetrahydroxyurs-12-en-28-oic acid	Terpenoids
588	5.47E+02	C28H54NO7P	LysoPC 20:2(2n isomer)*	Lipids
589	1.88E+02	C8H16N2O3	N-Glycyl-L-leucine*	Amino acids and derivatives
590	4.18E+02	C22H26O8	3,3'-Bis(3,4-dihydro-4-hydroxy-6,8-dimethoxy-2H-1-benzopyran)	Others
591	4.82E+02	C24H26N4O7	Tyr-Trp-Asp	Amino acids and

				derivatives
592	3.88E+02	C17H24O10	7-Dehydroxyzaluzioside	Terpenoids
593	4.32E+02	C18H24O12	4-Hydroxybenzoic acid glucosyl xyloside*	Phenolic acids
594	4.64E+02	C21H20O12	Quercetin-4'-O-glucoside (Spiraeoside)	Flavonoids
595	4.70E+02	C30H46O4	Rubiarbonone E*	Terpenoids
596	1.31E+02	C6H13NO2	L-Leucine*	Amino acids and derivatives
597	2.58E+02	C14H26O4	1,14-Tetradecanedioic Acid	Lipids
598	4.32E+02	C18H24O12	3,4-dihydroxybenzaldehyde-xylose-glucoside*	Phenolic acids
599	3.24E+02	C13H24O9	3-(Beta-D-Glucopyranosyloxy)-5-Hydroxyhexanoic Acid Methyl Ester	Organic acids
600	1.94E+02	C10H10O4	4,5,8-Trihydroxy-α-tetralone	Quinones
601	1.76E+02	C6H8O6	L-Ascorbic acid (Vitamin C)	Others
602	1.65E+02	C8H7NO3	2-Hydroxy-1,4-benzoxazine-3(2H)-one (HBOA)	Alkaloids
603	1.03E+02	C4H9NO2	N,N-Dimethylglycine*	Amino acids and derivatives
604	2.10E+02	C6H10O8	D-Galactaric acid	Others
605	2.62E+02	C6H15O9P	Sorbitol-6-phosphate	Others
606	3.46E+02	C16H26O8	Glucosyl 6-Hydroxy-2,6-Dimethyl-2E,7-Octadienoate	Others
607	2.86E+02	C20H30O	Retinol (Vitamin A1)	Others
608	1.11E+02	C5H9N3	Histamine	Alkaloids
609	1.65E+02	C6H7N5O	6-O-methylguanine	Nucleotides and derivatives
610	5.16E+02	C25H24O12	Isochlorogenic acid C*	Phenolic acids
611	1.64E+02	C9H8O3	Phenylpyruvic acid	Organic acids
612	3.39E+02	C20H21NO4	Canadine; Tetrahydroberberine	Alkaloids
613	2.07E+02	C11H13NO3	N-Acetyl-DL-phenylalanine	Amino acids and derivatives
614	2.94E+02	C19H34O2	E,E,Z-1,3,12-Nonadecatriene-5,14-diol	Lipids
615	2.51E+02	C10H13N5O3	2'-Deoxyadenosine*	Nucleotides and derivatives
616	2.36E+02	C12H12O5	Dimethylfraxetin; 6,7,8-Trimethoxycoumarin*	Lignans and Coumarins
617	2.34E+02	C12H14N2O3	Cyclo(Tyr-Ala)	Amino acids and derivatives
618	2.82E+02	C18H34O2	Oleic acid	Lipids
619	3.42E+02	C12H22O11	D-Sucrose*	Others
620	3.36E+02	C21H36O3	Vitetrifolin H	Terpenoids
621	2.77E+02	C10H19N3O6	Ala-Ser-Thr	Amino acids and derivatives
622	6.82E+02	C36H58O12	2,3,19,23,24-Pentahydroxyolean-12-en-28-oic acid-28-O-glucoside	Terpenoids
623	2.18E+02	C12H10O4	Baldrinal	Terpenoids

624	3.32E+02	C13H16O10	6-O-Galloyl-β-D-glucose*	Phenolic acids
625	1.89E+02	C7H11NO5	(2S)-2-Amino-6-oxoheptanedioic acid	Alkaloids
626	3.30E+02	C18H34O5	Tianshic acid	Organic acids
627	3.10E+02	C14H18N2O6	2-((L-tyrosyl)oxy)-5-amino-5-oxopentanoic acid	Amino acids and derivatives
628	9.50E+01	C5H5NO	3-Hydroxypyridine	Alkaloids
629	2.77E+02	C9H15N3O7	Gly-Asp-Ser	Amino acids and derivatives
630	7.02E+02	C36H46O14	Trichilin H	Terpenoids
631	1.54E+02	C10H18O	Eucalyptol; 1,8-Cineole	Terpenoids
632	3.46E+02	C16H26O8	Rehmapicroside	Terpenoids
633	3.54E+02	C16H18O9	Cryptochlorogenic acid (4-O-Caffeoylquinic acid)*	Phenolic acids
634	2.61E+02	C14H31NO3	2-Aminotetradecane-1,5,13-triol	Others
635	3.30E+02	C14H18O9	5-Glucosyloxy-2-Hydroxybenzoic acid methyl ester	Phenolic acids
636	3.26E+02	C12H22O10	Rutinose	Others
637	4.02E+02	C21H22O8	Nobiletin (5,6,7,8,3',4'-Hexamethoxyflavone)	Flavonoids
638	4.58E+02	C22H34O10	3-Hydroxy-beta-ionol 3-(6''-Malonyl)Glucoside	Others
639	2.20E+02	C11H12N2O3	o-Carboxy-5-hydroxytryptamine	Alkaloids
640	3.12E+02	C14H16O8	1-O-Caffeoyl-β-D-xylose	Phenolic acids
641	3.31E+02	C20H17N3O2	Angustoline	Alkaloids
642	4.75E+02	C23H42NO7P	LysoPE 18:3(2n isomer)	Lipids
643	4.73E+02	C20H23N7O7	10-Formyltetrahydrofolic Acid	Alkaloids
644	1.45E+02	C7H15NO2	(S)-2-Aminoheptanoic acid	Nucleotides and derivatives
645	2.90E+02	C19H30O2	Methyl 12-phenyldodecanoate	Lipids
646	1.46E+02	C9H6O2	Coumarin	Lignans and Coumarins
647	3.03E+02	C11H21N5O5	Glu-Arg	Amino acids and derivatives
648	2.36E+02	C12H16N2O3	L-Alanyl-L-Phenylalanine	Amino acids and derivatives
649	3.56E+02	C15H16O10	O-p-Coumaroylgalactaric acid	Phenolic acids
650	2.32E+02	C15H20O2	3,5,11(13)-Trieneudesma-13-oic acid	Terpenoids
651	2.60E+02	C10H20N4O4	Lys-Asn	Amino acids and derivatives
652	1.80E+02	C11H16O2	oxyphyllone F	Terpenoids
653	1.53E+02	C7H7NO3	3-Aminosalicylic acid	Phenolic acids
654	2.90E+02	C14H14N2O5	Indole-3-acetyl-L-aspartic acid	Alkaloids
655	9.42E+02	C47H74O19	Polygalasaponin E	Terpenoids
656	3.76E+02	C16H24O10	5β,6β-Dihydroxyboschnaloside*	Terpenoids
657	2.94E+02	C17H26O4	Nordihydrocapsiate	Phenolic acids
658	1.54E+02	C7H6O4	2,4-Dihydroxybenzoic acid	Phenolic acids

659	3.84E+02	C14H20N6O7	Asp-His-Asn	Amino acids and derivatives
660	2.89E+02	C16H35NO3	2-Aminohexadecane-1,16,16-triol	Others
661	3.10E+02	C18H30O4	9-Hydroxy-12-oxo-10(E),15(Z)-octadecadienoic acid	Lipids
662	2.90E+02	C18H26O3	14(15)-Bisnor-13-oxolabd-8(17),11(E)-dien-19-oic acid	Terpenoids
663	2.90E+02	C18H26O3	(9Z,11E,13E,15Z)-4-Oxo-9,11,13,15-Octadecatetraenoic Acid	Lipids
664	2.00E+02	C12H24O2	Dodecanoic acid (Lauric acid)	Lipids
665	9.56E+02	C48H76O19	Quinovic acid-3-O-β-D-glucosyl-(1→4)-α-L-rhamnoside-28-O-β-D-glucoside*	Terpenoids
666	2.90E+02	C18H26O3	Normelanothyrsin A	Terpenoids
667	1.32E+02	C5H8O4	Ethylmalonic acid*	Organic acids
668	1.45E+02	C9H7NO	Indole-3-carboxaldehyde	Alkaloids
669	2.80E+02	C14H20N2O4	Tyr-Val	Amino acids and derivatives
670	1.80E+02	C11H16O2	5,6,7,7a-tetrahydro-4,4,7a-trimethyl-2(4H)-benzofuranone	Others
671	1.80E+02	C11H16O2	2,5-dimethyl-2,3,3a,7a-tetrahydro-1H-indene-1,3-diol	Terpenoids
672	3.56E+02	C16H20O9	1-O-Feruloyl-β-D-glucose*	Phenolic acids
673	1.88E+02	C9H20N2O2	Trimethyllysine	Amino acids and derivatives
674	3.56E+02	C13H20N6O6	His-Ser-Asn	Amino acids and derivatives
675	1.31E+02	C5H9NO3	N-Propionylglycine	Amino acids and derivatives
676	3.00E+02	C18H36O3	DL-2-hydroxystearic acid*	Lipids
677	3.40E+02	C15H16O9	Esculin (6,7-Dihydroxycoumarin-6-O-glucoside)*	Lignans and Coumarins
678	4.22E+02	C12H23O14P	Trehalose 6-phosphate	Others
679	3.72E+02	C20H20O7	Tangeretin (4',5,6,7,8-Pentamethoxyflavone)*	Flavonoids
680	1.78E+02	C10H10O3	p-Coumaric acid methyl ester	Phenolic acids
681	2.98E+02	C14H18O7	Picein (4-Acetylphenyl-glucoside)	Phenolic acids
682	4.67E+02	C22H46NO7P	LysoPC 14:0	Lipids
683	5.64E+02	C27H32O13	Pinocembrin-7-O-neohesperidoside	Flavonoids
684	5.50E+02	C27H34O12	5'-Methoxymatairesinoside	Lignans and Coumarins
685	2.06E+02	C11H10O4	4-Methoxy-5-(hydroxymethyl)-2H-1-benzopyran-2-one*	Lignans and Coumarins
686	1.48E+02	C5H8O5	D-Arabinono-1,4-lactone*	Others
687	2.04E+02	C12H12O3	Senkyunolide B	Others

688	1.22E+02	$C_7H_6O_2$	2-Hydroxybenzaldehyde (Salicylaldehyde)	Phenolic acids
689	5.81E+02	$C_{28}H_{56}NO_9P$	1-(2,3-dihydroxypropoxy)-3-(((2-(dimethylamino)ethoxy)(hydroxy)phosphoryl)oxy)propan-2-yl (Z)-14-Octadecenoic Acid	Others
690	3.12E+02	$C_{18}H_{32}O_4$	13S-Hydroperoxy-9Z,11E-octadecadienoic acid	Lipids
691	6.52E+02	$C_{29}H_{32}O_{17}$	Kaempferol-3-O-(6''-Acetyl)glucosyl-(1→3)-Galactoside	Flavonoids
692	1.66E+02	$C_8H_6O_4$	Phthalic acid	Phenolic acids
693	3.42E+02	$C_{15}H_{18}O_9$	6-O-Caffeoyl-D-glucose*	Phenolic acids
694	1.47E+02	$C_5H_9NO_4$	L-threo-3-Methylaspartate	Amino acids and derivatives
695	4.14E+02	$C_{24}H_{30}O_6$	Myrtucommulone B	Others
696	3.36E+02	$C_{16}H_{16}O_8$	3-O-caffeoylshikimic acid*	Phenolic acids
697	2.98E+02	$C_{18}H_{34}O_3$	Ricinoleic acid	Lipids
698	8.51E+01	$C_5H_{11}N$	Piperidine	Alkaloids
699	1.88E+02	$C_9H_{16}O_4$	Azelaic acid	Organic acids
700	5.04E+02	$C_{30}H_{48}O_6$	2α,3β,23,29-tetrahydroxy olean-12-en-28-oic acid	Terpenoids
701	3.11E+02	$C_{14}H_{17}NO_7$	5,6-Dihydroxyindole-5-O-β-glucoside	Alkaloids
702	3.42E+02	$C_{15}H_{18}O_9$	Grevilloside Q	Others
703	4.33E+02	$C_{19}H_{23}N_5O_7$	His-Asp-Tyr	Amino acids and derivatives
704	1.46E+02	$C_6H_{10}O_4$	3-Hydroxy-3-Methyl-2-Oxopentanoic Acid*	Organic acids
705	2.78E+02	$C_{18}H_{30}O_2$	Punicic acid (9Z,11E,13Z-octadecatrienoic acid)	Lipids
706	4.70E+02	$C_{31}H_{50}O_3$	3-Hydroxy-24-methylene-9,19-cyclolanostan-26-oic acid (Ambolic acid)*	Terpenoids
707	4.70E+02	$C_{31}H_{50}O_3$	Methyl oleanolate*	Terpenoids
708	3.02E+02	$C_{15}H_{18}N_4O_3$	His-Phe	Amino acids and derivatives
709	5.04E+02	$C_{18}H_{32}O_{16}$	D-Melezitose	Others
710	4.19E+02	$C_{20}H_{19}O_{10}^+$	Cyanidin-3-O-arabinoside	Flavonoids
711	2.08E+02	$C_{13}H_{20}O_2$	3R-3-hydroxy-β-ionone	Terpenoids
712	3.42E+02	$C_{16}H_{22}O_8$	Coniferin	Phenolic acids
713	4.86E+02	$C_{30}H_{46}O_5$	3-Hydroxyolean-12-ene-27,28-dioic acid (Cincholic acid)	Terpenoids
714	2.26E+02	$C_{11}H_{14}O_5$	3,4'-Dihydroxy-3',5'-dimethoxypropiophenone	Others
715	2.07E+02	$C_{11}H_{13}NO_3$	N-Acetyl-L-phenylalanine	Amino acids and derivatives
716	3.72E+02	$C_{16}H_{20}O_{10}$	Quinacyl syringic acid	Phenolic acids
717	2.62E+02	$C_{14}H_{18}N_2O_3$	L-Prolyl-L-Phenylalanine	Amino acids and derivatives
718	2.34E+02	$C_{15}H_{22}O_2$	polygodial	Terpenoids
719	4.72E+02	$C_{30}H_{48}O_4$	2,3-Dihydroxyolean-12-en-28-oic acid (2-Hydroxyoleanolic acid)*	Terpenoids

720	2.68E+02	C9H16O9	Keto-Deoxy-Nonulonic acid	Organic acids
721	3.96E+02	C17H32O10	1-Hexanol arabinosylglucoside	Others
722	6.36E+02	C35H40O11	Schisantherin J	Lignans and Coumarins
723	3.14E+02	C18H34O4	Hydroxy ricinoleic acid	Lipids
724	1.55E+02	C6H9N3O2	2-amino-3-(1H-pyrazol-1-yl)propanoic acid	Organic acids
725	6.52E+02	C30H36O16	Aeschynanthoside A	Phenolic acids
726	6.78E+02	C33H58O14	Gingerglycolipid B	Lipids
727	3.36E+02	C16H16O8	4-caffeoylshikimic acid*	Phenolic acids
728	4.02E+02	C21H22O8	5,6,7,3',4',5'-hexamethoxyflavone	Flavonoids
729	3.34E+02	C21H22N2O2	Strychnine	Alkaloids
730	6.84E+02	C31H40O17	7-O-(4''-O-glucosyl)coumaroyl-loganic acid	Terpenoids
731	3.83E+02	C14H17N5O8	Succinyladenosine	Nucleotides and derivatives
732	1.80E+02	C6H12O6	Inositol*	Others
733	1.68E+02	C8H8O4	4-Methoxysalicylic Acid	Phenolic acids
734	3.42E+02	C12H22O11	D-Lactose*	Others
735	1.42E+02	C8H14O2	Cyclohexaneacetic acid*	Organic acids
736	1.41E+02	C2H8NO4P	O-Phosphorylethanolamine	Alkaloids
737	2.32E+02	C15H20O2	Isoalantolactone	Terpenoids
738	6.78E+02	C31H34O17	1,4-O-di-Caffeoyl-3-O-glucoside Quinic Acid*	Phenolic acids
739	5.21E+02	C26H52NO7P	LysoPC 18:1(2n isomer)*	Lipids
740	4.70E+02	C30H46O4	23-Hydroxy-3-oxoolean-12-en-28-oic acid (Hederagonic acid)	Terpenoids
741	4.95E+02	C24H50NO7P	LysoPC 16:0(2n isomer)	Lipids
742	1.54E+02	C5H3ClN4	6-Chloropurine	Nucleotides and derivatives
743	7.00E+02	C37H52N2O11	Puberaconitidine	Alkaloids
744	1.92E+02	C13H20O	β-Ionone	Terpenoids
745	1.66E+02	C6H6N4O2	7-Methylxanthine	Nucleotides and derivatives
746	3.01E+02	C18H39NO2	Tetradecyldiethanolamine	Alkaloids
747	3.38E+02	C16H18O8	5-O-p-Coumaroylquinic acid*	Phenolic acids
748	3.68E+02	C17H20O9	Chlorogenic acid methyl ester	Phenolic acids
749	2.90E+02	C15H14O6	Epicatechin	Flavonoids
750	1.19E+02	C3H5NO4	Aminomalonic acid	Organic acids
751	4.88E+02	C30H48O5	2,3,23-Trihydroxyurs-12-en-28-oic acid (Asiatic acid)	Terpenoids
752	2.61E+02	C9H15N3O6	Glu-Asn	Amino acids and derivatives
753	1.89E+02	C10H7NO3	3-quinolinecarboxylic acid	Alkaloids
754	3.00E+02	C13H16O8	1-(4-Hydroxybenzoyl)Glucose; 25545-07-7	Phenolic acids
755	3.98E+02	C15H22N6O5S	S-(5'-Adenosyl)-L-methionine	Amino acids and derivatives

756	4.81E+02	C23H48NO7P	LysoPC 15:0*	Lipids
757	1.92E+02	C11H12O3	5-(2-hydroxypropyl)-3H-2-benzofuran-1-one	Others
758	4.52E+02	C20H20O12	Maleoyl-caffeoylquinic acid	Phenolic acids
759	2.05E+02	C11H11NO3	2-(Acetylamino)-3-phenyl-2-propenoic acid*	Alkaloids
760	4.72E+02	C30H48O4	2,3-Dihydroxy-12-ursen-28-oic acid*	Terpenoids
761	4.17E+02	C19H23N5O6	Phe-Asp-His	Amino acids and derivatives
762	1.64E+02	C9H8O3	4-hydroxyphenyl acrylaldehyde*	Others
763	3.10E+02	C18H30O4	13(s)-hydroperoxy-(9z,11e,15z)-octadecatrienoic acid	Lipids
764	3.82E+02	C24H30O4	(Z)-3,8-Dihydro 6,6·;7,3'a-diligustilide	Others
765	1.68E+02	C8H8O4	2,6-Dimethoxy-1,4-benzoquinone*	Quinones
766	3.90E+02	C16H22O11	Scandoside	Terpenoids
767	1.15E+02	C5H9NO2	3-hydroxy-1-methylpyrrolidin-2-one*	Alkaloids
768	1.18E+02	C4H6O4	Methylmalonic acid*	Organic acids
769	2.61E+02	C13H15N3O3	Glycyl-tryptophan	Amino acids and derivatives
770	3.00E+02	C18H36O3	12-Hydroxyoctadecanoic acid	Lipids
771	3.88E+02	C17H24O10	Majoroside	Terpenoids
772	1.36E+02	C8H12N2	2,3,5,6-Tetramethylpyrazine; Ligustrazine	Alkaloids
773	2.28E+02	C7H4N2O7	2-Hydroxy-3,5-dinitrobenzoic acid	Phenolic acids
774	1.36E+02	C4H8O5	D-Threonic Acid	Others
775	5.36E+02	C26H32O12	Nortrachelogenin-4-O-glucoside	Lignans and Coumarins
776	6.02E+02	C31H38O12	Senecioyloxychaparrinone triacetate	Terpenoids
777	1.44E+02	C7H16N2O	N-Acetylcadaverine	Alkaloids
778	2.34E+02	C15H22O2	Procurcumenol	Terpenoids
779	2.76E+02	C12H20O7	Triethyl citrate	Organic acids
780	4.88E+02	C30H48O5	Cannabifolin F	Terpenoids
781	5.04E+02	C24H40O11	(6R,9R)-megastigman-4-en-9-ol-3-one O-β-D-(6'-O-β-D-apiofuranosyl)glucopyranoside	Terpenoids
782	1.39E+02	C6H5NO3	6-Hydroxynicotinic acid	Alkaloids
783	3.38E+02	C16H18O8	Coumaroyl Quinic Acid	Others
784	2.75E+02	C10H17N3O6	gamma-Glutamylglutamine	Amino acids and derivatives
785	3.45E+02	C10H12N5O7P	Guanosine 3',5'-cyclic monophosphate	Nucleotides and derivatives
786	3.56E+02	C19H16O7	5,7,5'-Trimethoxy-3',4'-methylenedioxyflavonoid	Flavonoids
787	3.99E+02	C15H21N5O8	Ribosyladenosine	Nucleotides and derivatives
788	5.34E+02	C26H30O12	Sesamolinol-glucoside	Lignans and Coumarins

789	2.49E+02	C8H12NO6P	Pyridoxine-5'-phosphate	Others
790	3.76E+02	C16H24O10	Mussaenosidic acid*	Terpenoids
791	3.86E+02	C20H18O8	5,6,7,5'-tetramethoxy-3',4'-methylenedioxyflavonoid	Flavonoids
792	3.99E+02	C15H23N6O5S+	S-Adenosylmethionine	Amino acids and derivatives
793	4.77E+02	C23H44NO7P	LysoPE 18:2	Lipids
794	1.23E+02	C6H5NO2	Isonicotinic acid	Others
795	2.86E+02	C15H10O6	Luteolin (5,7,3',4'-Tetrahydroxyflavone)*	Flavonoids
796	3.40E+02	C6H14O12P2	D-Glucose 1,6-bisphosphate	Others
797	1.58E+02	C8H15NO2	1-(Hydroxymethyl)hexahydro-1h-pyrrolizin-2-ol	Alkaloids
798	3.90E+02	C17H26O10	Dihydrocornin	Terpenoids
799	2.06E+02	C11H10O4	Scoparone	Lignans and Coumarins
800	5.77E+02	C28H52NO9P	2-(2,3-dihydroxypropoxy)-3-(((2-(dimethylamino)ethoxy)(hydroxy)phosphoryl)oxy)propyl (8E,11Z,14Z)-octadeca-8,11,14-trienoate	Others
801	5.23E+02	C26H54NO7P	LysoPC 18:0	Lipids
802	3.14E+02	C18H34O4	12,13-DHOME; (9Z)-12,13-Dihydroxyoctadec-9-enoic acid	Lipids
803	1.61E+02	C6H11NO4	O-Acetyl-L-homoserine	Amino acids and derivatives
804	1.16E+02	C5H8O3	3-Methyl-2-Oxobutanoic acid	Organic acids
805	1.69E+02	C7H11N3O2	1-Methylhistidine*	Amino acids and derivatives
806	3.00E+02	C13H16O8	4-O-Glucosyl-4-hydroxybenzoic acid*	Phenolic acids
807	5.05E+02	C25H48NO7P	LysoPE 20:2	Lipids
808	1.45E+02	C9H7NO	4-Hydroxyquinoline	Alkaloids
809	3.26E+02	C20H26N2O2	Hydroquinine	Alkaloids
810	2.92E+02	C19H32O2	Methyl linolenate	Lipids
811	5.18E+02	C24H38O12	Vomifoliol 9-[Xylosyl-(1->6)-Glucoside]	Terpenoids
812	3.74E+02	C16H22O10	Homosyringic Acid 4'-O-Glucoside	Phenolic acids
813	5.06E+02	C21H30O14	Hebitol II	Phenolic acids
814	2.05E+02	C11H11NO3	Methoxyindoleacetic acid	Alkaloids
815	2.90E+02	C18H26O3	4-Oxo-9Z,11Z,13E,15E-Octadecatetraenoic Acid	Lipids
816	2.18E+02	C15H22O	Nootkatone	Terpenoids
817	1.97E+02	C9H11NO4	3,4-Dihydroxy-L-phenylalanine (L-Dopa)	Amino acids and derivatives
818	4.72E+02	C30H48O4	Rubione B	Terpenoids
819	1.48E+02	C5H8O5	3-Methylmalic acid*	Organic acids
820	3.46E+02	C16H26O8	(2E,6Z)-2,6-Dimethyl-8-β-D-glucosyloxy-2,6-octadienoic acid	Others
821	5.55E+02	C26H54NO9P	1-(2,3-dihydroxypropoxy)-3-(((2-(dimethylamino)ethoxy)(hydroxy)phosphoryl)oxy)propan-2-yl	Others

			palmitate*	
822	1.29E+02	C5H7NO3	1-Pyrroline-4-hydroxy-2-carboxylic acid	Organic acids
823	2.36E+02	C16H12O2	6-Methylflavone	Flavonoids
824	3.00E+02	C18H36O3	3-Hydroxyoctadecanoic Acid	Lipids
825	1.53E+02	C7H7NO3	3-Hydroxyanthranilic acid	Alkaloids
826	1.78E+02	C9H6O4	Esculetin (6,7-Dihydroxycoumarin)	Lignans and Coumarins
827	1.34E+02	C8H6O2	2(4H)-benzofuranone	Others
828	1.24E+02	C6H4O3	Furan-2,5-dicarbaldehyde	Others
829	2.60E+02	C6H13O9P	D-Glucose 6-phosphate*	Others
830	2.45E+02	C10H19N3O4	Ile-Asn	Amino acids and derivatives
831	2.72E+02	C16H32O3	2-Hydroxyhexadecanoic acid	Organic acids
832	1.60E+02	C6H8O5	2-Oxoadipic acid	Organic acids
833	6.38E+02	C29H34O16	β-Oxoacteoside	Phenolic acids
834	2.78E+02	C16H22O4	1,3-Benzodioxole-5-nonanoic acid	Others
835	3.40E+02	C15H16O9	Esculetin-7-O-glucoside*	Lignans and Coumarins
836	2.22E+02	C11H10O5	isofraxidin	Lignans and Coumarins
837	4.42E+02	C22H18O10	Epicatechin gallate	Flavonoids
838	1.51E+02	C5H5N5O	Guanine	Nucleotides and derivatives
839	1.63E+02	C6H13NO2S	L-Homomethionine	Amino acids and derivatives
840	3.73E+02	C17H31N3O6	Leu-Ile-Glu	Amino acids and derivatives
841	3.21E+02	C11H19N3O6S	S-(Methyl)glutathione	Amino acids and derivatives
842	4.52E+02	C30H44O3	3-Oxooleana-11,13(18)-dien-28-oic acid	Terpenoids
843	6.10E+02	C27H30O16	Kaempferol-3-O-sophoroside	Flavonoids
844	2.10E+02	C11H14O4	3-(2,5-dimethoxyphenyl)propanoic acid	Phenolic acids
845	3.82E+02	C16H30O10	3-Methylbutyl 6-O-(alpha-L-arabinopyranosyl)-beta-D-glucopy ranoside	Others
846	1.03E+02	C5H13NO	L-Valinol	Amino acids and derivatives
847	1.56E+02	C5H4N2O4	Orotic acid (Vitamin B13)	Others
848	3.02E+02	C17H34O4	MG(0:0/14:0/0:0)	Lipids
849	3.62E+02	C22H34O4	19,20-DiHDPA	Lipids
850	2.06E+02	C13H18O2	4-Hydroxy-3,5-diisopropylbenzaldehyde	Phenolic acids
851	2.29E+02	C14H31NO	2-Aminotetradecan-1-ol	Others
852	9.31E+01	C6H7N	Aniline	Alkaloids
853	5.22E+02	C26H34O11	(7R,8S)-dihydrodehydrodiconiferyl alcohol	Lignans and

			9-O-β-D-glucopyranoside	Coumarins
854	2.46E+02	C13H14N2O3	N-Acetyl-L-Tryptophan	Amino acids and derivatives
855	3.62E+02	C22H34O4	Ethyl (4E,6E,8E,10E,13Z)-15,16-dihydroxy-5,9-dimethyloctadeca-4,6,8,10,13-pentaenoate	Others
856	2.55E+02	C16H33NO	Hexadecanamide	Alkaloids
857	1.76E+02	C7H12O5	2-Propylmalic Acid*	Organic acids
858	4.94E+02	C22H22O13	Laricitrin-3-O-glucoside	Flavonoids
859	4.56E+02	C20H24O12	Apiosylskimmin (Adicardin)	Lignans and Coumarins
860	1.78E+02	C5H10N2O3S	L-Cysteinyl-L-glycine	Amino acids and derivatives
861	1.24E+02	C7H8O2	4-Methylcatechol	Phenolic acids
862	4.54E+02	C30H46O3	Mudanpinoic acid A	Terpenoids
863	3.47E+02	C10H14N5O7P	Adenosine 5'-monophosphate	Nucleotides and derivatives
864	2.86E+02	C13H18O7	3-Hydroxy-5-Methylphenol-1-O-Glucoside	Phenolic acids
865	2.51E+02	C12H13NO5	N-carboxy-N-(2-oxo-2-phenylethyl)-L-alanine	Amino acids and derivatives
866	3.74E+02	C16H22O10	Gardoside*	Terpenoids
867	2.29E+02	C9H15N3O4	Pro-Asn	Amino acids and derivatives
868	1.23E+02	C6H5NO2	Nicotinic acid (Vitamin B3)	Others
869	1.20E+02	C8H8O	acetophenone	Others
870	5.36E+02	C26H32O12	1-Hydroxypinoresinol-4'-O-Glucoside	Lignans and Coumarins
871	4.56E+02	C30H48O3	Morolic acid*	Terpenoids
872	1.54E+02	C9H14O2	4-Oxo-2-nonenal	Others
873	3.15E+02	C15H25NO6	5-(4-(((S)-1-carboxyethyl)carbamoyl)cyclohexyl)-2-hydroxypentanoicacid	Alkaloids
874	2.72E+02	C12H16O7	p-Hydroxypheny-β-D-allopyranoside*	Phenolic acids
875	1.54E+02	C7H6O4	3,4-Dihydroxybenzoic acid (Protocatechuic acid)*	Phenolic acids
876	1.94E+02	C8H10N4O2	Caffeine	Alkaloids
877	3.10E+02	C18H30O4	13S-Hydroperoxy-6Z,9Z,11E-octadecatrienoic acid	Lipids
878	2.06E+02	C14H22O	2,6-Di-tert-butylphenol*	Phenolic acids
879	2.18E+02	C9H18N2O4	L-Seryl-L-Isoleucine	Amino acids and derivatives
880	2.94E+02	C18H30O3	Rabdosia acid A	Lipids
881	3.78E+02	C22H34O5	Vitexilactone	Terpenoids
882	1.69E+02	C7H11N3O2	3-Methyl-L-Histidine*	Amino acids and derivatives

883	3.31E+02	C14H21NO8	Pyridoxine-5'-O-glucoside	Others
884	3.72E+02	C17H24O9	Syringin	Phenolic acids
885	1.35E+02	C5H5N5	2-Aminopurine	Nucleotides and derivatives
886	1.45E+02	C7H15NO2	N,N-Dimethyl-L-Valine	Amino acids and derivatives
887	2.94E+02	C18H30O3	9-Hydroxy-10,12,15-octadecatrienoic acid*	Lipids
888	1.68E+02	C8H8O4	3-Hydroxy-4-methoxybenzoic acid; Isovanillic Acid	Phenolic acids
889	5.04E+02	C18H32O16	Maltotriose	Others
890	7.11E+01	C4H9N	Pyrrolidin	Alkaloids
891	2.86E+02	C11H18N4O3S	Met-His	Amino acids and derivatives
892	2.05E+02	C10H7NO4	4,8-Dihydroxyquinoline-2-carboxylic acid	Organic acids
893	1.64E+02	C10H12O2	Hinokitiol	Terpenoids
894	3.30E+02	C18H34O5	2-Hydroxy-4-methyl-3-undecanoyloxypentanoic acid methyl ester	Lipids
895	2.02E+02	C12H14N2O	L-Praziquanamine	Alkaloids
896	2.18E+02	C8H14N2O5	5-L-Glutamyl-L-amino acid	Amino acids and derivatives
897	5.07E+02	C25H50NO7P	LysoPC 17:1	Lipids
898	3.58E+02	C16H22O9	Dihydroferulic acid glucoside	Phenolic acids
899	1.05E+02	C3H7NO3	L-Serine	Amino acids and derivatives
900	2.86E+02	C13H18O7	Salicin	Phenolic acids
901	1.15E+02	C5H9NO2	Pterolactam	Alkaloids
902	2.34E+02	C13H18N2O2	p-Coumaroylputrescine	Alkaloids
903	2.90E+02	C15H14O6	Leucopelargonidin; 3,4,5,7,4'-Pentahydroxyflavan	Others
904	2.91E+02	C11H17NO8	N-Fructosyl Pyroglutamate	Amino acids and derivatives
905	1.60E+02	C7H12O4	2-Propylsuccinic acid*	Organic acids
906	1.46E+02	C9H6O2	Chromone	Others
907	2.80E+02	C18H32O2	Ethyl (10Z,13Z)-hexadeca-10,13-dienoate	Others
908	1.11E+02	C4H5N3O	Cytosine	Nucleotides and derivatives
909	5.76E+02	C30H24O12	Procyanidin A2	Tannins
910	6.16E+02	C30H32O14	Plumieride(Z)-P-Coumarate	Terpenoids
911	2.94E+02	C14H18N2O5	γ-Glutamylphenylalanine	Amino acids and derivatives
912	2.90E+02	C15H14O6	Catechin	Flavonoids
913	3.27E+02	C15H21NO7	N-benzoyl-2-aminoethyl-β-D-glucopyranoside	Alkaloids
914	4.39E+02	C20H42NO7P	LysoPE 15:0	Lipids
915	3.30E+02	C18H34O5	9,10,11-Trihydroxy-12-octadecenoic acid	Lipids

916	2.97E+02	C11H15N5O3S	5'-Deoxy-5'-(methylthio)adenosine	Nucleotides and derivatives
917	9.50E+01	C5H5NO	4-Hydroxypyridine	Alkaloids
918	1.80E+02	C10H12O3	Methyl-3-(3-hydroxyphenyl)Propionate	Phenolic acids
919	2.82E+02	C15H22O5	Dihydrophaseic acid	Terpenoids
920	1.46E+02	C5H6O5	3-Oxopentanedioic acid	Organic acids
921	1.54E+02	C8H10O3	3,4-Dimethoxyphenol	Phenolic acids
922	6.36E+02	C35H40O11	Chloramultilide D	Terpenoids
923	3.86E+02	C17H22O10	1-O-Glucosyl sinapate	Phenolic acids
924	3.36E+02	C21H36O3	Glycidyl Linoleate	Lipids
925	3.06E+02	C19H30O3	3β,6β-Dihydroxy-15-nor-14-oxo8(17),12-labdadien-14-a	Terpenoids
926	4.97E+02	C23H31N9O4	Trp-Arg-His	Amino acids and derivatives
927	6.73E+02	C36H51NO11	Veratridine	Alkaloids
928	1.20E+02	C8H8O	3-Methylbenzaldehyde*	Others
929	3.38E+02	C16H18O8	4-O-p-Coumaroylquinic acid	Phenolic acids
930	1.59E+02	C7H13NO3	5-Acetamidopentanoic Acid	Organic acids
931	5.20E+02	C26H32O11	Dehydrodiconiferylalcohol-9'-O-glucoside	Lignans and Coumarins
932	3.09E+02	C15H23N3O4	Lys-Tyr	Amino acids and derivatives
933	3.42E+02	C12H22O11	D-Cellobiose	Others
934	5.17E+02	C26H48NO7P	LysoPC 18:3	Lipids
935	5.78E+02	C27H30O14	Kaempferol-3,7-O-dirhamnoside (Kaempferitrin)	Flavonoids
936	4.53E+02	C21H44NO7P	LysoPE 16:0(2n isomer)	Lipids
937	4.48E+02	C21H20O11	Luteolin-3'-O-glucoside*	Flavonoids
938	1.12E+02	C5H4O3	3-Furoic acid	Organic acids
939	4.18E+02	C18H26O11	2-Hydroxyphenol-1-O-glucosyl(6→1)rhamnoside	Phenolic acids
940	1.78E+02	C6H10O6	4,5,6-Trihydroxy-2-oxohexanoic acid	Organic acids
941	4.16E+02	C18H24O11	Paeonicluside	Phenolic acids
942	2.17E+02	C12H27NO2	2-Aminododecane-1,4-diol	Others
943	1.52E+02	C8H8O3	Methyl 4-hydroxybenzoate*	Phenolic acids
944	1.52E+02	C8H8O3	Vanillin; 4-Hydroxy-3-Methoxybenzaldehyde*	Others
945	5.19E+02	C26H50NO7P	1-(9Z,12Z-Octadecadienoyl)-Sn-Glycero-3-Phosphocholine	Others
946	3.12E+02	C15H20O7	3,4-dihydroxy-allylbenzene-3-O-β-D-glucopyranoside	Phenolic acids
947	2.70E+02	C18H38O	1-Octadecanol	Lipids
948	1.52E+02	C7H8N2O2	1-Methyl-6-Oxo-1,6-Dihydropyridine-3-Carboxamide	Alkaloids
949	3.70E+02	C22H28NO4+	N-Methyltetrahydropalmatine	Alkaloids

950	5.30E+02	C26H26O12	Macranthoin G	Lignans and Coumarins
951	1.19E+02	C3H9N3O2	4-methyl-1,5,2,3-dioxadiazinan-2-amine	Others
952	2.78E+02	C18H30O2	Crepenynic acid	Lipids
953	1.88E+02	C8H16N2O3	L-Glycyl-L-isoleucine*	Amino acids and derivatives
954	2.30E+02	C11H22N2O3	L-Valyl-L-Leucine	Amino acids and derivatives
955	1.92E+02	C11H12O3	1,4-Benzodioxin-6-propanol	Others
956	4.48E+02	C19H28O12	2'-O-β-D-Glucopyranosylsalicin	Phenolic acids
957	1.29E+02	C5H7NO3	5-Oxo-L-Proline*	Amino acids and derivatives
958	2.87E+02	C16H33NO3	14-Amino-15-hydroxy-11-methylpentadecanoic acid	Others
959	2.94E+02	C18H30O3	13-KODE; (9Z,11E)-13-Oxooctadeca-9,11-dienoic acid*	Lipids
960	3.54E+02	C16H18O9	Neochlorogenic acid (5-O-Caffeoylquinic acid)*	Phenolic acids
961	1.62E+02	C8H6N2S	4-Phenyl-1,2,3-thiadiazole	Others
962	2.82E+02	C18H34O2	11-Octadecanoic acid(Vaccenic acid)*	Lipids
963	6.32E+02	C29H28O16	1,5-O-dicaffeoyl-3-O-maloyl-quinic acid	Phenolic acids
964	3.46E+02	C16H26O8	Kankanoside A	Terpenoids
965	3.26E+02	C16H22O7	Raspberryketone glucoside	Phenolic acids
966	3.30E+02	C14H18O9	1-O-Vanilloyl-D-Glucose	Phenolic acids
967	3.88E+02	C15H28N6O6	Arg-Asp-Val	Amino acids and derivatives
968	3.26E+02	C15H18O8	Melilotoside	Phenolic acids
969	4.48E+02	C19H28O12	3,4-Dihydroxyphenethyl alcohol-8-O-[β-D-apinosyl(1→3)]-β-D-glucoside	Phenolic acids
970	2.18E+02	C10H18O5	3-Hydroxydecanedioic acid	Organic acids
971	3.70E+02	C16H18O10	Fraxetin-8-O-glucoside (Fraxin)	Lignans and Coumarins
972	2.16E+02	C12H12N2O2	(R)-1,2,3,4-Tetrahydro-3-carboxy-2-carboline	Alkaloids
973	3.91E+02	C14H21N3O10	Asp-Glu-Glu	Amino acids and derivatives
974	3.44E+02	C15H20O9	2-β-D-Glucopyranosyloxy-5-hydroxyphenylacetic acidmethylester*	Phenolic acids
975	1.94E+02	C10H10O4	Ferulic acid*	Phenolic acids
976	4.91E+02	C24H46NO7P	LysoPC 16:2(2n isomer)	Lipids
977	1.36E+02	C8H8O2	4-Hydroxyacetophenone	Others
978	2.60E+02	C15H20N2O2	Baptifoline	Alkaloids
979	1.03E+02	C4H9NO2	2-Aminoisobutyric acid*	Organic acids
980	4.86E+02	C30H46O5	27,28-Dicarboxyl ursolic acid	Terpenoids
981	2.24E+02	C14H8O3	1-Hydroxyanthraquinone	Quinones
982	2.46E+02	C13H14N2O3	N-acetyl-tryptophan	Amino acids and

				derivatives
983	1.01E+02	C4H7NO2	5-Hydroxy-2-pyrrolidinone	Alkaloids
984	1.66E+02	C8H6O4	5,7-Dihydroxy-1(3H)-isobenzofuranone*	Phenolic acids
985	1.20E+02	C8H8O	Phenylacetaldehyde	Phenolic acids
986	5.94E+02	C27H30O15	Kaempferol-3-O-rutinoside(Nicotiflorin)	Flavonoids
987	5.94E+02	C27H30O15	Safflor yellow A	Quinones
988	4.86E+02	C30H46O5	2α,3α,23-Trihydroxyurs-12,20(30)-dien-28-oic acid	Terpenoids
989	5.26E+02	C24H30O13	Guaiacylglycerol 8-O-(1''-O-Glucosyl)Vanillic Acid Ether	Phenolic acids
990	1.68E+02	C8H8O4	3,4-Dihydroxybenzeneacetic acid*	Phenolic acids
991	3.37E+02	C12H23N3O4S2	Met-Gly-Met	Amino acids and derivatives
992	3.72E+02	C19H24N4O4	Ala-Pro-Trp	Amino acids and derivatives
993	1.35E+02	C4H9NO2S	S-Methyl-L-cysteine	Amino acids and derivatives
994	1.76E+02	C10H8O3	2-Methoxy-1-benzofuran-5-carbaldehyde	Others
995	4.92E+02	C24H28O11	picroside Ⅰ	Others
996	3.28E+02	C18H32O5	9,12,13-Trihydroxy-10,15-octadecadienoic acid	Lipids
997	2.62E+02	C9H14N2O7	Asp-Glu	Amino acids and derivatives
998	7.00E+02	C32H44O17	Yadanzioside J	Terpenoids
999	4.48E+02	C21H20O11	Carthamone*	Flavonoids
1000	4.48E+02	C21H20O11	Kaempferol-3-O-galactoside (Trifolin)*	Flavonoids
1001	6.28E+02	C35H36N2O9	Thapsakon A	Alkaloids
1002	5.36E+02	C25H28O13	Syringic acid-4-O-(6''-feruloyl)glucoside	Phenolic acids
1003	4.81E+02	C24H27N5O6	His-Tyr-Tyr	Amino acids and derivatives
1004	4.48E+02	C19H28O12	Hebitol I	Others
1005	4.58E+02	C30H50O3	Rubiatriol	Terpenoids
1006	2.44E+02	C9H12N2O6	Uridine	Nucleotides and derivatives
1007	1.88E+02	C10H20O3	2,6-Dimethyl-7-octene-2,3,6-triol	Others
1008	5.22E+02	C26H34O11	Dihydrodehydrodiconiferyl alcohol-4-O-glucoside*	Lignans and Coumarins
1009	5.22E+02	C26H34O11	Isolariciresinol-9'-O-glucoside*	Lignans and Coumarins
1010	3.38E+02	C20H18O5	Cagayanone A	Lignans and Coumarins
1011	1.72E+02	C9H16O3	9-Oxononanoic acid	Organic acids
1012	1.95E+02	C10H13NO3	L-Tyrosine methyl ester	Amino acids and derivatives
1013	4.50E+02	C21H22O11	Aromadendrin-7-O-glucoside	Flavonoids

1014	1.47E+02	C9H9NO	(E)-Cinnamamide	Alkaloids
1015	4.74E+02	C22H34O11	Dihydrovomifoliol 4-O-malonyl glucoside	Others
1016	6.16E+02	C30H32O14	13-O-p-Coumaroylplumieride	Terpenoids
1017	3.78E+02	C16H26O10	Lamiol	Terpenoids
1018	4.80E+02	C21H20O13	Quercetagetin-7-O-glucoside(Quercetagitrin)*	Flavonoids
1019	3.50E+02	C16H18N2O5S	Indole-3-cyano-2-O-glucoside	Alkaloids
1020	2.64E+02	C14H20N2O3	Val-Phe	Amino acids and derivatives
1021	2.94E+02	C12H22O8	Glucosyl 2-Hydroxy-4-Methylpentanoic Acid	Organic acids
1022	3.02E+02	C15H18N4O3	Phe-His	Amino acids and derivatives
1023	4.88E+02	C30H48O5	16,23-Epoxy-11,24,25-trihydroxydammar-13(17)-en-3-one (Alisol F)	Terpenoids
1024	3.74E+02	C16H22O10	Secologaoside	Others
1025	2.68E+02	C8H16N2O4S2	L-Homocystine	Amino acids and derivatives
1026	2.96E+02	C18H32O3	12,13-Epoxy-9-Octadecenoic Acid	Lipids
1027	6.28E+02	C35H36N2O9	Thapsakon B	Alkaloids
1028	3.43E+02	C15H21NO8	N-(1-Deoxy-1-fructosyl)Tyrosine	Amino acids and derivatives
1029	4.70E+02	C30H46O4	3,4-Secodammara-4(28),20,24-trien-3,26-dioic acid	Terpenoids
1030	4.92E+02	C23H24O12	Protocatechuic acid 4-O-(6''-O-Feruloyl)Glucoside	Phenolic acids
1031	4.22E+02	C24H38O6	(2'R)-6-(2'-Acetoxypentadecyl)-5-hydroxy-2-methoxy-1,4-benzoquinone-1	Quinones
1032	2.22E+02	C11H14N2O3	Glycylphenylalanine*	Amino acids and derivatives
1033	1.90E+02	C10H6O4	Coumarin-3-carboxylic Acid	Lignans and Coumarins
1034	2.86E+02	C20H30O	Pimaradienone*	Terpenoids
1035	4.08E+02	C20H24O9	Nodakenin	Lignans and Coumarins
1036	1.64E+02	C10H12O2	4-Phenylbutyric acid	Organic acids
1037	2.94E+02	C15H22N2O4	Tyrosylleucine	Amino acids and derivatives
1038	4.25E+02	C19H40NO7P	LysoPE 14:0*	Lipids
1039	5.79E+02	C28H54NO9P	2-(2,3-dihydroxypropoxy)-3-(((2-(dimethylamino)ethoxy)(hydroxy)phosphoryl)oxy)propyl (11Z,14Z)-octadeca-11,14-dienoate	Others
1040	3.11E+02	C13H21N5O4	Val-Gly-His	Amino acids and derivatives
1041	1.02E+03	C49H78O22	Yamogenin-3-O-glucosyl(1→2)[xylosyl(1→3)]glucosyl(1→4)galactoside	Steroids

1042	1.25E+02	C2H7NO3S	2-Aminoethanesulfonic acid	Organic acids
1043	4.74E+02	C30H50O4	3,13,15-Trihydroxyoleanane-12-one	Terpenoids
1044	1.46E+02	C5H6O5	α-Ketoglutaric acid	Organic acids
1045	1.88E+02	C7H12N2O4	N-Acetyl-L-Glutamine	Amino acids and derivatives
1046	4.72E+02	C30H48O4	2,3-Dihydroxyurs-12-en-29-oic acid (Maslinic acid)*	Terpenoids
1047	5.22E+02	C25H30O12	7-O-coumaroyl-loganic acid	Terpenoids
1048	3.54E+02	C16H18O9	Chlorogenic acid (3-O-Caffeoylquinic acid)*	Phenolic acids
1049	4.61E+02	C19H27NO12	Anthranilate-1-O-Sophoroside	Phenolic acids
1050	1.51E+02	C5H5N5O	Isoguanine	Nucleotides and derivatives
1051	1.22E+02	C6H6N2O	Isonicotinamide	Others
1052	2.56E+02	C11H14NO6+	Nicotinate D-ribonucleoside	Others
1053	4.78E+02	C23H26O11	1'-O-(3,4-Dihydroxyphenethyl)-O-caffeoyl-glucoside	Phenolic acids
1054	1.36E+02	C5H4N4O	Allopurinol	Nucleotides and derivatives
1055	2.76E+02	C10H16N2O7	L-α-Glutamyl-L-Glutamic Acid	Amino acids and derivatives
1056	4.51E+02	C21H42NO7P	LysoPE 16:1(2n isomer)*	Lipids
1057	3.87E+02	C15H25N5O5S1	Met-Thr-His	Amino acids and derivatives
1058	3.39E+02	C15H17NO8	6-beta-d-glucopyranosyloxyindole-3-carboxylic acid	Alkaloids
1059	2.78E+02	C16H22O4	Dibutyl phthalate*	Phenolic acids
1060	1.61E+02	C9H7NO2	Indole-5-carboxylic acid*	Alkaloids
1061	3.68E+02	C18H24O8	3,4,5-Trihydroxy-6-[4-(4-methyl-3-oxopentyl)phenoxy]oxane-2-carboxylic acid	Phenolic acids
1062	1.54E+02	C7H6O4	2,3-Dihydroxybenzoic Acid*	Phenolic acids
1063	2.82E+02	C16H26O4	6-Hydroxyphomodiol	Others
1064	4.79E+02	C23H46NO7P	LysoPE 18:1*	Lipids
1065	2.00E+02	C4H9O7P	D-Erythrose-4-phosphate	Others
1066	3.38E+02	C16H18O8	1,4,8-Trihydroxynaphthalene-1-O-glucoside*	Quinones
1067	3.08E+02	C19H32O3	Sessilifol O	Terpenoids
1068	1.84E+02	C9H12O4	Antiarol; 3,4,5-Trimethoxyphenol	Phenolic acids
1069	5.00E+02	C22H28O13	1-O-rhamnose-3-O-Caffeoyl Quinic Acid	Phenolic acids
1070	4.75E+02	C20H29NO12	2-Glucosyl-glucosyloxy-2-phenylacetic acid amide	Alkaloids
1071	5.05E+02	C25H48NO7P	LysoPC 17:2	Lipids
1072	5.66E+02	C15H24N2O17P2	Uridine 5'-diphospho-D-glucose	Nucleotides and derivatives
1073	4.31E+02	C20H29N7O4	Ala-Arg-Trp	Amino acids and derivatives

1074	5.16E+02	C22H28O14	4-O-(4'-O-alpha-D-Glucopyranosyl)caffeoylquinic acid	Phenolic acids
1075	2.02E+02	C8H18N4O2	N,N'-Dimethylarginine;SDMA*	Amino acids and derivatives
1076	3.20E+02	C17H20O6	Guaiacylglycerol-β-Guaiacyl Ether	Lignans and Coumarins
1077	1.82E+02	C9H10O4	3-(3-Hydroxyphenyl)-3-hydroxypropanoic acid	Phenolic acids
1078	1.15E+02	C5H9NO2	4-Methylazetidine-2-Carboxylic acid*	Alkaloids
1079	1.68E+02	C3H5O6P	Phosphoenolpyruvate	Organic acids
1080	1.74E+02	C7H14N2O3	N-α-Acetyl-L-ornithine	Amino acids and derivatives
1081	1.44E+02	C10H8O	1-Naphthol*	Phenolic acids
1082	3.34E+02	C11H18N4O8	Ser-Asp-Asn	Amino acids and derivatives
1083	3.31E+02	C14H21NO8	Hydroxymenisdaurin D	Alkaloids
1084	2.67E+02	C10H13N5O4	Adenosine*	Nucleotides and derivatives
1085	3.11E+02	C12H17N5O5	2-(Dimethylamino)guanosine*	Nucleotides and derivatives
1086	3.11E+02	C12H17N5O5	N6-(2-Hydroxyethyl)adenosine*	Nucleotides and derivatives
1087	2.21E+02	C8H15NO6	N-Acetyl-D-glucosamine	Others
1088	5.36E+02	C26H32O12	1-Hydroxypinoresinol-1-O-Glucoside	Lignans and Coumarins
1089	1.81E+02	C9H11NO3	DL-O-tyrosine	Amino acids and derivatives
1090	2.34E+02	C15H22O2	Aspergillusene A	Terpenoids
1091	6.54E+02	C31H58O14	2-Palmitoyl-Sn-Glycerol 3-O-Diglucoside	Lipids
1092	3.60E+02	C22H32O4	Moniliferanone D	Others
1093	6.38E+02	C30H38O15	4-Dihydroxyphenethoxy-8-O-β-D-[6-O-(4-O-β-D-glucopyranosyl)-feruloyl]glucopyranoside	Phenolic acids
1094	4.65E+02	C21H21O12+	Delphinidin-3-O-glucoside (Mirtillin)	Flavonoids
1095	1.51E+02	C7H5NOS	2(3H)-Benzothiazolone	Alkaloids
1096	3.42E+02	C12H22O11	D-Maltose*	Others
1097	3.91E+02	C11H21NO10S2	2-Hydroxy-2-methylpropyl glucosinolate	Others
1098	2.67E+02	C9H17NO8	2-Amino-3,4-dihydroxybutanoic acid-3-O-arabinoside	Amino acids and derivatives
1099	1.64E+02	C10H12O2	Eugenol	Phenolic acids
1100	1.38E+02	C7H6O3	4-Hydroxybenzoic acid	Phenolic acids
1101	1.34E+02	C4H6O5	D-Malic acid*	Organic acids
1102	3.58E+02	C20H38O5	2-Hydroxy-4-methyl-3-tridecanoyloxypentanoic acid methyl ester	Lipids
1103	3.26E+02	C15H18O8	1-O-p-Coumaroyl-β-D-glucose	Phenolic acids
1104	1.34E+02	C4H6O5	L-Malic acid*	Organic acids

1105	5.47E+02	C28H54NO7P	LysoPC 20:2*	Lipids
1106	1.92E+02	C10H8O4	6-Hydroxy-7-methoxycoumarin*	Lignans and Coumarins
1107	1.72E+02	C10H20O2	Decanoic acid	Organic acids
1108	1.67E+02	C4H9NO4S	(2s)-2-Amino-4-sulfinobutanoic acid	Amino acids and derivatives
1109	2.78E+02	C15H22N2O3	Ile-Phe	Amino acids and derivatives
1110	1.89E+02	C7H11NO5	N-Acetyl-L-glutamic acid	Amino acids and derivatives
1111	4.84E+02	C22H45O9P	LysoPG 16:0	Lipids
1112	3.05E+02	C15H19N3O4	Thr-Trp	Amino acids and derivatives
1113	2.16E+02	C8H16N4O3	N-Acetyl-L-Arginine	Amino acids and derivatives
1114	1.92E+02	C10H8O4	6,8-Dihydroxy-3-methylisocoumarin	Lignans and Coumarins
1115	1.35E+02	C5H5N5	Adenine	Nucleotides and derivatives
1116	2.08E+02	C11H12O4	(R)-3-ethyl-7-hydroxy-6-methoxyphthalide	Others
1117	2.38E+02	C11H14N2O4	Tyr-Gly*	Amino acids and derivatives
1118	4.02E+02	C18H26O10	Benzyl-(2''-O-xylosyl)glucoside*	Phenolic acids
1119	3.63E+02	C10H14N5O8P	Guanosine 5'-monophosphate	Nucleotides and derivatives
1120	1.34E+02	C4H6O5	3-Dehydro-L-Threonic Acid*	Others
1121	5.80E+02	C28H36O13	Syringaresinol-4'-O-glucoside; Acanthoside B	Lignans and Coumarins
1122	5.16E+02	C22H28O14	4-O-(3'-O-alpha-D-Glucopyranosyl)caffeoylquinic acid	Phenolic acids
1123	2.61E+02	C9H12NO6P	O-phosphate-L-tyrosine	Amino acids and derivatives
1124	1.78E+02	C6H10O6	L-Gulono-1,4-Lactone*	Others
1125	1.41E+02	C6H11N3O	Histidinol	Alkaloids
1126	7.86E+02	C41H70O14	3,12,23,25-tetrahydroxy-20S,24S-epoxydammarane-3-O-xylosylglucoside	Terpenoids
1127	1.80E+02	C9H8O4	2,5-Dihydroxycinnamic acid	Phenolic acids
1128	2.36E+02	C10H20O6	Butyl beta-D-glucoside	Others
1129	1.48E+02	C8H4O3	Phthalic anhydride	Phenolic acids
1130	3.06E+02	C20H34O2	11,14,17-Eicosatrienoic acid*	Lipids
1131	3.06E+02	C20H34O2	Dihomo-gamma-linolenic acid; (8Z,11Z,14Z)-Icosatrienoic acid*	Lipids
1132	1.88E+02	C10H20O3	10-Hydroxydecanoic acid	Lipids
1133	4.70E+02	C30H46O4	Virgatic acid	Terpenoids

1134	1.82E+02	C6H14O6	Dulcitol*	Others
1135	2.16E+02	C12H24O3	12-Hydroxydodecanoic acid	Lipids
1136	5.04E+02	C21H28O14	1-O-Caffeoyl-(6-O-glucosyl)-β-D-glucose	Phenolic acids
1137	4.42E+02	C30H50O2	3,28-Dihydroxylup-20(29)-ene (Betulin)	Terpenoids
1138	1.82E+02	C9H10O4	3,5-Dimethoxy-4-hydroxybenzaldehyde	Others
1139	2.94E+02	C18H30O3	13S-Hydroxy-9Z,11E,15Z-octadecatrienoic acid	Lipids
1140	2.64E+02	C14H20N2O3	L-Valyl-L-Phenylalanine	Amino acids and derivatives
1141	2.78E+02	C18H30O2	γ-Linolenic Acid*	Lipids
1142	4.61E+02	C22H31N5O6	Trp-Glu-Lys	Amino acids and derivatives
1143	6.66E+02	C24H42O21	Stachyose	Others
1144	4.72E+02	C30H48O4	16,23:16,30-Diepoxydammar-24-ene-3,20-diol (Jujubogenin)*	Terpenoids
1145	3.82E+02	C16H22N4O7	Tyr-Ser-Asn	Amino acids and derivatives
1146	1.74E+02	C6H10N2O4	N-Alpha-Acetyl-L-Asparagine	Amino acids and derivatives
1147	3.02E+02	C20H30O2	Pimaric acid*	Terpenoids
1148	4.88E+02	C21H28O13	Cistanoside F	Phenolic acids
1149	3.31E+02	C15H29N3O5	Ser-Leu-Leu	Amino acids and derivatives
1150	4.27E+02	C10H15N5O10P2	Adenosine 5'-diphosphate	Nucleotides and derivatives
1151	6.66E+02	C28H42O18	2,5-Dihydroxyphenylacetate ethyl triglucoside	Phenolic acids
1152	2.68E+02	C16H12O4	6-Hydroxy-2'-methoxyflavone	Flavonoids
1153	1.19E+02	C8H9N	N-Benzylmethylene isomethylamine	Alkaloids
1154	3.12E+02	C20H40O2	Arachidic acid	Lipids
1155	1.31E+02	C5H9NO3	N-acetyl-beta-alanine	Amino acids and derivatives
1156	2.06E+02	C11H10O4	(Z)-3-Ethylidene-7-hydroxy-6-methoxyphthalide*	Others
1157	2.31E+02	C8H17N5O3	Arg-Gly	Amino acids and derivatives
1158	1.80E+02	C9H8O4	3-(3,4-Dihydroxyphenyl)prop-2-enoic acid	Phenolic acids
1159	4.18E+02	C17H22O12	Protocatechuic acid xylosyl xyloside	Phenolic acids
1160	4.68E+02	C30H44O4	24-Hydroxy-3-oxooleana-11,13(18)-dien-28-oic acid	Terpenoids
1161	6.10E+02	C25H38O17	6'-O-β-D-glucosylbarlerin	Terpenoids
1162	6.07E+02	C17H27N3O17P2	Uridine-diphosphate-n-acetylgalactosamine	Nucleotides and derivatives
1163	2.18E+02	C8H14N2O5	Hyp-Ser	Amino acids and derivatives
1164	3.34E+02	C9H19O11P	1-(sn-Glycero-3-phospho)-1D-myo-inositol	Others

1165	3.30E+02	C18H34O5	9,10-Dihydroxy-12,13-epoxyoctadecanoic acid	Lipids
1166	3.76E+02	C16H24O10	Loganic acid*	Terpenoids
1167	5.00E+02	C28H36O8	Heteroclitin A	Lignans and Coumarins
1168	4.18E+02	C22H26O8	Lirioresinol A	Lignans and Coumarins
1169	3.56E+02	C20H20O6	Phellodensin D	Flavonoids
1170	4.52E+02	C21H24O11	Dihydromarein*	Flavonoids
1171	4.80E+02	C25H28N4O6	Phe-Trp-Glu	Amino acids and derivatives
1172	1.76E+02	C7H12O5	3-Isopropylmalic Acid*	Organic acids
1173	1.34E+02	C5H10O4	2,3-Dihydroxy-3-Methylbutanoic Acid	Organic acids
1174	1.67E+02	C7H5NS2	2-Mercaptobenzothiazole	Alkaloids
1175	1.89E+02	C11H11NO2	3-Indolepropionic acid	Alkaloids
1176	1.03E+02	C4H9NO2	(S)-3-Amino-2-methylpropanoic acid	Amino acids and derivatives
1177	4.70E+02	C30H46O4	2,3-Dihydroxyurs-12,18-dien-28-oic acid*	Terpenoids
1178	1.45E+02	C7H15NO2	N-Methylisoleucine	Amino acids and derivatives
1179	1.64E+02	C10H12O2	Frambinone	Others
1180	3.75E+02	C15H25N3O8	Ile-Asp-Glu	Amino acids and derivatives
1181	7.73E+02	C33H41O21+	Delphinidin-3-O-rutinoside-7-O-glucoside	Flavonoids
1182	3.64E+02	C15H24O10	Harpagide	Terpenoids
1183	1.33E+02	C8H7NO	2-(4-Hydroxyphenyl)acetonitrile	Phenolic acids
1184	3.44E+02	C15H20O9	Dihydrocaffeoylglucose*	Phenolic acids
1185	3.60E+02	C18H16O8	Myricetin-3,7,3'-trimethyl ether	Flavonoids
1186	3.16E+02	C13H16O9	1-O-Galloyl-rhamnose	Phenolic acids
1187	2.06E+02	C14H22O	2,4-Di-Tert-Butylphenol*	Phenolic acids
1188	1.12E+02	C4H4N2O2	Uracil	Nucleotides and derivatives
1189	2.76E+02	C14H16N2O4	Cyclo(Phe-Glu)	Amino acids and derivatives
1190	3.33E+02	C16H19N3O5	γ-Glu-Trp	Amino acids and derivatives
1191	1.88E+02	C9H16O4	Eucommiol	Others
1192	1.89E+02	C7H15N3O3	L-Homocitrulline	Amino acids and derivatives
1193	1.62E+02	C9H6O3	8-Hydroxycoumarin	Lignans and Coumarins
1194	1.38E+02	C7H6O3	Protocatechualdehyde	Others
1195	3.54E+02	C16H18O9	Noreugenin-7-O-glucoside*	Others
1196	1.82E+02	C9H10O4	(S)-2-Hydroxy-3-(4-Hydroxyphenyl)Propanoic Acid	Phenolic acids

1197	5.16E+02	C25H24O12	1,3-O-Dicaffeoylquinic Acid (Cynarin)	Phenolic acids
1198	1.94E+02	C11H14O3	Dehydrololiolide	Terpenoids
1199	3.00E+02	C12H12O9	Vnilloyltartaric acid	Phenolic acids
1200	5.82E+02	C28H38O13	Lyoniresinol-3-O-glucoside	Lignans and Coumarins
1201	2.78E+02	C15H22N2O3	L-Leucyl-L-phenylalanine	Amino acids and derivatives
1202	5.33E+02	C27H52NO7P	LysoPC 19:2	Lipids
1203	2.60E+02	C6H13O9P	D-Glucose-1-phosphate*	Others
1204	1.54E+02	C7H6O4	2,5-Dihydroxybenzoic acid; Gentisic Acid*	Phenolic acids
1205	2.36E+02	C10H20O6	Butyl Beta-D-Fructopyranoside	Others
1206	3.74E+02	C16H22O10	1-O-(3,4,5-Trimethoxybenzoyl)-B-D-Glucopyranoside	Phenolic acids
1207	4.56E+02	C30H48O3	3-Hydroxyurs-12-en-28-oic acid (Ursolic acid)*	Terpenoids
1208	4.48E+02	C21H36O10	Geranyl 6-O-xylopyranosyl-glucopyranoside*	Others
1209	3.16E+02	C14H20O8	3,4-dihydroxyphenylethanol-β-D-glucopyranoside	Phenolic acids
1210	1.49E+02	C5H11NO2S	L-Methionine	Amino acids and derivatives
1211	1.84E+02	C9H12O4	Vanylglycol	Others
1212	4.52E+02	C21H24O11	Epicatechin glucoside	Flavonoids
1213	2.24E+02	C13H20O3	Vomifoliol (Blumenol A)	Terpenoids
1214	5.64E+02	C26H28O14	Lucidin primeveroside	Quinones
1215	1.68E+02	C8H8O4	Vanillic acid	Phenolic acids
1216	1.63E+02	C5H9NO5	4-Hydroxy-L-glutamic acid	Amino acids and derivatives
1217	5.26E+02	C24H30O13	isatioxyneolignoside A	Lignans and Coumarins
1218	4.96E+02	C25H28N4O7	Trp-Glu-Tyr	Amino acids and derivatives
1219	4.75E+02	C24H29NO9	N-Feruloyltyramine 4-glucoside	Alkaloids
1220	1.62E+02	C10H10O2	5-Hydroxy-1-tetralone	Others
1221	4.58E+02	C22H18O11	Gallocatechin 3-O-gallate	Flavonoids
1222	4.87E+02	C27H29N5O4	Trp-Pro-Trp	Amino acids and derivatives
1223	4.56E+02	C17H21N4O9P	Flavin Single Nucleotide(FMN)	Nucleotides and derivatives
1224	5.55E+02	C26H54NO9P	2-(2,3-dihydroxypropoxy)-3-(((2-(dimethylamino)ethoxy)(hydroxy)phosphoryl)oxy)propyl palmitate*	Others
1225	2.07E+02	C7H13NO4S	S-(2-Carboxypropyl)cysteine	Amino acids and derivatives
1226	2.78E+02	C18H30O2	Octadeca-11E,13E,15Z-trienoic acid	Lipids
1227	3.68E+02	C17H20O9	1-O-Feruloylquinic acid	Phenolic acids

1228	3.68E+02	C17H20O9	Murrayacarpin A glucoside	Lignans and Coumarins
1229	2.44E+02	C10H16N2O3S	Biotin	Others
1230	3.08E+02	C20H36O2	Eicosadienoic acid	Lipids
1231	3.94E+02	C19H22O9	6-Hydroxyrumicin-8-O-D-glucoside	Quinones
1232	3.07E+02	C10H17N3O6S	Glutathione reduced form	Amino acids and derivatives
1233	1.76E+02	C6H8O6	D-Glucurono-6,3-lactone	Others
1234	3.10E+02	C18H30O4	9-Hydroperoxy-10E,12,15Z-octadecatrienoic acid	Lipids
1235	3.30E+02	C17H14O7	Quercetin-3,4'-Dimethyl Ether	Flavonoids
1236	2.27E+02	C9H13N3O4	2'-Deoxycytidine	Nucleotides and derivatives
1237	2.88E+02	C10H20N6O4	Asn-Arg	Amino acids and derivatives
1238	3.42E+02	C12H22O11	Isomaltulose*	Others
1239	3.35E+02	C15H29NO7	N-(1-Deoxy-1-fructosyl)Aminononanoic acid	Alkaloids
1240	2.30E+02	C14H14O3	Demethylsuberosin	Lignans and Coumarins
1241	1.25E+02	C6H11N3	1-Methylhistamine	Alkaloids
1242	4.06E+02	C17H26O11	Morroniside	Terpenoids
1243	4.36E+02	C21H24O10	Phloretin-2'-O-glucoside (Phlorizin)	Flavonoids
1244	3.36E+02	C20H32O4	3,9-Dihydroxy-13(14)-labden-16,15-olide	Terpenoids
1245	2.59E+02	C6H14NO8P	D-Glucosamine 1-phosphate	Others
1246	2.02E+02	C9H18N2O3	2,6-diamino-7-methyl-5-oxooctanoic acid	Amino acids and derivatives
1247	1.78E+02	C6H10O6	D-Glucono-1,5-lactone*	Others
1248	2.36E+02	C12H12O5	5,6,7-Trimethoxycoumarin*	Lignans and Coumarins
1249	1.90E+02	C11H14N2O	5-Methoxytryptamine	Alkaloids
1250	5.68E+02	C23H36O16	6'-O-α-D-galactosylshanzhiside methyl ester	Terpenoids
1251	1.22E+02	C7H6O2	3-hydroxybenzaldehyde	Phenolic acids
1252	5.05E+02	C25H48NO7P	LysoPE 20:2(2n isomer)	Lipids
1253	4.65E+02	C21H21O12+	Delphinidin-3-O-galactoside	Flavonoids
1254	4.04E+02	C17H24O11	8-Epikingiside*	Terpenoids
1255	3.88E+02	C19H32O8	Sammangaoside A	Others
1256	6.24E+02	C28H32O16	Tamarixetin-3-O-rutinoside	Flavonoids
1257	3.14E+02	C14H18O8	Mandelic acid-β-glucoside	Organic acids
1258	4.92E+02	C23H24O12	Tricin-7-O-Glucoside	Flavonoids
1259	2.81E+02	C11H15N5O4	2'-O-Methyladenosine	Nucleotides and derivatives
1260	4.81E+02	C23H48NO7P	LysoPE 18:0	Lipids
1261	4.78E+02	C22H22O12	Isorhamnetin-7-O-glucoside (Brassicin)*	Flavonoids
1262	4.77E+02	C23H44NO7P	LysoPE 18:2(2n isomer)	Lipids

1263	4.20E+02	C6H15O15P3	d-Myo-inositol-1,4,5-triphosphate	Organic acids
1264	1.31E+02	C6H13NO2	6-Aminocaproic acid	Organic acids
1265	1.79E+02	C6H13NO5	D-Glucosamine	Others
1266	3.46E+02	C16H26O8	Jasminoside G	Terpenoids
1267	2.71E+02	C12H17NO6	3-pyridine-methanol-O-β-D-glucopyranosyl	Alkaloids
1268	3.00E+02	C13H16O8	Glucosyloxybenzoic acid	Phenolic acids
1269	3.10E+02	C14H30O5S	2-Dodecoxyethyl Hydrogen Sulfate	Phenolic acids
1270	3.12E+02	C18H20N2O3	L-Phenylalanyl-L-phenylalanine	Amino acids and derivatives
1271	2.86E+02	C15H10O6	Isoscutellarein*	Flavonoids
1272	2.96E+02	C14H20N2O3S	Met-Phe	Amino acids and derivatives
1273	4.22E+02	C12H23O14P	Sucrose-6-phosphate	Others
1274	4.86E+02	C30H46O5	wilforic acid E	Terpenoids
1275	3.72E+02	C20H20O7	3',4',5',5,7-Pentamethoxyflavone*	Flavonoids
1276	1.80E+02	C9H8O4	5-Acetylsalicylic acid	Phenolic acids
1277	1.07E+02	C7H9N	2,6-Dimethylpyridine	Alkaloids
1278	1.29E+02	C9H7N	Isoquinoline	Alkaloids
1279	4.24E+02	C19H36O10	Octyl 6-O-Alpha-L-Arabinopyranosyl-Beta-D-Glucopyranoside	Others
1280	2.67E+02	C10H13N5O4	9-Alpha-Ribofuranosyladenine*	Nucleotides and derivatives
1281	1.04E+02	C5H14NO+	Choline	Alkaloids
1282	6.78E+02	C31H34O17	1,5-O-dicaffeoyl-3-O-glucoside-quinic acid*	Phenolic acids
1283	2.80E+02	C13H12O7	p-Coumaroylmalic acid	Phenolic acids
1284	2.86E+02	C17H34O3	3-Hydroxy-palmitic acid methyl ester	Lipids
1285	4.04E+02	C17H24O11	6α-Hydroxygeniposide	Terpenoids
1286	1.66E+02	C9H10O3	DL-3-Phenyllactic acid*	Organic acids
1287	1.52E+02	C5H4N4O2	Xanthine	Nucleotides and derivatives
1288	2.78E+02	C14H18N2O4	Phe-Hyp	Amino acids and derivatives
1289	3.36E+02	C20H32O4	8,15-Dihydroxy-5,9,11,13-eicosatetraenoic acid	Lipids
1290	3.52E+02	C16H16O9	Esculetin-7-O-quinic acid	Lignans and Coumarins
1291	1.64E+02	C9H8O3	3',4'-(Methylenedioxy)acetophenone	Others
1292	1.23E+02	C6H5NO2	2-Picolinic acid	Organic acids
1293	3.02E+02	C12H14O9	Gallic acid-1-O-xyloside	Phenolic acids
1294	3.56E+02	C16H20O9	gentiananoside B	Terpenoids
1295	3.60E+02	C12H20N6O7	Asn-Asn-Asn	Amino acids and derivatives
1296	2.92E+02	C18H28O3	12-Oxo-phytodienoic acid	Lipids
1297	3.88E+02	C17H24O10	Geniposide	Terpenoids

1298	3.84E+02	C22H28N2O4	Corynoxine*	Alkaloids
1299	3.84E+02	C22H28N2O4	Corynoxine B*	Alkaloids
1300	2.94E+02	C18H30O3	9-Oxo-10E,12Z-octadecadienoic acid	Lipids
1301	4.48E+02	C21H20O11	Luteolin-4'-O-glucoside*	Flavonoids
1302	2.02E+02	C9H18N2O3	(S)-4-amino-5-(butylamino)-5-oxopentanoic acid	Amino acids and derivatives
1303	2.46E+02	C6H15O8P	sn-glycero-3-phospho-(1'-sn-glycerol)	Others
1304	4.70E+02	C30H46O4	2,3-Dihydroxy-5(6),12(13)-diene-ursolic acid*	Terpenoids
1305	3.26E+02	C15H18O8	p-Coumaric acid-4-O-glucoside	Phenolic acids
1306	1.35E+02	C8H9NO	N-benzylformamide	Alkaloids
1307	4.98E+02	C25H22O11	Dicaffeoylshikimic acid	Phenolic acids
1308	4.04E+02	C17H24O11	10-acetylmonomelittoside	Phenolic acids
1309	1.18E+03	C60H95NO22	Mussaendoside O	Terpenoids
1310	2.93E+02	C15H23N3O3	Lys-Phe	Amino acids and derivatives
1311	2.93E+02	C12H23NO7	N-(1-Deoxy-1-fructosyl)Leucine	Amino acids and derivatives
1312	3.70E+02	C17H22O9	Sinapaldehyde-4-O-Glucoside	Phenolic acids
1313	3.28E+02	C15H20O8	Cryptamygin B	Phenolic acids
1314	3.17E+02	C17H23N3O3	Ile-Trp	Amino acids and derivatives
1315	3.76E+02	C16H24O10	6β-diydrocormic acid	Others
1316	5.18E+02	C24H38O12	(6S,7E,9R)-6,9-Dihydroxy-4,7-megastigmadin-3 -one-9-O-xylosyl(1-6)glucoside	Others
1317	1.66E+02	C10H14O2	Dihydrohinokitiol	Terpenoids
1318	7.43E+02	C21H28N7O17P 3	NADP (Nicotinamide adenine dinucleotide phosphate)	Nucleotides and derivatives
1319	1.22E+02	C7H10N2	2,3,5-Trimethylpyrazine	Alkaloids
1320	1.37E+02	C7H7NO2	Nicotinic Acid Methyl Ester(Methyl Nicotinate)	Alkaloids
1321	3.54E+02	C21H38O4	1-Linoleoylglycerol*	Lipids
1322	3.28E+02	C19H20O5	Uvafzelic acid	Others
1323	3.28E+02	C22H32O2	Cis-4,7,10,13,16,19-Docosahexaenoic Acid	Lipids
1324	4.38E+02	C30H46O2	3,11-dioxo-β-oleorene	Terpenoids
1325	5.07E+02	C10H16N5O13P 3	ATP; Adenosine 5'-Triphosphate	Nucleotides and derivatives
1326	2.80E+02	C13H16N2O5	L-Aspartyl-L-Phenylalanine	Amino acids and derivatives
1327	1.67E+02	C7H5NO4	Quinolinic Acid	Alkaloids
1328	1.12E+02	C6H8O2	2,4-Hexadienoic acid	Organic acids
1329	8.86E+02	C45H74O17	Sarsasapogenin-3-O-glucosyl(1→2)[rhamnosyl(1→4)]glucoside (Asparanin B)	Steroids
1330	1.74E+02	C8H14O4	Suberic Acid	Organic acids
1331	6.14E+02	C32H38O12	Buddlenol F	Lignans and Coumarins

1332	4.80E+02	C21H20O13	Myricetin-3-O-galactoside*	Flavonoids
1333	2.08E+02	C10H8O5	Fraxetin (7,8-Dihydroxy-6-methoxycoumarin)	Lignans and Coumarins
1334	4.58E+02	C30H50O3	Olean-12-ene-3,22,23-triol (Soyasapogenol B)	Terpenoids
1335	3.40E+02	C6H14O12P2	D-Fructose-1,6-biphosphate	Others
1336	2.67E+02	C10H13N5O4	9-Arabinosyladenine*	Nucleotides and derivatives
1337	9.56E+02	C48H76O19	3-O-Glucosyl(1→4)rhamnosyl quinovic acid-28-O-glucosyl ester*	Terpenoids
1338	5.04E+02	C30H48O6	2,3,7,19-Tetrahydroxyurs-12-en-28-oic acid (Roxburic Acid)*	Terpenoids
1339	1.82E+02	C6H14O6	D-Mannitol*	Others
1340	1.04E+02	C4H8O3	3-Hydroxybutyric acid	Organic acids
1341	3.44E+02	C20H24O5	Anhydrosecoisolariciresinol (AHS)*	Lignans and Coumarins
1342	2.45E+02	C9H15N3O5	Asn-Hyp	Amino acids and derivatives
1343	5.18E+02	C22H30O14	6'-O-Feruloyl-D-sucrose	Phenolic acids
1344	4.48E+02	C21H20O11	Luteolin-7-O-glucoside (Cynaroside)*	Flavonoids
1345	3.28E+02	C15H20O8	(E)-caffeyl alcohol 4-O-β-D-glucopyranoside	Phenolic acids
1346	1.17E+02	C4H7NO3	N-Acetyl-L-glycine	Amino acids and derivatives
1347	5.05E+02	C25H48NO7P	1-Linoleoyl-2-Lysophosphatidic Acid Monobutylamine Ester	Others
1348	3.12E+02	C18H32O4	13-Hydroperoxy-9Z,11E-octadecadienoic acid*	Lipids
1349	3.12E+02	C18H32O4	7S,8S-DiHODE; (9Z,12Z)-(7S,8S)-Dihydroxyoctadeca-9,12-dienoic acid*	Lipids
1350	4.06E+02	C17H26O11	6-β-Hydroxyloganin	Terpenoids
1351	1.26E+02	C7H10O2	2-Methylcyclohexane-1,3-dione	Others
1352	4.46E+02	C24H30O8	Isodunnianin	Lignans and Coumarins
1353	4.37E+02	C20H40NO7P	LysoPE 15:1*	Lipids
1354	1.50E+02	C5H10O5	D-Arabinose*	Others
1355	4.47E+02	C23H29NO8	3'-Glucosyl-6,7-dihydroxy-N-methyl-benzyltetrahydroisoquinoline	Alkaloids
1356	4.62E+02	C22H22O11	Chrysoeriol-5-O-glucoside	Flavonoids
1357	2.86E+02	C20H30O	Ferruginol*	Terpenoids
1358	5.00E+02	C22H28O13	1,2,4-Trihydroxynaphthalene-1,4-di-glucoside (Lawsoniaside)	Others
1359	4.68E+02	C22H28O11	Cimifugin-7-O-glucoside	Others
1360	1.48E+02	C10H12O	4-Methylphenylacetone	Others
1361	3.00E+02	C16H12O6	1,6-dihydroxy-2,5-dimethoxy-9,10-anthraquinone	Quinones

1362	4.56E+02	C30H48O3	Rubione D*	Terpenoids
1363	4.32E+02	C18H24O12	Licoagroside B	Others
1364	4.64E+02	C21H20O12	Quercetin-7-O-glucoside	Flavonoids
1365	1.70E+02	C3H7O6P	Dihydroxyacetone phosphate	Others
1366	1.32E+02	C4H8N2O3	L-Asparagine	Amino acids and derivatives
1367	5.00E+02	C22H28O13	5-O-p-Coumaroylquinic acid O-glucoside	Phenolic acids
1368	4.40E+02	C30H48O2	3-Hydroxylup-20(29)-en-28-al (Betulinaldehyde)	Terpenoids
1369	3.10E+02	C16H24NO5+	Sinapine	Alkaloids
1370	1.16E+02	C5H8O3	4-Oxopentanoic Acid	Organic acids
1371	5.38E+02	C22H34O15	6'-O-β-glucopyranosyl secologanol	Terpenoids
1372	4.79E+02	C23H46NO7P	LysoPE 18:1(2n isomer)*	Lipids
1373	3.74E+02	C16H22O10	Swertiamarin	Terpenoids
1374	5.10E+02	C24H30O12	8-O-(p-Coumaroyl)Harpagide	Terpenoids
1375	3.54E+02	C12H18O12	Citric acid glucoside	Organic acids
1376	1.46E+02	C5H6O5	2-Methyl-3-oxosuccinic acid	Organic acids
1377	4.62E+02	C21H18O12	Kaempferol-3-O-glucuronide	Flavonoids
1378	2.78E+02	C16H22O4	Butyl isobutyl phthalate*	Phenolic acids
1379	4.62E+02	C21H18O12	Demethylwedelolactone-3-O-glucoside	Lignans and Coumarins
1380	2.30E+02	C13H14N2O2	(1S,3S)-1-Methyl-1,2,3,4-tetrahydro-β-carbolin e-3-carboxylic acid	Alkaloids
1381	2.70E+02	C15H10O5	2,5-dihydroxy-1-methoxy-anthraquinone	Quinones
1382	2.21E+02	C11H11NO4	Methyl dioxindole-3-acetate	Alkaloids
1383	5.22E+02	C24H26O13	Centaurein	Flavonoids
1384	3.00E+02	C13H16O8	Salicylic acid-2-O-glucoside	Phenolic acids
1385	8.62E+02	C42H38O20	Sennoside B	Quinones
1386	3.76E+02	C17H20N4O6	Riboflavin (Vitamin B2)	Others
1387	4.91E+02	C24H46NO7P	LysoPC 16:2	Lipids
1388	4.16E+02	C22H24O8	Ovafolinin E	Lignans and Coumarins
1389	4.36E+02	C21H24O10	Dihydrocharcone-4'-O-glucoside	Flavonoids
1390	2.74E+02	C15H14O5	Epiafzelechin	Flavonoids
1391	2.22E+02	C11H14N2O3	L-Glycyl-L-phenylalanine*	Amino acids and derivatives
1392	4.37E+02	C20H40NO7P	LysoPE 15:1(2n isomer)*	Lipids
1393	1.32E+02	C5H8O4	Dimethylmalonic acid*	Organic acids
1394	2.38E+02	C11H10O6	Benzoylmalic acid	Phenolic acids
1395	6.80E+02	C36H56O12	2,3,19-Trihydroxyurs-12-en-23,28-dioic acid-28-O-glucoside	Terpenoids
1396	2.42E+02	C15H30O2	13-methylmyristic acid	Lipids
1397	1.52E+02	C4H4N6O	8-Azaguanine	Nucleotides and derivatives

1398	2.87E+02	C12H25N5O3	Leu-Arg	Amino acids and derivatives
1399	6.10E+02	C30H26O14	Gallocatechin-(4α→8)-gallocatechin	Flavonoids
1400	5.00E+02	C22H28O13	3-O-p-Coumaroylquinic acid-O-glucoside	Phenolic acids
1401	1.04E+02	C3H4O4	Malonic acid	Organic acids
1402	1.74E+02	C10H10N2O	hupcrispatine	Alkaloids
1403	3.44E+02	C15H20O9	Syringaldehyde-4-O-glucoside	Phenolic acids
1404	4.86E+02	C30H46O5	3-Hydroxyurs-12-ene-27,28-dioic acid (Quinovic acid)	Terpenoids
1405	3.59E+02	C16H29N3O6	Asp-Ile-Leu	Amino acids and derivatives
1406	3.76E+02	C16H24O10	D-Threo-guaiacylglycerol-7-O-β-D-glucoside	Phenolic acids
1407	1.52E+02	C9H12O2	3-(4-Hydroxyphenyl)-1-propanol	Phenolic acids
1408	3.42E+02	C16H22O8	Baihuaqianhuoside	Others
1409	1.45E+02	C9H7NO	8-hydroxyquinoline	Alkaloids
1410	5.16E+02	C27H48O9	1-O-Linoleoyl-3-O-galactopyranosyl-L-glycerol	Lipids
1411	3.42E+02	C15H18O9	1-O-Caffeoyl-β-D-glucose*	Phenolic acids
1412	5.96E+02	C26H28O16	Quercetin-3-O-apiosyl(1→2)galactoside	Flavonoids
1413	1.45E+02	C6H11NO3	N-Methyl-Trans-4-Hydroxy-L-Proline	Amino acids and derivatives
1414	2.81E+02	C11H15N5O4	N6-methyladenosine	Nucleotides and derivatives
1415	3.72E+02	C20H20O7	8-Hydroxy-α-conidendrin	Lignans and Coumarins
1416	2.91E+02	C10H17N3O7	Ser-Glu-Gly	Amino acids and derivatives
1417	3.02E+02	C20H30O2	Isopimaric acid*	Terpenoids
1418	5.94E+02	C27H30O15	Luteolin-7-O-neohesperidoside (Lonicerin)*	Flavonoids
1419	5.94E+02	C27H30O15	Luteolin-7-O-rutinoside*	Flavonoids
1420	5.94E+02	C27H30O15	Kaempferol-3-O-glucorhamnoside*	Flavonoids
1421	5.94E+02	C27H30O15	Kaempferol-3-O-glucoside-7-O-rhamnoside*	Flavonoids
1422	4.31E+02	C20H25N5O6	Phe-His-Glu	Amino acids and derivatives
1423	1.20E+02	C8H8O	4-Methylbenzaldehyde*	Others
1424	3.86E+02	C19H30O8	Roseoside	Others
1425	6.78E+02	C31H34O17	Dicaffeoylquinic acid-O-glucoside	Phenolic acids
1426	2.43E+02	C9H13N3O5	Cytarabine	Nucleotides and derivatives
1427	2.78E+02	C11H22N2O4S	Pantetheine	Alkaloids
1428	1.67E+02	C8H9NO3	2-Amino-3-methoxybenzoic acid	Phenolic acids
1429	4.16E+02	C15H28O13	Digalactosylglycerol	Others
1430	1.38E+02	C8H10O2	Tyrosol; 4-Hydroxyphenylethanol	Phenolic acids
1431	5.45E+02	C28H52NO7P	LysoPC 20:3	Lipids
1432	5.46E+02	C27H34N2O10	3α-Dihydrocadambine	Alkaloids

1433	2.40E+02	C14H12N2O2	1-Ethoxycarbonyl-β-Carboline	Alkaloids
1434	1.96E+02	C11H16O3	Loliolide*	Terpenoids
1435	2.66E+02	C14H20NO4+	Caffeoylcholine	Alkaloids
1436	2.22E+02	C11H10O5	Fraxidin (8-Hydroxy-6,7-dimethoxycoumarin)	Lignans and Coumarins
1437	5.32E+02	C25H24O13	Caffeyl fraxin	Lignans and Coumarins
1438	1.37E+02	C7H7NO2	Anthranilic Acid	Phenolic acids
1439	3.62E+02	C16H26O9	1-O-Glucopyranosylamplexine	Terpenoids
1440	1.30E+02	C5H6O4	Itaconic acid	Organic acids
1441	7.70E+02	C41H70O13	Gynoside A	Terpenoids
1442	5.08E+02	C21H32O14	6'-O-α-D-Xylopyranosylloganic acid;Qinjiaoside C	Terpenoids
1443	3.60E+02	C15H20O10	Glucosyringic acid	Phenolic acids
1444	1.46E+02	C6H10O4	2-Acetyl-2-Hydroxybutanoic Acid	Organic acids
1445	2.84E+02	C10H12N4O6	Xanthosine	Nucleotides and derivatives
1446	6.78E+02	C34H30O15	1,3,4-Tri-O-Caffeoyl Quinic Acid	Phenolic acids
1447	1.92E+02	C10H8O4	5,7-Dihydroxy-4-methylcoumarin	Lignans and Coumarins
1448	3.24E+02	C20H24O2N2	Quinine	Alkaloids
1449	2.04E+02	C11H12N2O2	cyclo-(Gly-Phe)	Amino acids and derivatives
1450	1.18E+02	C4H6O4	D-Erythronolactone	Organic acids
1451	4.44E+02	C31H40O2	Menatetrenone (Vitamin K2)	Others
1452	3.88E+02	C19H32O8	Dihydrovomifoliol-O-β-D-glucoside*	Terpenoids
1453	1.16E+02	C4H4O4	Fumaric acid*	Organic acids
1454	1.16E+02	C4H4O4	Maleic acid*	Organic acids
1455	3.72E+02	C19H32O7	Byzantionoside B	Others
1456	1.73E+02	C8H15NO3	N-Acetyl-L-leucine	Amino acids and derivatives
1457	4.56E+02	C30H48O3	3-Hydroxylup-20(29)-en-28-oic acid (Betulinic acid)*	Terpenoids
1458	2.67E+02	C10H13N5O4	Vidarabine*	Nucleotides and derivatives
1459	2.39E+02	C14H25NO2	N-Isobutyl-4,5-epoxy-2E-decaenamide	Alkaloids
1460	3.52E+02	C20H20N2O4	cis-Moschamine*	Alkaloids
1461	2.20E+02	C11H12N2O3	5-Hydroxy-DL-tryptophan(5-HTP)	Amino acids and derivatives
1462	4.62E+02	C22H22O11	Chrysoeriol-7-O-glucoside	Flavonoids
1463	3.24E+02	C9H13N2O9P	Uridine 5'-monophosphate	Nucleotides and derivatives
1464	2.04E+02	C11H12N2O2	1-Methoxy-indole-3-acetamide*	Alkaloids
1465	1.79E+02	C10H13NO2	L-Homophenylalanine	Amino acids and

				derivatives
1466	1.66E+02	C10H14O2	1-(4-Methoxyphenyl)-1-propanol	Phenolic acids
1467	4.52E+02	C21H24O11	Sieboldin*	Flavonoids
1468	3.96E+02	C17H24N4O5S1	Phe-Cys-Gln	Amino acids and derivatives
1469	3.74E+02	C16H22O10	Geniposidic acid*	Terpenoids
1470	5.08E+02	C23H24O13	1-O-Galloyl-2-O-Feruloyl-β-D-glucose	Phenolic acids
1471	2.78E+02	C18H30O2	Octadeca-9,12,15-trienoic acid	Lipids
1472	2.08E+02	C8H16O6	Dambonitol	Others
1473	3.87E+02	C15H25N5O5S1	His-Thr-Met	Amino acids and derivatives
1474	4.78E+02	C20H30O13	Kelampayoside A[3,4,5-Trimethoxyphenol-β-D-apiosyl-(1→6)-β-D-glucoside]	Phenolic acids
1475	1.43E+02	C6H9NO3	Methyl L-pyroglutamate	Alkaloids
1476	4.06E+02	C17H26O11	Dihydromonotropein methyl ester	Terpenoids
1477	2.80E+02	C18H32O2	(9Z,11E)-Octadecadienoic acid*	Lipids
1478	2.80E+02	C18H32O2	Linoleic acid*	Lipids
1479	3.10E+02	C14H14O8	Feruloylmalic acid	Phenolic acids
1480	4.06E+02	C20H22O9	Astringin	Others
1481	1.98E+02	C9H10O5	4-Hydroxy-3-methoxymandelate	Phenolic acids
1482	2.11E+02	C10H13NO4	Methyldopa	Amino acids and derivatives
1483	4.48E+02	C21H36O10	patrinioside	Terpenoids
1484	1.86E+02	C11H22O2	Undecylic Acid	Lipids
1485	6.82E+02	C30H34O18	Isorhamnetin-3-O-(6''-acetylglucosyl)(1→3)-glucoside	Flavonoids
1486	5.04E+02	C18H32O16	Laminaran	Others
1487	3.44E+02	C12H24O11	Maltitol	Others
1488	3.35E+02	C13H25N3O5S1	Thr-Leu-Cys	Amino acids and derivatives
1489	3.64E+02	C20H28O6	Phorbol	Terpenoids
1490	6.68E+02	C31H40O16	4-Hydroxyphenethoxy-8-O-β-D-[6-O-(4-O-β-D-glucopyranosyl)-sinapoyl]glucopyranoside	Phenolic acids
1491	1.50E+02	C8H6O3	Benzoylformic acid	Organic acids
1492	2.06E+02	C13H18O2	Arteannuin A	Terpenoids
1493	1.52E+02	C8H8O3	2-hydroxymethyl benzoic acid	Phenolic acids
1494	1.53E+02	C8H11NO2	Dopamine	Alkaloids
1495	2.26E+02	C11H14O5	Genipin	Terpenoids
1496	3.90E+02	C16H22O11	Monotropein	Terpenoids
1497	3.02E+02	C17H34O4	1-Monomyristin	Lipids
1498	4.75E+02	C24H29NO9	N-(4-O-(Glucosyl)-E-feruloyl)-tyramine	Alkaloids
1499	3.56E+02	C16H20O9	3,4,5-Trihydroxy-6-[[3-(4-methoxyphenyl)oxiran-2-yl]methoxy]oxane-2-carboxylic acid	Phenolic acids

1500	2.88E+02	C19H28O2	Trans-dehydrorosinone	Others
1501	3.86E+02	C21H22O7	7-hydroxy-4-methoxyphenanthrene-2-O-glucoside	Quinones
1502	1.75E+02	C10H9NO2	Indole-3-acetic acid (IAA)	Alkaloids
1503	3.44E+02	C20H24O5	3,4-Divanillyltetrahydrofuran*	Phenolic acids
1504	4.48E+02	C21H20O11	Kaempferol-3-O-glucoside (Astragalin)*	Flavonoids
1505	3.00E+02	C15H8O7	Pseudopurpurin	Quinones
1506	1.74E+02	C7H10O5	2-Methyl-3-oxoadipic acid*	Organic acids
1507	1.74E+02	C7H10O5	2-Oxoheptanedionic acid*	Organic acids
1508	3.58E+02	C16H22O9	Dehydroxysecologanic acid	Terpenoids
1509	4.04E+02	C20H20O9	Gardenin C	Flavonoids
1510	4.72E+02	C21H28O12	4-p-Cumaroyl-rhamnosyl-(1→6)-D-glucose	Phenolic acids
1511	1.62E+02	C8H6N2O2	Benzoyleneurea	Alkaloids
1512	4.72E+02	C30H48O4	3,23-Dihydroxyolean-12-en-28-oic acid (Hederagenin)	Terpenoids
1513	3.94E+02	C22H34O6	Trijugin A	Terpenoids
1514	3.42E+02	C12H22O11	D-Trehalose*	Others
1515	1.50E+02	C9H10O2	4-Allylcatechol	Phenolic acids
1516	2.04E+02	C11H12N2O2	DL-Tryptophan*	Amino acids and derivatives
1517	1.25E+02	C6H7NO2	N-Ethylmaleimide (NEM)	Amino acids and derivatives
1518	1.68E+02	C8H8O4	Homogentisic acid*	Phenolic acids
1519	2.26E+02	C14H26O2	Myristoleic acid	Lipids
1520	2.94E+02	C18H30O3	9-Oxooctadeca-10,12-Dienoic Acid	Lipids
1521	6.05E+02	C27H25O16+	Pelargonidin-3-O-(3'',6''-O-dimalonylglucoside)	Flavonoids
1522	4.32E+02	C16H28N6O8	Glu-Arg-Glu	Amino acids and derivatives
1523	5.38E+02	C24H26O14	Limocitrol 3-Glucoside	Flavonoids
1524	3.60E+02	C16H24O9	Ixoroside	Terpenoids
1525	5.04E+02	C24H40O11	Foliasalacioside B1	Terpenoids
1526	5.04E+02	C24H40O11	Guettardionoside	Terpenoids
1527	4.86E+02	C30H46O5	2-Carboxy-3-hydroxy-A(1)-norlupan-20(29)-en-28-oic acid (Ceanothic acid)	Terpenoids
1528	3.52E+02	C16H16O9	4-Methylumbelliferyl glucuronide	Lignans and Coumarins
1529	4.04E+02	C17H24O11	8-epi-kingiside*	Terpenoids
1530	5.04E+02	C30H48O6	2,3,19,23-Tetrahydroxyolean-12-en-28-oic acid (Arjungenin)*	Terpenoids
1531	4.60E+02	C26H36O7	Vibsanin J	Terpenoids
1532	1.64E+02	C6H12O5	Rhamnose*	Others
1533	6.78E+02	C34H30O15	3,4,5-Tricaffeoylquinic acid	Phenolic acids
1534	1.74E+02	C8H18N2O2	N(6),N(6)-Dimethyl-L-lysine	Amino acids and derivatives

1535	2.46E+02	C14H14O4	Decursinol	Lignans and Coumarins
1536	4.65E+02	C24H27N5O5	His-Tyr-Phe	Amino acids and derivatives
1537	1.60E+02	C10H12N2	Tryptamine	Alkaloids
1538	1.68E+02	C8H8O4	Protocatechuic Acid Methyl Ester	Phenolic acids
1539	1.45E+02	C7H19N3	Spermidine	Alkaloids
1540	3.81E+02	C18H40NO5P	Dihydrosphingosine-1-Phosphate	Lipids
1541	1.87E+02	C11H9NO2	3,5-Dihydro-2H-Furo[3,2-C]Quinolin-4-One*	Alkaloids
1542	1.87E+02	C11H9NO2	3-Indoleacrylic acid*	Alkaloids
1543	3.18E+02	C15H18N4O4	His-Tyr	Amino acids and derivatives
1544	1.22E+02	C7H6O2	Benzoic acid	Phenolic acids
1545	5.20E+02	C26H32O11	Pinoresinol-4-O-glucoside	Lignans and Coumarins
1546	4.88E+02	C24H24O11	2-Caffeoyl-6-(4-hydroxycinnamoyl)glucoside	Phenolic acids
1547	1.93E+02	C10H11NO3	N-(2-Methylbenzoyl)glycine	Amino acids and derivatives
1548	3.48E+02	C16H28O8	Laminamplexoside C	Others
1549	4.07E+02	C20H29N3O6	Leu-Glu-Phe	Amino acids and derivatives
1550	4.32E+02	C18H24O12	Asperulosidic acid	Terpenoids
1551	3.68E+02	C17H20O9	Phellodenol H	Lignans and Coumarins
1552	1.68E+02	C8H12N2O2	4-(Aminomethyl)-5-(hydroxymethyl)-2-methylpyridin-3-ol	Alkaloids
1553	4.03E+02	C20H19O9+	Pelargonidin-3-O-arabinoside	Flavonoids
1554	3.01E+02	C16H19N3O3	Pro-Trp	Amino acids and derivatives
1555	3.70E+02	C14H22N6O6	Asn-Thr-His	Amino acids and derivatives
1556	6.11E+02	C27H31O16+	Cyanidin-3,5-O-diglucoside (Cyanin)	Flavonoids
1557	4.66E+02	C21H22O12	Protocatechuic acid 1-O-(Glucosylvanilloyl)	Phenolic acids
1558	5.02E+02	C24H38O11	Urenalobaside C	Terpenoids
1559	1.96E+02	C11H16O3	Isololiolide	Terpenoids
1560	2.25E+02	C12H11N5	6-Benzylaminopurine	Nucleotides and derivatives
1561	3.06E+02	C15H14O7	Epigallocatechin	Flavonoids
1562	5.94E+02	C27H30O15	Vitexin-2''-O-galactoside	Flavonoids
1563	4.88E+02	C24H24O11	1,4,8-Trihydroxynaphthalene-1-O-[6'-O-(3''-methoxy-5''-hydryoxybenzoyl)]glucoside	Quinones
1564	1.66E+02	C9H10O3	3-(4-Hydroxyphenyl)-propionic acid*	Phenolic acids
1565	4.04E+02	C17H24O11	10-hydroxymajoroside	Phenolic acids
1566	6.40E+02	C29H36O16	Orobanchoside	Phenolic acids

1567	1.33E+02	C8H7NO	Oxindole	Alkaloids
1568	1.47E+02	C5H9NO4	L-Glutamic acid	Amino acids and derivatives
1569	3.02E+02	C20H30O2	Kaurenoic Acid*	Terpenoids
1570	5.44E+02	C27H32N2O10	Cadambine	Alkaloids
1571	5.36E+02	C25H28O13	5,4'-Dihydroxy-3,6,7,3'-tetramethoxyflavone-4'-O-glucoside	Flavonoids
1572	5.81E+02	C28H56NO9P	2-(2,3-dihydroxypropoxy)-3-(((2-(dimethylamino)ethoxy)(hydroxy)phosphoryl)oxy)propan-2-yl (Z)-14-Octadecenoic Acid	Others
1573	3.23E+02	C9H14N3O8P	Cytidine 5'-monophosphate(Cytidylic acid)	Nucleotides and derivatives
1574	4.78E+02	C22H22O12	Nepetin-7-O-glucoside(Nepitrin)*	Flavonoids
1575	4.25E+02	C19H40NO7P	LysoPE 14:0(2n isomer)*	Lipids
1576	1.28E+02	C6H8O3	4-Hydroxy-2,5-dimethyl-3(2H)furanone	Others
1577	3.03E+02	C16H21N3O3	Val-Trp	Amino acids and derivatives
1578	5.83E+02	C34H37N3O6	N',N'',N'''-p-Coumaroyl-cinnamoyl-caffeoyl spermidine	Alkaloids
1579	2.20E+02	C15H24O	Nootkatol	Terpenoids
1580	4.78E+02	C22H22O12	1-O-Galloyl-2-O-p-Coumaroyl-β-D-glucose	Phenolic acids
1581	1.64E+02	C9H8O3	3-Hydroxycinnamic Acid*	Phenolic acids
1582	5.62E+02	C27H34N2O11	3β-Isodihydrocadambine 4-oxide	Alkaloids
1583	6.24E+02	C28H32O16	Isorhamnetin-3-O-neohesperidoside	Flavonoids
1584	2.67E+02	C9H17NO6S	S-Ribosyl-L-homocysteine	Amino acids and derivatives
1585	7.70E+02	C45H70O10	Monogalactosyldiacylglycerol	Lipids
1586	3.54E+02	C21H38O4	2-Linoleoylglycerol*	Lipids
1587	3.60E+02	C18H16O8	Rosmarinic acid	Phenolic acids
1588	4.80E+02	C23H28O11	Astraflavonoid C	Flavonoids
1589	4.86E+02	C30H46O5	3-Hydroxyolean-12-ene-28,29-dioic acid (Serratagenic Acid)	Terpenoids
1590	2.07E+02	C11H13NO3	N-Acetyl-D-phenylalanine	Amino acids and derivatives
1591	1.98E+02	C9H10O5	Syringic acid	Phenolic acids
1592	5.22E+02	C22H34O14	Plantarenaloside glucoside	Terpenoids
1593	4.70E+02	C30H46O4	2,3-Dihydroxylup-20(29)-en-28-oic acid	Terpenoids
1594	3.42E+02	C20H22O5	Myricatomentogenin	Others
1595	1.15E+02	C5H9NO2	1-Aminocyclobutanecarboxylic acid	Amino acids and derivatives
1596	4.51E+02	C21H42NO7P	LysoPE 16:1*	Lipids
1597	6.82E+02	C32H42O16	Pinoresinol-4,4'-O-diglucoside	Lignans and Coumarins
1598	2.78E+02	C16H22O4	6β-Hydroxy-8α-methoxyeremophila-1(10),7(11	Terpenoids

			)-dien-12,8β-olide	
1599	1.65E+02	C8H7NO3	2-(Formylamino)benzoic acid	Phenolic acids
1600	1.94E+02	C10H10O4	3ξ-(1ξ-Hydroxyethyl)-7-hydroxy-1-isobenzofuranone	Others
1601	8.90E+02	C48H75NO14	Mussaendoside D	Terpenoids
1602	2.17E+02	C9H15NO5	N-malonylleucine	Amino acids and derivatives
1603	1.25E+02	C5H7N3O	5-Methylcytosine	Nucleotides and derivatives
1604	3.88E+02	C19H32O8	Bridelionoside F	Terpenoids
1605	6.11E+02	C27H31O16+	Delphinidin-3-O-rutinoside	Flavonoids
1606	1.94E+02	C10H14N2O2	Cyclo(Pro-Pro)	Amino acids and derivatives
1607	1.25E+02	C2H8NO3P	2-Aminoethylphosphonate	Others
1608	5.50E+02	C23H34O15	Genameside C	Terpenoids
1609	4.49E+02	C21H21O11+	Petunidin-3-O-arabinoside	Flavonoids
1610	3.02E+02	C20H30O2	Levopimaric acid*	Terpenoids
1611	4.48E+02	C19H28O12	8-O-Acetyl shanzhiside methyl ester	Terpenoids
1612	5.04E+02	C24H40O11	Cuneataside E	Others
1613	4.49E+02	C21H21O11+	Cyanidin-3-O-galactoside*	Flavonoids
1614	4.64E+02	C22H24O11	Hesperetin-7-O-glucoside	Flavonoids
1615	4.18E+02	C22H26O8	Olivil Monoacetate	Lignans and Coumarins
1616	3.86E+02	C22H26O6	epieudesmin	Lignans and Coumarins
1617	5.34E+02	C26H30O12	Noricariside	Flavonoids
1618	2.04E+02	C12H12O3	2,2-dimethylchromene-6-carboxylic acid	Others
1619	4.33E+02	C21H21O10+	Pelargonidin-3-O-glucoside	Flavonoids
1620	3.30E+02	C15H22O8	Bartsioside	Terpenoids
1621	1.75E+02	C10H9NO2	7-Amino-4-methylcoumarin	Lignans and Coumarins
1622	2.90E+02	C14H10O7	4-(3,4,5-Trihydroxybenzoxy)benzoic acid	Phenolic acids
1623	1.64E+02	C6H12O5	D-Fucose*	Others
1624	1.64E+02	C6H12O5	L-Fucose*	Others
1625	4.56E+02	C23H28N4O6	Gln-Tyr-Phe	Amino acids and derivatives
1626	6.50E+02	C36H58O10	Pedunculoside	Terpenoids
1627	4.48E+02	C21H20O11	Cimicifugic acid A	Phenolic acids
1628	3.68E+02	C16H16O10	Scopoletin-7-O-glucuronide	Lignans and Coumarins
1629	1.24E+02	C7H8O2	Salicyl Alcohol	Phenolic acids
1630	3.52E+02	C20H20N2O4	N-Feruloylserotonin*	Alkaloids
1631	6.50E+02	C31H38O15	xylosyl Phellamurin	Flavonoids
1632	3.43E+02	C14H17NO9	2-O-Glucosyl-7-hydroxy-1,4(2H)-benzoxazin-3-	Alkaloids

			one (DHBOA glucoside)	
1633	1.81E+02	C9H11NO3	N-(2-Hydroxy-4-methoxyphenyl)acetamide	Alkaloids
1634	3.54E+02	C16H18O9	4-O-acetyl-3-O-caffeoyl-2-C-methyl-D-erythronate*	Others
1635	3.90E+02	C16H22O11	Secologanoside	Terpenoids
1636	2.24E+02	C12H16O4	(2E)-3-(1-Hydroxy-2,6,6-trimethyl-4-oxo-2-cyclohexen-1-yl)-2-propenoic acid	Others
1637	4.72E+02	C24H24O10	4-O-(6''-P-coumaroyl-β-D-glucopyranosyl)-P-coumaric acid	Others
1638	1.45E+02	C6H11NO3	N-Hydroxypipecolic acid	Alkaloids
1639	4.03E+02	C18H21N5O6	N-(p-hydroxybenzyl) adenosine	Nucleotides and derivatives
1640	2.26E+02	C9H10N2O5	2,2'-Cyclouridine	Alkaloids
1641	6.06E+02	C35H42O9	1-Deacetylnimbolinin A	Terpenoids
1642	1.78E+02	C9H6O4	3,7-Dihydroxychromen-4-one	Others
1643	4.88E+02	C18H32O15	3'-Fucosyllactose	Others
1644	5.20E+02	C22H32O14	Genipin-1-O-(2''-O-apiosyl)glucoside	Terpenoids
1645	1.68E+02	C11H8N2	Norharmane; Beta-Carboline	Alkaloids
1646	3.70E+02	C21H26N2O4	Isorhynchophyllic acid	Alkaloids
1647	1.24E+02	C7H8O2	3-Methylcatechol	Phenolic acids
1648	4.80E+02	C21H20O13	Myricetin-3-O-β-D-glucoside*	Flavonoids
1649	1.20E+03	C60H95NO23	Mussaendoside P	Terpenoids
1650	3.12E+02	C18H32O4	9-Hydroxy-13-oxo-10-octadecenoic Acid	Lipids
1651	1.20E+02	C7H8N2	Benzamidine	Alkaloids
1652	3.58E+02	C16H22O9	10-Deoxygeniposidic acid	Terpenoids
1653	2.10E+02	C11H18N2O2	Cyclo(D-Leu-L-Pro)*	Amino acids and derivatives
1654	3.26E+02	C12H22O10	2-O-alpha-L-Rhamnopyranosyl-D-glucopyranose	Others
1655	3.54E+02	C16H18O9	Isoscopoletin-β-D-glucoside*	Lignans and Coumarins
1656	4.49E+02	C21H21O11+	Cyanidin-3-O-glucoside*	Flavonoids
1657	5.18E+02	C26H30O11	Phellamurin	Flavonoids
1658	4.18E+02	C21H22O9	Liquiritigenin-4'-O-Glucoside (Liquiritin)	Flavonoids
1659	7.36E+02	C39H60O13	Gentrogenin-3-O-glucosyl(1→4)fucoside (Kingianoside B)	Steroids
1660	3.28E+02	C16H24O7	Jasminoside C	Terpenoids
1661	7.56E+02	C33H40O20	Kaempferol-3-O-neohesperidoside-7-O-glucoside*	Flavonoids
1662	7.56E+02	C33H40O20	Kaempferol-3-O-sophoroside-7-O-rhamnoside*	Flavonoids
1663	4.61E+02	C21H31N7O5	Thr-Trp-Arg	Amino acids and derivatives
1664	2.34E+02	C12H14N2O3	5-Methoxy-DL-tryptophan	Amino acids and derivatives

1665	2.16E+02	C11H20O4	Undecanedioic acid	Lipids
1666	4.74E+02	C21H14O13	Trigallic acid	Phenolic acids
1667	1.60E+02	C10H8O2	2,3-Dihydro-1,4-naphthoquinone	Quinones
1668	1.70E+02	C3H7O6P	DL-Glyceraldehyde-3-phosphate	Organic acids
1669	4.18E+02	C20H18O10	Luteolin-8-C-arabinoside	Flavonoids
1670	1.68E+02	C8H8O4	Gallacetophenone	Phenolic acids
1671	4.32E+02	C21H20O10	kwanzoquinone C	Quinones
1672	5.48E+02	C25H40O13	Glucosyl corchoionoside C	Terpenoids
1673	5.18E+02	C24H38O12	pteleifoside C	Others
1674	1.56E+02	C10H8N2	3-Indoleacetonitrile	Alkaloids
1675	6.24E+02	C29H36O15	Isoacteoside	Phenolic acids
1676	3.76E+02	C16H24O10	8-hydroxy-10-hydrosweroside*	Terpenoids
1677	3.76E+02	C16H24O10	Adoxosidic acid*	Terpenoids
1678	3.46E+02	C17H14O8	5,6,3',4'-Tetrahydroxy-3,7-dimethoxyflavone	Flavonoids
1679	5.50E+02	C25H26O14	Luteolin-6,8-di-C-arabinoside	Flavonoids
1680	1.66E+02	C9H10O3	3-(3-Hydroxyphenyl)-propionic acid	Phenolic acids
1681	1.50E+02	C4H6O6	L-Tartaric acid	Organic acids
1682	2.78E+02	C15H22N2O3	Phe-Ile	Amino acids and derivatives
1683	6.25E+02	C31H29O14+	Petunidin-3-O-(6''-O-p-Coumaroyl)glucoside	Flavonoids
1684	1.64E+02	C9H8O3	p-Coumaric acid*	Phenolic acids
1685	5.58E+02	C34H54O6	Chisopanin D	Terpenoids
1686	4.33E+02	C19H23N5O7	Tyr-His-Asp	Amino acids and derivatives
1687	7.08E+02	C38H60O12	Ruscogenin-1-O-xylosyl(1→3)fucoside	Steroids
1688	5.94E+02	C27H30O15	Isovitexin-7-O-glucoside(Saponarin)	Flavonoids
1689	6.08E+02	C28H32O15	Chrysoeriol-7-O-rutinoside	Flavonoids
1690	1.76E+02	C6H8O6	Erythorbic Acid; Isoascorbic Acid	Others
1691	3.12E+02	C18H16O5	5,7,4'-Trimethoxyflavone	Flavonoids
1692	5.80E+02	C26H28O15	Luteolin-7-O-glucoside-5-O-arabinoside	Flavonoids
1693	4.04E+02	C9H14N2O12P2	Uridine 5'-diphosphate	Nucleotides and derivatives
1694	1.66E+02	C6H6N4S	6-Methylmercaptopurine	Nucleotides and derivatives
1695	4.12E+02	C17H24N4O6S1	Gln-Cys-Tyr	Amino acids and derivatives
1696	3.68E+02	C17H20O9	methyl 5-caffeoylquinate*	Phenolic acids
1697	6.66E+02	C24H42O21	Nystose	Others
1698	2.20E+02	C8H16N2O5	Thr-Thr	Amino acids and derivatives
1699	1.04E+03	C54H85NO18	Heinsiagenin A	Terpenoids
1700	1.17E+02	C5H11NO2	5-Aminovaleric acid	Organic acids
1701	4.34E+02	C21H22O10	6-O-Caffeoylarbutin	Phenolic acids
1702	2.84E+02	C14H20O6	2-Phenylethyl-1-O-β-D-glucoside	Phenolic acids

1703	4.75E+02	C24H29NO9	N-Feruloyltyramine 4'-glucoside	Alkaloids
1704	3.54E+02	C21H26N2O3	Yohimbine	Alkaloids
1705	7.31E+01	C2H7N3	1-Methylguanidine	Alkaloids
1706	4.42E+02	C20H26O11	Dimethylasperuloside	Terpenoids
1707	2.21E+02	C8H15NO6	N-Acetyl-D-mannosamine	Others
1708	1.92E+02	C11H12O3	Eugenyl formate	Others
1709	3.54E+02	C16H18O9	1-Caffeoylquinic acid	Phenolic acids
1710	1.93E+02	C10H11NO3	N-Phenylacetylglycine	Amino acids and derivatives
1711	2.70E+02	C13H18O6	Benzyl glucoside	Phenolic acids
1712	2.10E+02	C7H14O7	Sedoheptulose	Others
1713	3.42E+02	C20H22O5	Lignans Machilin F	Lignans and Coumarins
1714	2.26E+02	C11H14O5	Sarracenin	Terpenoids
1715	2.68E+02	C13H16O6	1-Feruloyl-sn-glycerol	Phenolic acids
1716	2.86E+02	C15H10O6	Scutellarein (5,6,7,4'-Tetrahydroxyflavone)	Flavonoids
1717	1.26E+02	C6H6O3	Maltol	Others
1718	3.70E+02	C19H30O7	Reseoside	Terpenoids
1719	2.26E+02	C11H18N2O3	Cyclo(L-Leu-trans-4-hydroxy-L-Pro)	Amino acids and derivatives
1720	3.72E+02	C19H32O7	myrsinionoside A	Terpenoids
1721	3.15E+02	C15H29N3O4	Ala-Ile-Leu	Amino acids and derivatives
1722	5.64E+02	C26H28O14	Isovitexin-2''-O-xyloside	Flavonoids
1723	2.10E+02	C11H18N2O2	Cyclo(Pro-Leu)*	Amino acids and derivatives
1724	1.64E+02	C9H8O3	Caffeic aldehyde	Phenolic acids
1725	7.13E+02	C30H33O20+	Delphinidin-3-O-(6''-O-malonyl)glucoside-5-O-glucoside	Flavonoids
1726	4.08E+02	C20H24O9	Columbianetin Glucopyranoside	Lignans and Coumarins
1727	4.24E+02	C21H28O9	Grandidentatin	Phenolic acids
1728	8.90E+01	C3H7NO2	L-Alanine	Amino acids and derivatives
1729	4.69E+02	C23H31N7O4	Trp-His-Lys	Amino acids and derivatives
1730	1.82E+02	C9H10O4	2,4-Dihydroxy-6-methoxyacetophenone	Others
1731	3.01E+02	C8H16NO9P	N-Acetyl-D-glucosamine-1-phosphate	Others
1732	4.64E+02	C21H20O12	Quercetin-3-O-glucoside (Isoquercitrin)*	Flavonoids
1733	3.20E+02	C15H12O8	Dihydromyricetin (Ampelopsin)	Flavonoids
1734	6.62E+02	C28H38O18	1-O-rhamnose-3-O-glucoside-Caffeoyl Quinic Acid	Phenolic acids
1735	2.28E+02	C11H16O5	Loganetin	Terpenoids
1736	2.32E+02	C14H16O3	Fraxinellone	Others

1737	7.72E+02	C33H40O21	Quercetin-3-O-sophoroside-7-O-rhamnoside	Flavonoids
1738	2.08E+02	C10H8O5	3-[(1-Carboxyvinyl)oxy]benzoic acid	Phenolic acids
1739	5.05E+02	C24H25O12+	Peonidin-3-O-(6''-O-Acetyl)glucoside	Flavonoids
1740	2.51E+02	C12H13NO5	N-(malonyl)phenylalanine	Amino acids and derivatives
1741	3.86E+02	C15H26N6O6	Pro-Arg-Asp	Amino acids and derivatives
1742	1.80E+02	C9H12N2O2	L-Tyrosinamide	Amino acids and derivatives
1743	3.84E+02	C17H20O10	Fraxidin-8-O-glucoside	Lignans and Coumarins
1744	5.30E+02	C26H26O12	3,4-Di-O-caffeoylquinic acid methyl ester	Phenolic acids
1745	1.92E+02	C10H8O4	Isoscopoletin (6-Hydroxy-7-Methoxycoumarin)	Lignans and Coumarins
1746	3.32E+02	C16H28O7	(3R,4R)-p-Menth-1-ene-3,4-diol-3-O-β-D-glucoside*	Terpenoids
1747	3.32E+02	C16H28O7	(3R,4S,6R)-p-Menth-1-ene-3,6-diol-3-O-β-D-glucoside*	Terpenoids
1748	5.50E+02	C26H30O13	angustiamarin	Terpenoids
1749	2.26E+02	C10H10O6	Chorismic acid	Organic acids
1750	2.46E+02	C10H18N2O5	Leu-Asp	Amino acids and derivatives
1751	4.09E+02	C16H27NO11	Linustatin	Alkaloids
1752	3.20E+02	C16H16O7	Trans-5-O-(p-Coumaroyl)shikimate	Phenolic acids
1753	3.20E+02	C14H16N4O3S	N(4)-Acetylsulfamethazine	Others
1754	1.87E+02	C11H9NO2	3-amino-2-naphthoic acid*	Alkaloids
1755	1.87E+02	C11H9NO2	naphthisoxazol A	Alkaloids
1756	2.23E+02	C11H13NO4	N-Acetyl-L-tyrosine	Amino acids and derivatives
1757	3.68E+02	C17H20O9	3-O-Feruloylquinic acid*	Phenolic acids
1758	4.87E+02	C27H29N5O4	Pro-Trp-Trp	Amino acids and derivatives
1759	3.97E+02	C17H27N5O6	His-Leu-Glu	Amino acids and derivatives
1760	4.44E+02	C20H24N6O4S1	Cys-Trp-His	Amino acids and derivatives
1761	1.92E+02	C10H8O4	6,7-Dihydroxy-4-methylcoumarin	Lignans and Coumarins
1762	3.84E+02	C22H28N2O4	Gambirine*	Alkaloids
1763	3.84E+02	C22H28N2O4	Isogambirine*	Alkaloids
1764	3.90E+02	C17H26O10	Penstemonoside	Terpenoids
1765	3.88E+02	C17H24O10	Epivogeloside	Terpenoids
1766	1.54E+02	C7H6O4	Methyl cumalate*	Phenolic acids
1767	3.70E+02	C21H26N2O4	Rhynchophyllic acid	Alkaloids

1768	3.49E+02	C16H23N5O4	Pro-Pro-His	Amino acids and derivatives
1769	3.86E+02	C22H26O6	Dimethylmatairesinol*	Lignans and Coumarins
1770	3.28E+02	C15H20O8	Androsin	Phenolic acids
1771	1.96E+02	C10H12O4	3-Hydroxy-1-(4-Hydroxy-3-Methoxyphenyl)Propan-1-One	Phenolic acids
1772	3.38E+02	C12H18O11	2-O-α-D-Glucopyranosyl-L-ascorbic acid	Others
1773	2.16E+02	C10H20N2O3	(S)-4-amino-5-oxo-5-(pentylamino)pentanoic acid	Amino acids and derivatives
1774	6.10E+02	C27H30O16	Kaempferol-3,7-O-diglucoside	Flavonoids
1775	3.62E+02	C16H26O9	Glucosyl 5,8-dihydroxy-2,6-dimethylocta-2,6-dienoic acid	Others
1776	7.31E+01	C4H11N	Butylamine	Alkaloids
1777	5.24E+02	C24H28O13	2'-O-(3''-hydroxybenzoyl)-kingiside	Terpenoids
1778	3.32E+02	C13H16O10	1-O-Galloyl-β-D-glucose*	Phenolic acids
1779	6.10E+02	C27H30O16	Quercetin-3-O-neohesperidoside*	Flavonoids
1780	5.60E+02	C27H28O13	3-O-caffeioyl sinapyl quinic acid	Others
1781	3.46E+02	C12H22N6O6	Gly-Arg-Asp	Amino acids and derivatives
1782	4.78E+02	C20H30O13	2,4,6-Trimethoxyphenyl 1-O-β-D-apiofuranosyl-(1→6)-β-D-glucopyranoside	Others
1783	4.90E+02	C24H26O11	3,5-Dihydroxy-3',4'-diacetoxylstilbene-3-O-glucoside	Others
1784	4.32E+02	C21H20O10	Apigenin-6-C-glucoside (Isovitexin)*	Flavonoids
1785	4.32E+02	C21H20O10	Apigenin-8-C-Glucoside (Vitexin)*	Flavonoids
1786	4.62E+02	C21H18O12	Luteolin-7-O-glucuronide	Flavonoids
1787	1.28E+02	C5H8N2O2	Cyclo(Ala-Gly)	Amino acids and derivatives
1788	1.06E+02	C3H6O4	DL-Glyceric Acid	Organic acids
1789	1.66E+02	C9H10O3	Ethylsalicylate	Phenolic acids
1790	1.67E+02	C8H9NO3	Pyridoxal	Others
1791	5.30E+02	C27H34N2O9	Strictosidine	Alkaloids
1792	5.18E+02	C24H38O12	(6S,7E,9R)-vomifoliol-9-O-β-D-Xyl-(1-6)-O-β-D-Glu	Terpenoids
1793	2.90E+02	C10H18N4O6	Argininosuccinic acid	Organic acids
1794	3.54E+02	C20H22N2O4	Mitraphyllic acid	Alkaloids
1795	1.78E+02	C10H10O3	Methyl Hydroxycinnamate	Phenolic acids
1796	5.46E+02	C27H34N2O10	3β-Isodihydrocadambine	Alkaloids
1797	1.26E+02	C6H6O3	Phloroglucinol; 1,3,5-Benzenetriol	Phenolic acids
1798	6.10E+02	C27H30O16	Orientin-7-O-glucoside	Flavonoids
1799	2.50E+02	C13H18N2O3	N-Caffeoylputrescine	Alkaloids

1800	5.80E+02	C28H36O13	Tortoside A	Lignans and Coumarins
1801	4.11E+02	C23H29N3O4	Phe-Val-Phe	Amino acids and derivatives
1802	3.70E+02	C19H30O7	3-(3'-Hydroxybutyl)-2,4,4-trimethylcyclohexa-2,5-dienone glucoside	Terpenoids
1803	3.54E+02	C16H18O9	Scopoletin-7-O-glucoside (Scopolin)	Lignans and Coumarins
1804	2.54E+02	C11H18N4O3	Val-His	Amino acids and derivatives
1805	1.26E+02	C6H6O3	5-Methoxyfurfural*	Others
1806	3.88E+02	C19H32O8	Blumenol B Glucoside*	Terpenoids
1807	1.93E+02	C9H7NO4	5,6-Dihydroxy-1H-indole-2-carboxylic acid	Alkaloids
1808	5.52E+02	C27H36O12	viburfordosides G	Lignans and Coumarins
1809	1.39E+02	C6H5NO3	Nicotinic acid N-oxide	Alkaloids
1810	3.00E+02	C20H28O2	Sugiol	Terpenoids
1811	2.44E+02	C12H24N2O3	L-Leucyl-L-Leucine	Amino acids and derivatives
1812	4.06E+02	C20H22O9	2,3,5,4'-Tetrahydroxystilbene-2-O-glucoside	Others
1813	1.82E+02	C9H10O4	2,6-Dimethoxybenzoic acid	Phenolic acids
1814	4.63E+02	C22H23O11+	Peonidin-3-O-glucoside	Flavonoids
1815	1.40E+02	C7H8O3	Ethyl maltol	Phenolic acids
1816	5.52E+02	C26H32O13	7-O-Caffeoyl Secologanol	Terpenoids
1817	2.93E+02	C17H27NO3	Nonivamide	Alkaloids
1818	7.72E+02	C33H40O21	Quercetin-7-O-rutinoside-4'-O-glucoside	Flavonoids
1819	1.91E+02	C7H13NO3S	N-Acetyl-L-Methionine	Amino acids and derivatives
1820	4.91E+02	C24H29NO10	N-Feruloyloctopamine glucoside	Alkaloids
1821	6.10E+02	C27H30O16	Luteolin-7,3'-di-O-glucoside	Flavonoids
1822	3.90E+02	C20H22O8	Resveratroloside	Others
1823	3.26E+02	C15H18O8	Bilobalide	Terpenoids
1824	3.67E+02	C17H25N3O6	Tyr-Ser-Val	Amino acids and derivatives
1825	5.20E+02	C22H32O14	6'-O-D-Glucosylsweroside	Terpenoids
1826	4.66E+02	C21H22O12	Plantagoside	Phenolic acids
1827	4.04E+02	C17H24O11	6-methoxygeniposidic acid	Others
1828	1.94E+02	C7H14O6	(2r,3s,4s,5r)-2,5-bis(hydroxymethyl)-2-methoxyoxolane-3,4-diol*	Others
1829	2.37E+02	C9H11N5O3	L-Sepiapterin	Nucleotides and derivatives
1830	7.80E+02	C41H64O14	Ruscogenin-1-O-carboxyglucosyl(1,2)rhamnoside	Steroids
1831	4.06E+02	C17H26O11	Shanzhiside methyl ester	Terpenoids

1832	6.22E+02	C30H38O14	Syringaresinol-4'-O-(6''-acetyl)glucoside	Lignans and Coumarins
1833	6.11E+02	C27H31O16+	Cyanidin-3-O-gentiobioside	Flavonoids
1834	4.88E+02	C21H28O13	3'-p-Coumaroyl-sucrose	Phenolic acids
1835	4.00E+02	C22H28N2O5	Rhynchophylline N-oxide	Alkaloids
1836	9.19E+02	C42H47O23+	Delphinidin-3-O-(2'''-O-p-coumaroyl)rutinoside -7-O-glucoside	Flavonoids
1837	3.86E+02	C19H30O8	Citroside A	Terpenoids
1838	2.08E+02	C13H20O2	(R)-3-(3'-Hydroxybutyl)-2,4,4-trimethylcyclohex a-2,5-dienone	Terpenoids
1839	4.12E+02	C15H20N6O8	N6-Threonylcarbamoyladenosine	Others
1840	1.94E+02	C7H14O6	D-Pinitol*	Others
1841	3.09E+02	C17H31N3O2	N1,N10-Ditigloylspermidine	Alkaloids
1842	3.30E+02	C17H14O7	3,7-Di-O-methylquercetin	Flavonoids
1843	4.93E+02	C20H31NO13	Pyridoxine-5'-O-diglucoside	Others
1844	6.11E+02	C30H27O14+	Delphinidin-3-O-(6''-O-p-coumaroyl)glucoside	Flavonoids
1845	1.26E+02	C6H6O3	5-Hydroxymethylfurfural*	Others
1846	6.22E+02	C29H34O15	Crenatoside	Phenolic acids
1847	3.88E+02	C15H28N6O6	Asp-Arg-Val	Amino acids and derivatives
1848	7.40E+02	C33H40O19	Kaempferol-3-O-robinoside-7-O-rhamnoside (Robinin)	Flavonoids
1849	6.10E+02	C27H30O16	Quercetin-3-O-glucoside-7-O-rhamnoside*	Flavonoids
1850	6.10E+02	C27H30O16	Quercetin-7-O-rutinoside*	Flavonoids
1851	5.20E+02	C26H32O11	Dehydrodiconiferyl alcohol-gamma'-O-glucoside	Lignans and Coumarins
1852	4.78E+02	C21H18O13	3,4-Digalloylshikimic acid	Phenolic acids
1853	4.64E+02	C21H20O12	Quercetin-5-O-β-D-glucoside*	Flavonoids
1854	3.21E+02	C16H23N3O4	Gly-Val-Phe	Amino acids and derivatives
1855	2.60E+02	C11H20N2O5	gamma-Glu-leu	Amino acids and derivatives
1856	5.22E+02	C26H34O11	Lariciresinol-4'-O-glucoside	Lignans and Coumarins
1857	5.22E+02	C26H34O11	Icariside E5	Others
1858	4.04E+02	C17H24O11	Secoxyloganin	Terpenoids
1859	3.42E+02	C20H22O5	Avicennol	Lignans and Coumarins
1860	4.64E+02	C21H20O12	Quercetin-3-O-alloside; Isohyperoside*	Flavonoids
1861	4.64E+02	C21H20O12	Quercetin-3-O-galactoside (Hyperin)*	Flavonoids
1862	1.42E+02	C8H14O2	2-n-Propyl-2-pentenoic acid	Organic acids
1863	2.08E+02	C11H12O4	Methyl 3-(4-methoxyphenyl)-2-oxopropanoate	Others
1864	5.57E+02	C29H39N3O8	N1,N8-Bis(sinapoyl)spermidine	Alkaloids
1865	3.01E+02	C18H23NO3	Dobutamine	Alkaloids

1866	1.64E+02	C9H8O3	α-Hydroxycinnamic Acid*	Phenolic acids
1867	2.84E+02	C15H24O5	Dendrobiumane D	Others
1868	3.58E+02	C16H22O9	Sweroside	Terpenoids
1869	1.66E+02	C9H10O3	4'-Hydroxy-3'-methoxyacetophenone (Acetovanillone)	Phenolic acids
1870	5.94E+02	C27H30O15	Isosaponarin(Isovitexin-4'-O-glucoside)	Flavonoids
1871	5.64E+02	C26H28O14	Isoschaftoside	Flavonoids
1872	5.52E+02	C27H36O12	Tinosposinenside A	Terpenoids
1873	1.32E+02	C6H12O3	6-Hydroxyhexanoic acid	Organic acids
1874	1.52E+02	C10H16O	Perillyl alcohol	Terpenoids
1875	2.51E+02	C13H17NO4	Ethyl N-acetyl-L-tyrosinate	Amino acids and derivatives

Table S2. DEGs-based KEGG metabolic pathway enrichment in comparison of B vs CK.

Number	Kegg_pathway	Ko_id	P-value
1	Anthocyanin biosynthesis	ko00942	0.07
2	Carotenoid biosynthesis	ko00906	0.08
3	Flavone and flavonol biosynthesis	ko00944	0.09
4	Thiamine metabolism	ko00730	0.16
5	Phenylpropanoid biosynthesis	ko00940	0.18
6	Biotin metabolism	ko00780	0.23
7	Benzoxazinoid biosynthesis	ko00402	0.23
8	Stilbenoid, diarylheptanoid and gingerol biosynthesis	ko00945	0.30
9	Flavonoid biosynthesis	ko00941	0.30
10	Monoterpenoid biosynthesis	ko00902	0.36
11	Indole alkaloid biosynthesis	ko00901	0.36
12	Phenylalanine metabolism	ko00360	0.36
13	Cysteine and methionine metabolism	ko00270	0.40
14	Oxidative phosphorylation	ko00190	0.41
15	Sulfur metabolism	ko00920	0.41
16	Linoleic acid metabolism	ko00591	0.46
17	Tryptophan metabolism	ko00380	0.46
18	Biosynthesis of various plant secondary metabolites	ko00999	0.51
19	Biosynthesis of secondary metabolites	ko01110	0.54
20	Pantothenate and CoA biosynthesis	ko00770	0.59
21	Biosynthesis of various alkaloids	ko00996	0.59
22	Arginine biosynthesis	ko00220	0.62
23	Nicotinate and nicotinamide metabolism	ko00760	0.74
24	Arginine and proline metabolism	ko00330	0.78
25	Tyrosine metabolism	ko00350	0.78
26	2-Oxocarboxylic acid metabolism	ko01210	0.94
27	Biosynthesis of amino acids	ko01230	0.95
28	Biosynthesis of cofactors	ko01240	0.96
29	ABC transporters	ko02010	0.98
30	Metabolic pathways	ko01100	1.00

Table S3. DEGs-based KEGG metabolic pathway enrichment in comparison of G vs CK.

Number	Kegg_pathway	Ko_id	P-value
1	Anthocyanin biosynthesis	ko00942	0.01
2	Zeatin biosynthesis	ko00908	0.02
3	Purine metabolism	ko00230	0.06
4	Oxidative phosphorylation	ko00190	0.06
5	Photosynthesis	ko00195	0.07
6	Stilbenoid, diarylheptanoid and gingerol biosynthesis	ko00945	0.13

7	Glycosylphosphatidylinositol (GPI)-anchor biosynthesis	ko00563	0.16
8	Carotenoid biosynthesis	ko00906	0.16
9	Biosynthesis of cofactors	ko01240	0.18
10	Nucleotide metabolism	ko01232	0.18
11	Neomycin, kanamycin and gentamicin biosynthesis	ko00524	0.19
12	Glycerophospholipid metabolism	ko00564	0.25
13	Biosynthesis of nucleotide sugars	ko01250	0.25
14	Amino sugar and nucleotide sugar metabolism	ko00520	0.25
15	Thiamine metabolism	ko00730	0.30
16	Porphyrin metabolism	ko00860	0.30
17	Lysine degradation	ko00310	0.31
18	Tryptophan metabolism	ko00380	0.33
19	Phenylpropanoid biosynthesis	ko00940	0.33
20	Ascorbate and aldarate metabolism	ko00053	0.41
21	Biotin metabolism	ko00780	0.41
22	Folate biosynthesis	ko00790	0.41
23	Benzoxazinoid biosynthesis	ko00402	0.41
24	Butanoate metabolism	ko00650	0.45
25	Lysine biosynthesis	ko00300	0.45
26	Galactose metabolism	ko00052	0.50
27	C5-Branched dibasic acid metabolism	ko00660	0.50
28	Carbon fixation in photosynthetic organisms	ko00710	0.50
29	Histidine metabolism	ko00340	0.50
30	Caffeine metabolism	ko00232	0.51
31	Glycine, serine and threonine metabolism	ko00260	0.56
32	Glycerolipid metabolism	ko00561	0.59
33	Sphingolipid metabolism	ko00600	0.59
34	Monoterpenoid biosynthesis	ko00902	0.59
35	Indole alkaloid biosynthesis	ko00901	0.59
36	Isoquinoline alkaloid biosynthesis	ko00950	0.59
37	Sulfur metabolism	ko00920	0.66
38	Vitamin B6 metabolism	ko00750	0.66
39	Monobactam biosynthesis	ko00261	0.66
40	Citrate cycle (TCA cycle)	ko00020	0.72
41	Pyruvate metabolism	ko00620	0.72
42	Ubiquinone and other terpenoid-quinone biosynthesis	ko00130	0.72
43	Phenylalanine metabolism	ko00360	0.73
44	Pentose phosphate pathway	ko00030	0.76
45	Pentose and glucuronate interconversions	ko00040	0.76
46	Metabolic pathways	ko01100	0.78
47	Arginine and proline metabolism	ko00330	0.80
48	Tyrosine metabolism	ko00350	0.80
49	Starch and sucrose metabolism	ko00500	0.80
50	Linoleic acid metabolism	ko00591	0.82

51	Aminoacyl-tRNA biosynthesis	ko00970	0.85
52	beta-Alanine metabolism	ko00410	0.86
53	Glutathione metabolism	ko00480	0.86
54	Biosynthesis of secondary metabolites	ko01110	0.88
55	D-Amino acid metabolism	ko00470	0.89
56	Glyoxylate and dicarboxylate metabolism	ko00630	0.89
57	Valine, leucine and isoleucine biosynthesis	ko00290	0.89
58	ABC transporters	ko02010	0.89
59	Carbon metabolism	ko01200	0.90
60	Flavonoid biosynthesis	ko00941	0.90
61	Flavone and flavonol biosynthesis	ko00944	0.90
62	Pyrimidine metabolism	ko00240	0.93
63	Nicotinate and nicotinamide metabolism	ko00760	0.93
64	Cysteine and methionine metabolism	ko00270	0.95
65	Biosynthesis of amino acids	ko01230	0.98
66	2-Oxocarboxylic acid metabolism	ko01210	1.00

Table S4. DEGs-based KEGG metabolic pathway enrichment in comparison of R vs CK.

Number	Kegg_pathway	ko_ID	P-value
1	Anthocyanin biosynthesis	ko00942	0.01
2	Propanoate metabolism	ko00640	0.02
3	Biotin metabolism	ko00780	0.02
4	Linoleic acid metabolism	ko00591	0.06
5	Phenylpropanoid biosynthesis	ko00940	0.06
6	alpha-Linolenic acid metabolism	ko00592	0.06
7	Lysine degradation	ko00310	0.08
8	Vitamin B6 metabolism	ko00750	0.09
9	Phenylalanine metabolism	ko00360	0.13
10	Biosynthesis of various alkaloids	ko00996	0.21
11	Fatty acid degradation	ko00071	0.24
12	Alanine, aspartate and glutamate metabolism	ko00250	0.28
13	Stilbenoid, diarylheptanoid and gingerol biosynthesis	ko00945	0.30
14	Flavonoid biosynthesis	ko00941	0.31
15	Indole alkaloid biosynthesis	ko00901	0.37
16	Pyrimidine metabolism	ko00240	0.38
17	Oxidative phosphorylation	ko00190	0.42
18	Sulfur metabolism	ko00920	0.42
19	Citrate cycle (TCA cycle)	ko00020	0.47
20	Pyruvate metabolism	ko00620	0.47
21	Valine, leucine and isoleucine degradation	ko00280	0.47
22	Pentose phosphate pathway	ko00030	0.52

23	Biosynthesis of cofactors	ko01240	0.55
24	Butanoate metabolism	ko00650	0.56
25	Lysine biosynthesis	ko00300	0.56
26	Arginine biosynthesis	ko00220	0.63
27	Glyoxylate and dicarboxylate metabolism	ko00630	0.67
28	Flavone and flavonol biosynthesis	ko00944	0.70
29	Ascorbate and aldarate metabolism	ko00053	0.72
30	Biosynthesis of secondary metabolites	ko01110	0.72
31	Nicotinate and nicotinamide metabolism	ko00760	0.75
32	Tyrosine metabolism	ko00350	0.79
33	Purine metabolism	ko00230	0.81
34	Tryptophan metabolism	ko00380	0.81
35	Biosynthesis of various plant secondary metabolites	ko00999	0.84
36	Carbon metabolism	ko01200	0.87
37	Nucleotide metabolism	ko01232	0.89
38	2-Oxocarboxylic acid metabolism	ko01210	0.95
39	Biosynthesis of amino acids	ko01230	0.95
40	ABC transporters	ko02010	0.99
41	Metabolic pathways	ko01100	1.00

Supplementary Figure S1: Emission spectra of the LED light sources used in this study.

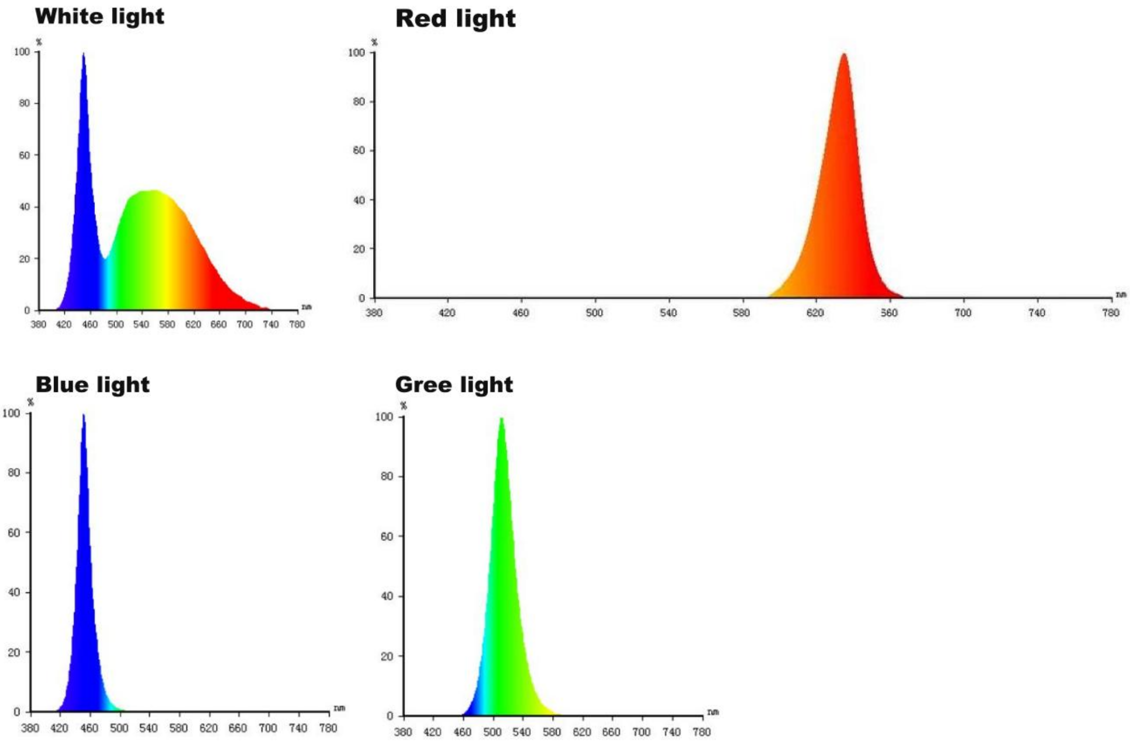

Figure S2. MRM Metabolite Detection Multi-Peak Chart

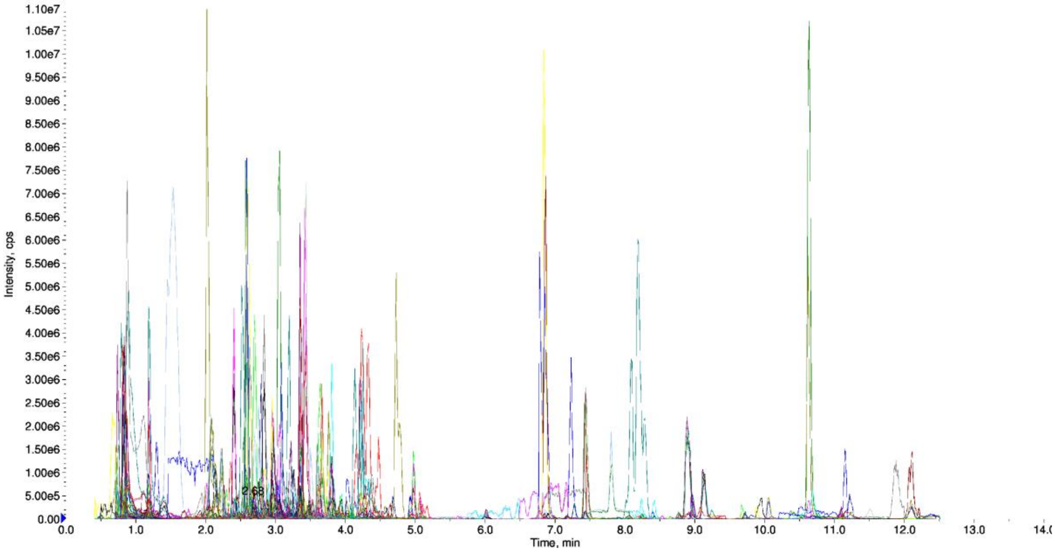

negative

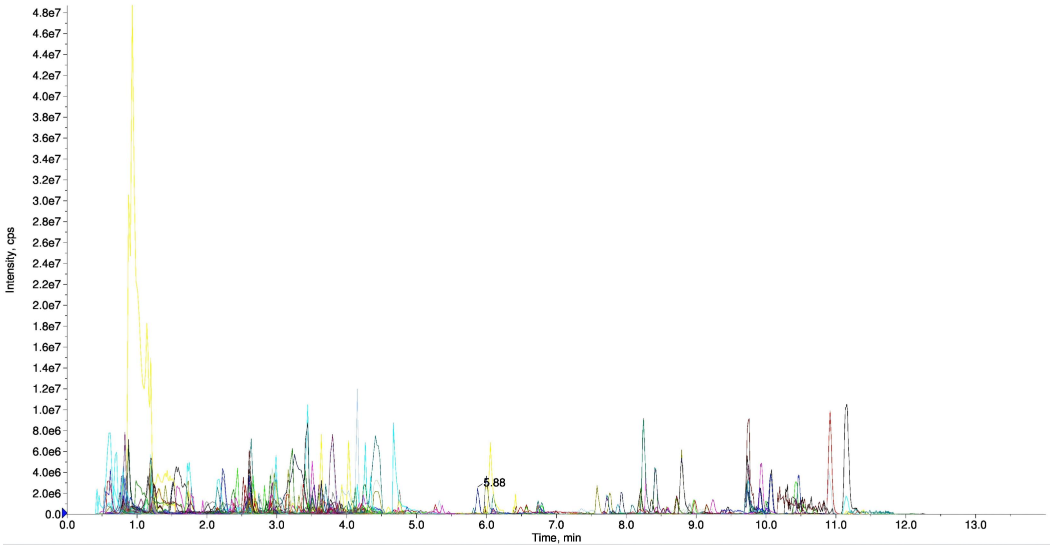

Supplement: Supplementary file 1 [file plants-14-03268-s001.zip › plants-3912947-supplementary.pdf]
